# Magnetic resonance identification tags for ultra-flexible electrodes

**DOI:** 10.1038/s41467-026-71887-x

**Published:** 2026-04-28

**Authors:** Eminhan Özil, Peter Gombkoto, Athina Apostolelli, Tansel Baran Yasar, Angeliki D. Vavladeli, Markus Marks, Manabu Rohr-Fukuma, Wolfger von der Behrens, Mehmet Fatih Yanik

**Affiliations:** 1 Neurotechnology Group, Institute of Neuroinformatics, Department of Information Technology and Electrical Engineering, ETH Zurich and University of Zurich, Zurich, Switzerland; 2 Neuroscience Center Zurich, University of Zurich and ETH Zurich, Zurich, Switzerland; 3 Sainsbury Wellcome Centre for Neural Circuits and Behaviour, University College London, London, UK; 4 Brain Research Institute, University of Zurich, Zurich, Switzerland; 5 University Research Priority Program (URPP), Adaptive Brain Circuits in Development and Learning, University of Zurich, Zurich, Switzerland; 6 Center for Microscopy and Image Analysis (ZMB), University of Zurich, Zurich, Switzerland; 7 Division of Computing and Mathematical Sciences, Caltech, Pasadena, CA USA; 8 Division of Engineering and Applied Science, Caltech, Pasadena, CA USA; 9 Department of Neurosurgery, University Hospital Zurich, University of Zurich, Zurich, Switzerland; 10 Clinical Neuroscience Center, University Hospital Zurich, University of Zurich, Zurich, Switzerland

**Keywords:** Neuroscience, Biomedical engineering, Nanoparticles, Magnetic devices, Implants

## Abstract

Ultra-flexible electrodes, due to their superior biocompatibility, are likely to lead the future of neuroprosthetics. However, identifying the precise positions of implanted high-density ultra-flexible electrodes in the brain for accurately assigning neural signals to specific structures remains a major challenge. To address this, we developed magnetic resonance identification (MRID)-tags. Each ultra-flexible electrode bundle carries an MRID-tag with unique barcode patterns visible in MRI (MRI-barcodes) for identification of the bundle. Individual bars in MRI-barcodes allow an accurate 3D reconstruction of the ultra-flexible electrode bundle’s trajectory in the brain and determine the anatomical positions of individual electrodes. We generate the MRI-barcodes by patterning superparamagnetic iron-oxide nanoparticles into electrode fibers (10 µm^2^) with dot-matrix nanoparticle coating technique. We chronically tested MRID-tagged ultra-flexible electrodes in vivo in the dorsal hippocampus of freely-moving rats, where distinct electrophysiological landmarks validated our electrode localization results. We were able to localize individual electrodes with a mean accuracy of 95 μm. MRID-tagged ultra-flexible electrodes demonstrated high long-term recording stability with mean single-unit signal-to-noise ratios as high as 20.

## Abstract (web summary)

Flexible electrodes offer higher biocompatibility but remain difficult to locate inside the brain, limiting data interpretation and targeting. Here, the authors demonstrate electrodes with magnetic tags for identification and localization.

## Introduction

High-density distributed single-neuron resolution electrophysiology is an important tool for understanding functions of brain circuits and mechanisms of brain disorders in basic research, and for improving the quality of diagnosis and therapeutics in the clinic^1–6^. Various electrophysiological probes have been developed with different mechanical/material properties, geometries, and electronics to achieve high-density distributed electrophysiology^3,7,8^. Flexible probes are increasingly favored for their minimal footprint and improved biocompatibility^8–10^. Stiff probes (e.g., silicon probes) often cause significant glial scarring^11^, and recording drifts due to brain micro-motions^7^. This reduces the signal-to-noise ratio (SNR) of recordings and the total number of recorded single neurons over time. In contrast, flexible probes ensure stable recording over long durations^9^.

Identifying the precise locations of implanted electrodes in the brain is important to accurately interpret the recorded data and to stimulate only desired brain structures. However, error-prone surgical procedures, subject-to-subject morphological variance, and vasculatures through the implantation trajectory introduce error and variance to each electrode implantation. For example, in human microwire recordings (guided through clinical depth electrodes), microwires randomly spread into the tissue as they are inserted, where the precise location of each electrode channel remains unknown, leading to major uncertainties in the interpretation of the exact brain structure and functional associations^12,13^. The variance and error increase with high-density distributed implantations.

For post-mortem analysis (typically in small animal models), optical microscopy can be used to visualize electrode trajectories after coating the probes with fluorescent dyes prior to implantation^14^ or performing electrolytic lesions. However, both methods yield only qualitative results due to dye diffusion and lesion sizes. Fluorescent barcodes incorporated to the electrodes have been proposed to achieve high-spatial resolution of electrode locations^15^, yet this requires electrodes to remain in place during tissue processing, which is too challenging, especially during slicing.

In clinical and non-human primates (NHP) studies, noninvasive imaging techniques such as computed tomography (CT) and magnetic resonance imaging (MRI) are essential for in vivo electrode localization^16–18^. While CT is more commonly used intra-/post- operatively, it lacks the soft tissue contrast and functional mapping that MRI provides. Existing clinical or NHP electrodes are readily visible in CT and MRI scans because of their large geometry and metal contacts, yet imaging markers on electrodes are still necessary for accurate electrode localization. For example, specific-geometry radiopaque markers on mm-size deep brain stimulation probes are utilized to determine the orientation of bi-directional stimulation electrodes in CT scans^19^. However, the micron-scale footprints of ultra-flexible electrodes make it impossible to use such CT and MRI methods to identify electrode locations in the brain. Yet, a reliable in vivo localization technique for ultra-flexible electrodes is essential to translate them to clinical practice.

**Fig. 1:**
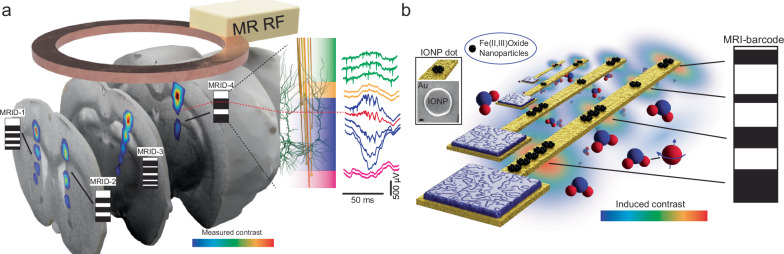
Magnetic resonance identification (MRID) tags allow high-resolution identification of ultra-flexible electrodes in the brain by MRI-barcodes. **a** MRID-tagged multiple ultra-flexible tentacle electrodes (UFTEs) implanted for distributed recordings. Each UFTE bundle has an MRID-tag with a unique MRI-barcode for identification. MRID-tags, furthermore, carry information about the precise positions of the individual electrode channels within the brain. The inset image shows an illustrative UFTE bundle registered to anatomical regions (the colors encode different anatomy) with illustrative raw electrophysiology traces. **b** Iron-oxide nanoparticles (IONPs) are wafer-level patterned on ultra-flexible electrode wires as a dot-matrix. The scanning electron microscope (SEM) image shows a sample IONP dot where IONPs are clustered circularly. IONP patterns on electrode wires induce unique barcode-like patterns locally in structural MRI images by reducing the T2* relaxation time of neighboring water protons. Scale bar denotes 200 nm in the SEM image in (**b**).

Here, we developed magnetic resonance identification (MRID) tags to uniquely identify ultra-flexible electrode shanks while also localizing individual electrode channels in MRI scans. MRID-tags (made of superparamagnetic nanoparticles) generate custom barcode patterns visible in MRI images unique to each ultra-flexible electrode shank, which we call *MRI-barcodes* (Fig. 1a, see Results). After the identification of an ultra-flexible shank using MRI-barcode, the individual bars along the MRI-barcode allow an accurate 3D reconstruction of the ultra-flexible shank trajectory. Subsequently, we localize the individual electrode channels along the reconstructed shank trajectory by using their fabricated distances relative to the patterned MRI-barcodes (Fig. 1a), with mean 95 µm accuracy validated electrophysiologically (see Results).

## Results

MRID-tags are made of MRI (T2/T2*-weighted) contrast agents i.e., superparamagnetic iron(II, III)-oxide nanoparticles (IONPs)^20–23^ (Fig. 1b). MRI contrast agents are substances (e.g., IONP and gadolinium etc.) that change the local magnetic properties to enhance the image contrast to identify specific anatomies and pathologies. We coat IONPs into individual ultra-flexible electrode fibers in unique barcode-like patterns as dot-matrices (Fig. 1b). Each dot in the matrix is an assembly of thousands of IONPs, as shown in Fig. 1b. The IONP dot-matrix alters the local magnetic properties of neighboring tissue, thereby yielding MRI-barcodes.

We tested the MRID-tags embedded in 64-channel (50 µm center-to-center spacing between recording sites) ultra-flexible tentacle electrodes (UFTEs)^9^ chronically implanted into rat brains to assess MRI-barcodes and electrode localization in vivo. UFTEs offer arbitrary depth and angle penetration capability, a minimal electrode footprint, high-density and distributed recording sites, high SNR, and months-long brain activity recordings facilitated by a novel implantation technique^9^ (Supplementary Fig. 1). We used Waxholm space (WHS) rat brain reference atlas for anatomical labeling^24^. The electrode localization accuracy was validated against established dorsal hippocampal (dHPC) electrophysiological landmarks—namely the pyramidal layer (PyL) and hippocampal fissure (HF) identified from local field potential profiles, with ripple power (150–200 Hz) components of sharp wave ripple (SWR) peaking in the pyramidal layer and theta power (7 Hz) reaching a maximum near the HF^6,25–31^.

### Dot-matrix nanoparticle coating for MRID-tag fabrication

**Fig. 2:**
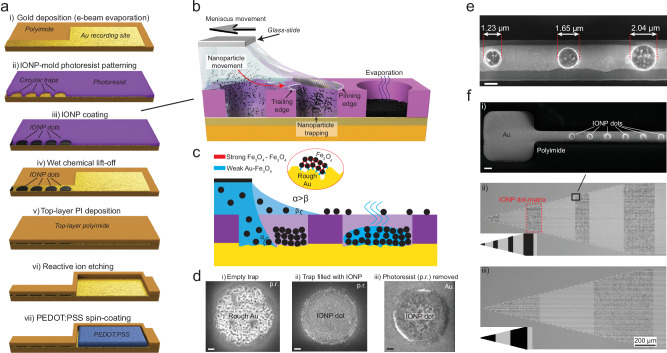
Microfabrication and characterization of ultra-flexible electrodes with MRI-barcodes. **a** Microfabrication steps from top to bottom: gold deposition, photoresist patterning to form recessed dot-matrix template for IONP coating, IONP coating, photoresist and excess IONP lift-off, polyimide deposition, polyimide etching for mechanically independent fibers and exposed Au surface at recording site, PEDOT:PSS coating onto recording sites. **b** Dot-matrix nanoparticle coating explained. IONP colloidal suspension between the glass slide and substrate forms a meniscus. The meniscus drags over the dot-matrix. Nanoparticles migrate into the dot-matrix traps due to laminar flows. **c** Hybrid meniscus explained. Roughened porous Au surface is hydrophobic and makes a larger contact angle (*σ* = ~40–60°, *β* = ~20°) with the meniscus. Grooves act as vertical confinement, yielding a capillary assembly. Porous surface increases the surface area between the Au surface and Fe_3_O_4_ (i.e., IONPs). Excess water evaporates, constructing a multi-layer IONP assembly in disk shape. **d** SEM images show the IONP coating steps. (i) Empty dot trap defined by patterned photoresist (p.r.) layer. The exposed Au surface is roughened chemically. (ii) The dot trap is filled with IONP after the coating. (iii) P.r. layer is lifted off together with excess IONP. IONP dot remains on the gold surface. **e** SEM image shows the different sizes of IONP dots tested, 1.25, 1.65, and 2.00 µm in diameter. **f** IONP coating alignment and patterning results. SEM image of a circular IONP assembly shown aligned on Au wire (panel i). The panels ii, iii show dot-matrix patterns on UFTE bundles. A pattern with 4-bars (panel ii) and a pattern with 2-bars (panel iii) are shown from optical microscope images, where the insets (bottom-left for each microscopy image) show the actual design layouts. Scale bars, 200 nm in (**d**); 1 µm in (**e**); 2 µm in (**f**) panel i.

We wafer-scale microfabricate UFTEs^9^ with MRID-tags as illustrated in Fig. 2a (see Methods). To achieve precisely coated IONP dots which construct MRID-tags, we adapted a dot-matrix nanoparticle coating technique based on the topography-controlled capillary and convective assembly (CCA)^32–34^ with a dragging meniscus of nanoparticle colloidal suspension. Convective assembly relies on evaporation-driven convective flow at low contact angles to deposit continuous nanoparticle layers, whereas capillary assembly uses meniscus pinning and capillary forces at higher contact angles to selectively trap and deposit particles in confined regions^32–34^. Our dot-matrix coating technique employs a dragging meniscus similar to that used in CCA^32–34^, which drives the nanoparticles toward the pinning edge via convective flows^32–34^. A photoresist layer is used to define dot-matrix traps for IONPs, enabling topography-controlled assembly. These traps capture large quantities of IONPs directly on the target substrate, eliminating the need for an intermediate stamp^32–34^.

First, we deposit a gold (Au) layer on polyimide to construct electrode contact sites, wire tracks, and solder pads (Fig. 2ai, see Methods). We pattern a thin photoresist layer on the sample to define the dot-matrix traps (~2 µm dot diameter), which are aligned and positioned on the Au wire tracks (Fig. 2aii). We coat IONP (25 nm nanoparticle diameter) in the dot-matrix traps, which are defined by the photoresist layer (Fig. 2aiii), as described in the next paragraph.

We start the dot-matrix coating by injecting a fixed amount of IONP colloidal suspension in deionised ultrapure water (dH_2_O), which covers all the UFTE fibers to be coated, in between the sample and the horizontal glass slide (Fig. 2b). The sample is moved linearly at a constant speed in the direction of the leading edge of the colloid meniscus, creating a dragging movement of the meniscus over the dot-matrix traps on the sample (Fig. 2b) using a custom-made setup (Supplementary Fig. 2a). The meniscus pins at the upper-edge of the trap while the nanoparticles are accumulated in the trap due to laminar flows^32,33^ (Fig. 2b). The trailing edge of the trap prevents nanoparticles from escaping (Fig. 2b). When the pinning breaks, the meniscus fully detaches from the corresponding trap, leaving a fixed volume of colloidal suspension in the trap. As the water evaporates, a micro-scale drop-casting-like assembly happens. Our results show that IONPs in each dot form a crater shape (Supplementary Fig. 2b). This suggests that a drop-casting-like assembly is occurring in circular traps with the trap diameters we used in this work. We have optimized the meniscus speed and the concentration of IONP colloidal suspension to prevent an IONP bridge formation from inside the circular trap to the outer surface of the photoresist (Supplementary Fig. 2c).

We hypothesize that sufficiently large traps allow the meniscus to partially contact the underlying Au surface after breaking from the photoresist pinning edge (Fig. 2c). We observed results suggesting this was the dominant mechanism when we used elongated traps (e.g., strips) in the matrix instead of dots (Supplementary Fig. 2d). We observe that the porous Au surface is more hydrophobic with increased contact angles varying between 40–60° (Fig. 2c). Possibly, a capillary dominant assembly occurs at that contact angle so that the porous structure acts as a capillary trap. Finally, the trailing upper-edge of the photoresist breaks the meniscus, leaving a fixed volume of colloidal suspension in the trap, resulting in a local drop-casting-like assembly.

After the coating, to remove the excess IONP, we chemically (i.e., wet lift-off) remove the photoresist layer (Fig. 2aiv). According to our observations, a low-vapor pressure (i.e., less-volatile) organic solvent is necessary to re-disperse excess nanoparticles following the dissolution of the photoresist to prevent excess nanoparticles from adhering to undesired locations on the substrate (see Methods). We apply gentle mechanical agitations to help the re-dispersion of excess IONPs in the organic solvent during lift-off. We achieve a clean lift-off with this method, which leaves the IONP dots only on the desired spots on the substrate, as observed in the SEM images in Fig. 2d.

We evaluated the IONP coating and lift-off on different substrates, such as smooth Au (i.e., e-beam evaporated, nanometer scale grains) and spin-coated polyimide (Supplementary Fig. 2e). On smooth surfaces, lift-off was less reliable, as IONP dots occasionally detached. To improve the lift off, before the IONP coating, we chemically roughen the exposed gold surface at the traps (see Methods), forming grooves with sizes within 100 nm (Fig. 2di). The rough surface increases the effective surface area for weaker Au-Fe_3_O_4_ bonds compared to Fe_3_O_4_-Fe_3_O_4_ bonds (Fig. 2c inset). The larger effective surface area increases the adhesion of IONP dots to the underlying Au surface, which yields a more reliable lift-off. Although we haven’t tested it in this work, to improve the lift-off on polyimide, similarly, the surface can be roughened and treated (e.g., via reactive ion etching).

We can construct custom geometries of nanoparticle assemblies on the sample using dot-matrix coating. We tested coating IONP dots in different diameter circular traps 1 µm, 1.25 µm, and 1.50 µm dots on photomask, slight lateral enlargement is observed due to development (1.25, 1.7 and 2 µm after development), as shown in Fig. 2e. Coated IONP dots are well aligned with the existing Au wires (Fig. 2fi). In Figure 2f (panels ii and iii), the microscopy images show barcode patterns formed by the IONP dot-matrices where the inset images show the actual design layouts for each barcode pattern.

As the final steps, we coat the top polyimide layer to encapsulate the IONP and insulate the Au (Fig. 2av). We etch the polyimide to construct the mechanically independent fibers and expose the contact sites (Fig. 2avi). Finally, PEDOT:PSS is coated onto the contact sites to lower the electrical electrode-tissue impedance (see Methods).

### MRI characterization and channel mapping

**Fig. 3:**
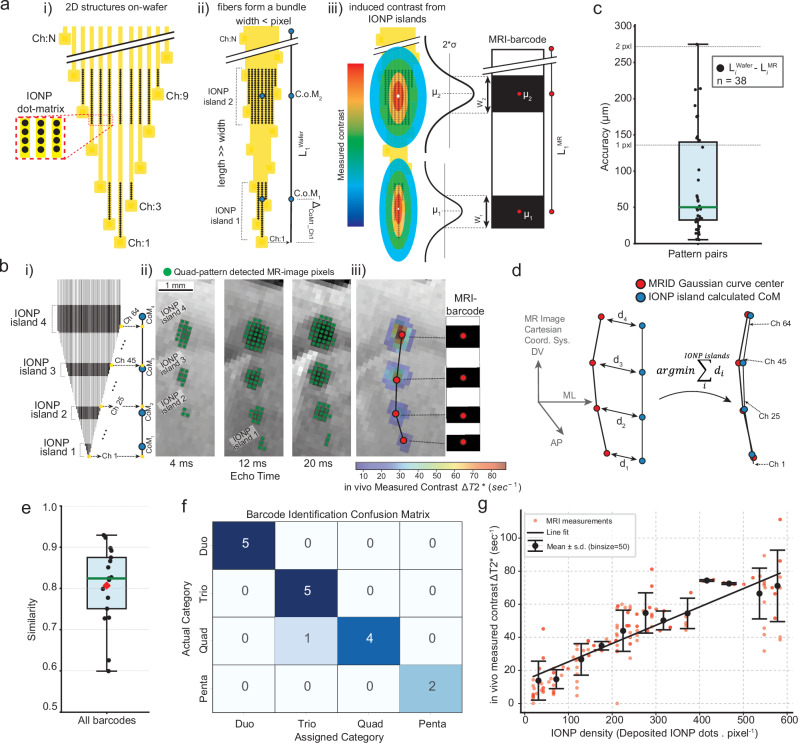
Localization of MRID-tagged electrode channels within the brain by MRI. **a** Left-to-right: IONP dot-matrices (*black*) on Ti/Au (*yellow*) wires. IONP island centers-of-masses (CoMs) (*blue* dots) are calculated from the 2D layout. CoMs define the 1D track. IONP islands induce MRI contrasts centered at the CoMs. Gaussian curves are fitted onto measured contrasts. Curve centers (*red* dots) reconstruct the trajectory of the bundle in MRI-space. MRID-barcode is reconstructed with the fitted curve centers (µ_i_) and 2σ (σ: sigma of the Gaussian curve fit) in the longitudinal axis. **b**-(i) 2D layout of Ti/Au tracks (*light gray*) and IONP islands (*black*) of an example MRID-tag. CoMs (*blue* dots) were calculated, which construct the 1D track. Channels were mapped onto the 1D track, four exemplary channels are shown with yellow rectangles (ch- 1, 25, 45, 64). (ii) In vivo induced contrast from IONP islands at increasing echotimes. Contrastive pixels are detected (z-score pixel intensity < −2, *n* > 100 pixels in baseline anatomical region). (iii) MRI contrast by MRID-tags is shown as a heatmap, 2D Gaussian curves are fitted on each island. The trajectory and MRID barcode are reconstructed. **c** Accuracy of reconstructed bundles (*n* = 38 IONP island pairs, minimum = 5.29, maximum = 424.29, median = 50.15, denoted by green line, 25th percentile = 32.53, 75th percentile = 140.16, IQR = 107.63), data are jittered along the x-axis for better visibility. **d** Channel mapping explained. The reconstructed trajectory (*red* dots) is in 3D MRI space. The calculated trajectory is initially positioned arbitrarily in the MRI-space. Calculated trajectory is fitted onto the reconstructed trajectory. Channels are linearly interpolated along the best-fit trajectory with their relative distances to calculated CoMs. **e** Boxplot for similarity scores of MRI-barcodes (minimum = 0.60, maximum = 0.93, median = 0.82 denoted by green line, mean = 0.81 denoted by red diamond, 25th percentile = 0.75, 75th percentile = 0.88). Data are jittered along the x-axis for better visualization (*n* = 17 MRI-barcodes). **f** Confusion matrix for MRI-barcode assignments. 94.11% of MRI-barcodes were assigned to the correct category (*n* = 17 MRI-barcodes). **g** In vivo measured MRI contrast levels (mean ± SD, *n* = 8 MRID-tags) as a function of IONP coating density showing the linear correlation with best-fit line (*black*).

UFTEs are MRI compatible and safe due to their material properties and geometries, which make them suitable for this work to test the MRID-tags. First, we imaged the IONP-coated UFTE bundles in vitro implanted in MRI phantoms in 7 T and 3 T scanners to show that IONP coating enhances the visibility of UFTE bundles (see Methods, Supplementary Fig. 3) while uncoated UFTE bundles were fully invisible. Then, to ensure that we can in vivo test MRID-tagged UFTE bundles safely, we carried out full-wave COMSOL simulations to show there is no radio-frequency induced heating (see Methods, Supplementary Fig. 4). Moreover, we expect that the small transverse gold surfaces in UFTEs induce minimal undesired Eddy current under changing gradient magnetic fields in MRI. In vitro tests and simulations confirmed that we can safely test MRID-tagged UFTE bundles in vivo in alive animals. Figure 3a illustrates the MRID-tag concept. IONPs are wafer-scale coated on Au wires in dot-matrices to form barcode patterns in two-dimensional (2D) layouts (Fig. 3a i). When we remove UFTEs from the wafer, individual fibers collapse into a linear bundle^9^ (~65 µm bundle thickness for 64 fibers, Fig. 3a ii, Supplementary Fig. 5). Bundle formation reduces the lateral gap between IONP dots within individual fibers, resulting in a three-dimensional cluster where both lateral and vertical dot-to-dot separations are less than ~5 µm. Because this separation (< ~5 μm) is nearly two orders of magnitude smaller than the dimensions of an MRI voxel (pixel size: 136 µm × 136 µm, slice-thickness: 800 µm), these clustered IONP dots can be approximated as continuous, solid-body assemblies – hereafter referred to as *IONP islands* – within the spatial resolution limits of MRI (Fig. 3a ii). The center of mass (CoM) of each IONP island can be accurately determined from the two-dimensional design layout (Fig. 3a ii). The longitudinal extent of the IONP islands exceeds the bundle thickness by approximately an order of magnitude. Under these assumptions, we can approximate the UFTE bundles as one-dimensional (1D) trajectories represented by their CoMs in MRI images. 

To observe the temporal dynamics of induced MRI contrast, we imaged the T2*-weighted contrast by MRID-tags (see Methods) at increasing echo times (TEs), defined as the interval between the excitation pulse and the peak of the measured MR signal. T2*-weighted MRI is sensitive to both intrinsic T2 relaxation, which reflects spin-spin interactions influenced by tissue microstructure and water content, and dephasing effects caused by magnetic field inhomogeneities. IONP islands act as strong local sources of magnetic field inhomogeneity, enhancing the image contrast. IONP islands induce spatially continuous contrast patterns, with the contrast intensity determined by the density of IONP dots within the IONP island and distance from the IONP island (Fig. 3a). Induced continuous contrasts are well described by two-dimensional Gaussian curves with the peak contrast intensity centered at the CoM of each IONP island. We fit 2D Gaussian curves to the contrast of individual IONP islands to estimate their CoM positions in MRI (i.e., curve centers of 2D Gaussian fits, µ_i_) in MRI (Fig. 3a). We reconstructed the MRI-barcodes starting from the deepest (i.e., ventral-most) IONP island, where each bar is centered at the estimated CoM and assigned with a width of 2σ (σ: sigma of the Gaussian curve fit) of the corresponding best-fit Gaussian (Fig. 3a, see Methods).

We designed randomly generated proof-of-principle MRI-barcodes with 300 µm pitch (i.e., up rounded length of two MRI pixels, 272 µm, 136 × 136 µm T2*Map MGE sequence, see Methods), a total 4500 µm length, and given number of bars. We used four designs namely *duo*, *trio*, *quad*, and *penta*, with 2-, 3-, 4-, 5-bars respectively (Supplementary Fig. 6a). Such that each MRI-barcode is qualitatively detectable and differentiable in MRI images. We used MRI-barcodes only for bundle identification in this work without any further encoding.

In vivo sample results from an implanted bundle with a quad-pattern barcode MRID-tag are shown in Figure 3b. Images with increasing TE show the spatial and temporal dynamics of induced contrast (Fig. 3b, the raw MRI images are shown in Supplementary Fig. 6b). Due to MRI soft-tissue contrast, anatomical regions have different and characteristic pixel intensity distributions (e.g., the corpus callosum has lower pixel intensities compared to cortical or thalamic areas). To accurately characterize the induced patterns of IONP islands, we detected the contrastive pixels as those with pixel intensities below -2 × z-score relative to the pixel intensity distribution of the anatomical structure (i.e., selected as the baseline), in which the pixel is located (Fig. 3b). IONP islands with lower IONP density become detectable at increasing TEs (Fig. 3b, IONP island 1). This demonstrates the capability to titrate the coated IONP density to better resolve the neighboring anatomical structures without shadowing them.

We quantified the contrast level of each pixel around MRID-tags with a sliding window (3 × 3 pixels, centered on each pixel) and created heatmap-like contrast maps around MRID-tags (see Methods, Fig. 3b, Supplementary Fig. 7). The sliding window strides by a single pixel, horizontally or vertically, to span the area around the MRID-tags. To quantify the contrast intensity at each pixel, we compared the mean T2*-relaxation within the sliding window to the mean relaxation of corresponding anatomical structure used as the baseline. The contrast heatmap reveals the induced 2D Gaussian-shaped contrasts, which are subsequently fit with Gaussian functions (Fig. 3b).

We reconstruct the implantation trajectory using best-fit Gaussian curve centers (i.e., estimated CoMs) in 3D MRI space (Fig. 3b). We compared the distances between estimated CoMs on the reconstructed trajectory to the actual distances between calculated CoMs on the design layout. We found a median absolute difference of 50.15 µm (**n** = 38 number of IONP island pairs) between the calculated and estimated inter-CoMs distances. (Fig. 3c). This result means that experimentally we can reconstruct the bundle trajectory with sub-pixel (i.e., 136 ×136 µm) and near-inter-channel separation (i.e., 50 µm) median error in 3D, which is sufficient to approximate the spatial locations of individual electrode channels.

We apply a point-set registration to find the best-fit of actual trajectory on the reconstructed trajectory (see Methods). Due to their ultra-flexibility, UFTE bundle trajectories in vivo are not necessarily straight lines, which sometimes follow slight arc- or S-shapes. We assume that the relative distances between CoM pairs and electrode channels remain constant during implantation with silk-fibroin coating. We position the actual trajectory that is defined by calculated CoMs in 3D MRI space near the reconstructed trajectory (see Methods, Fig. 3d, Supplementary. Fig. 8b). Point-set registration minimizes the total sum of distances between each calculated and estimated CoM pair (Fig. 3d) while keeping the dimensions of the actual trajectory based on the predefined constraints as described above (see Methods). The registration converges to a global minimum when more than 3 IONP islands are present in an MRID-tag. We observed multiple best-fits with a *duo-pattern* (i.e., 2 IONP islands in MRI-barcode).

For individual electrode localization, we map each electrode channel onto the best-fit trajectory in 3D MRI space. While MRI does not directly resolve individual electrodes, the relative positions of electrode contact sites to the CoM (i.e., ΔCoM_i__Ch_j_; *i*th IONP island, *j*th channel) are known from the design layout (Fig. 3aii). Electrode mapping on the best-fit trajectory can be done by these known relative distances (Fig. 3b, d), which yields the coordinates of electrode contact sites in 3D MRI space. We then warp the pixel coordinates of each electrode to the WHS using the transformation matrix from atlas registration to anatomically label each electrode channel (Fig. 3d, Supplementary Fig. 8).

For the bundle identification, we compute the similarities of the reconstructed MRI-barcode to all available barcodes in the design repertoire (i.e., four different barcodes in our case) using manhattan distance (see Methods). We found that reconstructed MRI-barcodes show a global mean 0.81 ± 0.09 SD similarity score (Fig. 3e, **n** = 17 MRI-barcodes). Using the similarity scores, we calculate the probability of the MRI-barcode belonging to any of the design barcodes using softmax algorithm (see Methods). Then, we assign the MRI-barcode to the highest probability barcode design (see Methods). We showed that 94.11% of the MRI-barcodes were correctly assigned (Fig. 3f, **n** = 1 false positive out of **n** = 17 assignments).

We compared the measured contrast intensities to the coated IONPs densities (**n** = 8 bundles). We define a region of interest (ROI) large enough and centered to cover the entire IONP island to measure and quantify the mean contrast intensities (see Methods). We set the IONP density as the number of IONP dots within the given ROI divided by the number of pixels of the ROI (number of IONP dots * pixel^−1^*)*. We observed a linear correlation between our IONP density to the induced contrast intensities (Fig. 3g). We found that IONP islands with a longitudinal length larger than two MRI pixels and at least 50 IONP dots per pixel can be reliably detected (7 T MRI), with a minimum mean observable IONP concentration of 0.0055 mM (see Methods, Supplementary Fig. 9). Four examples are given to show the contrast variance across animals, MRID-tags, and time in Supplementary Fig. 10.

### Validation of localization with electrophysiology and histology

We used electrophysiological landmarks to validate the accuracy of our MRID-tag electrode localization pipeline. Specific brain regions are known to exhibit spatially confined, characteristic oscillatory activity, which can serve as physiological ground truth for electrode positioning. The spatial extent of such oscillatory foci can be as small as approximately 100 µm² (in rat brain), providing a precise reference for validation. The dHPC in particular, which is known for its laminated organization and layer-specific theta and sharp wave–ripple (SWR) oscillations, was selected as the target region to validate MRID-based localization^6,25–27^. Therefore, we implanted 64-channel (50 µm inter-contact spacing) MRID-tagged ultra-flexible electrode (UFTE) bundles into the dHPC of the rat brain.

One of dHPC’s principal subdivisions, CA1, consists of four major laminae along the dorsoventral axis: stratum oriens (SO), stratum pyramidale (PyL), stratum radiatum (SR), stratum lacunosum-moleculare (SLM). Each layer exhibits characteristic activity patterns, particularly during network events such as SWRs and theta oscillations (Supplementary Fig. 11, Supplementary Video 1). SWRs arise from synchronous excitatory input of CA3 pyramidal neurons transmitted via the Schaffer collaterals^26^. These inputs target the apical dendrites of CA1 pyramidal neurons in stratum radiatum (SR), producing a large negative deflection in this layer as the functional signature of the input, along with high-frequency ripple oscillations (150–200 Hz) centered in the pyramidal layer (PyL)^6,26^. The sharp-wave component shows a polarity inversion between SR and SO, with maximal ripple power localized to PyL. Theta oscillations also display laminar-specific features, with theta power peaking near the hippocampal fissure (HF), and decreasing sharply toward the CA1–DG border^6,26^. As previously shown in the waking rat, theta waves exhibit a gradual phase reversal between CA1 SP and the SLM near hippocampal fissure^25^. Therefore, based on their electrophysiological features, we used PyL and HF as ground-truth landmarks to validate the accuracy of MRID-based localization.

**Fig. 4:**
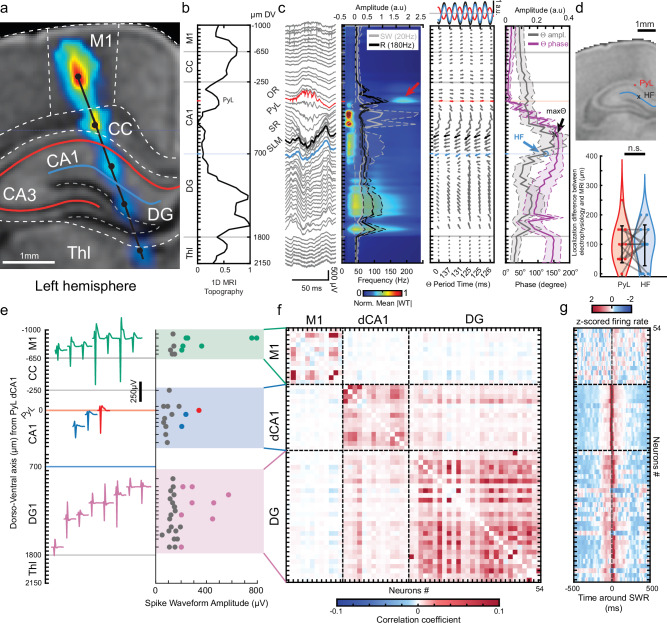
Accuracy of MRID-based electrode localization validated by electrophysiological landmarks. **a** Electrode locations (*black* lines) were mapped onto the Waxholm Space rat brain atlas^24^ (coronal view). The pyramidal layer (PyL-*red*) of dorsal CA1-CA2-CA3 is highlighted in red, while the granular layer of the dentate gyrus (DG) is marked by a *gray* dashed line. The white dashed line outlines the hippocampus (HP), corpus callosum (CC), motor cortex (M1), and brain boundaries. **b** Electrode alignment visualized using MRID-based topography. **c** The first column shows raw sharp-wave ripple (SWR, 0.1 Hz–7.5 kHz) activity along the electrode axis, with contact sites spaced at 50 µm. The *red* tick indicates the first electrode site corresponding to the pyramidal layer (PyL) in dorsal CA1, identified based on maximal ripple power. The *black* line shows the site where theta power reaches its maximum, and the *blue* line represents the position of the hippocampal fissure (HF). The second column presents the averaged SWR frequency decomposition (normalized mean |WT|–wavelet transformation; *n* = 463), showing ripple power along the DV axis at 184.14 Hz (*black* line, ±SD, dashed) and sharp-wave power at 20 Hz (*gray* line, ±SD, dashed). The red arrow marks the ripple power peak. The third column shows theta-filtered (4–10 Hz) LFP traces from three channels: PyL (*red*), maximum theta power (*black*), and the nearest HF channel (*blue*), with phase distributions aligned to the theta peaks from PyL. The fourth column displays theta power (*gray*) ±SD (dashed) and phase shifts (*purple*) relative to PyL. The black arrow indicates maximum theta, while the blue arrow marks HF. **d** The upper panel shows MRID-based localized PyL (*red*) and HF (*blue*) with the CA1-DG border (*light blue*). The lower panel compares MRI- and electrophysiologically-localized PyL and HF distances across three MRID bundles over a 5-month period (mean = 100 µm, SD = 62.67 µm for dHPC PyL; mean = 96.66 µm, SD = 69.35 µm for HF, *n* = 3 bundles from 2 rats). **e** Waveforms (>200 µV) are color-coded: M1 (green), dCA1 (blue and red [0 µm] in PyL), and DG (*purple*). **f** 2-ms binned cross-correlation matrix with delineated structural borders (dashed lines). **g** z-scored neuronal firing rates along the dorsoventral axis during SWRs.

Electrodes were, first, localized using MRID-tags (Fig. 4a, Supplementary Fig. 8) and anatomically labeled according to the Waxholm space (WHS) reference atlas^24^. We used the diffusion-weighted imaging (DWI) modality images of WHS to extract a 1D grayscale pixel intensity profile (pixel intensities are inverted solely for visualization purposes) along the registered electrode trajectory (Fig. 4b). We identified the electrode channel in PyL using this inverted DWI pixel intensity profile where PyL appears as a distinct peak in dCA1 due to its high cell density (Fig. 4b). To identify the electrode in HF, we used the border between CA1 and DG^6,25,35^ based on WHS anatomical labels.

Following the MRID-based identification of electrode channels located at the dCA1 PyL and HF, we used hippocampus-specific electrophysiological landmarks to identify corresponding contact sites. The highest ripple power was detected at 184.14 Hz in CA1 PyL (**n** = 463 SWRs; Fig. 4c, first column, red arrow), designating this site as the dCA1 PyL reference point (0 µm in Fig. 4c, second panel). We compute phase differences of theta cycles relative to the PyL as reference (**n** = 2303, peak at PyL: 0°, see Methods). A progressive phase shift is observed from 0° in the CA1 PyL toward dorsal channels (Fig. 4c). This shift is accompanied by an initial increase in theta amplitude, peaking near superficial CA1 before declining significantly beyond the HF. The maximum theta power channel is identified (Fig. 4c, black “X”), and theta power changes across channels are used to functionally locate the HF. A significant drop in theta power (z-test, *p* < 0.0015; CI [0.0570, 0.1689]; z = 3.1786) and a saturated phase shift mark the putative HF position (Fig. 4c, blue circle). The average phase difference between the CA1 PyL and the HF was 150.92° (**n** = 3 bundles), with individual bundle values as follows: Bundle 1: 167.77° ± 14.40° (**n** = 2306 cycles), Bundle 2: 146.81° ± 19.93° (**n** = 2303 cycles), Bundle 3: 138.20° ± 25.57° (**n** = 397 cycles).

To assess the accuracy of MRID-based localization, we measured the distances between MRID-based localized and electrophysiologically detected electrode channel locations in PyL and HF as the error (mean = 100 µm, SD = 62.67 µm for dHPC PyL; mean = 96.66 µm, SD = 69.35 µm for HF, **n** = 3 bundles from 2 rats, Fig. 4d). The inherent variations of statistical electrophysiological landmark detection and MRID-based electrode localization are shown in Supplementary Fig. 12a). For the CA1 PyL-identified as the channel exhibiting maximum ripple power, bundle 2 showed variation within ±50 µm across sessions, whereas bundles 1 and 3 showed no detectable variation (0 µm). In these bundles, the same channel was consistently identified as the pyramidal layer over extended recording periods (bundles 1 and 2 for 6 months; bundle 3 for 5 months). The underlying longitudinal localization differences across recording sessions for each bundle are shown in Supplementary Fig. 12b. A direct comparison of PyL and HF localization errors over the 5-month recording period using a two-sample t-test revealed no significant difference between the two regions (two-sample t-test: *t*(28) = 0.14, *p* = 0.89, pooled SD = 66.09 µm; Fig. 4d).

Furthermore, we investigated the inter- and intra-areal single- and multi-unit activity patterns to validate MRID-based anatomical labeling of individual electrode channels. The first column of Fig. 4e illustrates representative spike waveforms (*V*_peak-to-peak_ > 200 µV) aligned with their respective assigned structural labels (M1, CA1, DG) based on contact site locations. The PyL in CA1 and DG regions are distinguishable based on neuronal density, with a short gap before neurons reappear in the DG. The first neuron, located in the CA1 PyL as determined by electrophysiological analysis (i.e., maximum ripple power amplitude in CA1), is at the 0 µm position. The second column of Fig. 4e presents the mean amplitudes of spike waveforms recorded as high as 800 µV. Figure 4f displays the correlation matrix derived from binned (2 ms) spike data, which was subsequently analyzed using hierarchical clustering (Supplementary Figs. 13, 14). This analysis aimed to distinguish stronger intra-areal correlations from weak inter-areal correlations and to identify putative structural borders. Hierarchical clustering of neuronal activity correlations revealed three distinct functional clusters (see Methods).

We assessed the homogeneity of MRID-based anatomical labels (M1, CA1, DG) within each functional cluster. 50 of 54 neurons were correctly matched to their MRID-based labels, yielding an overall classification accuracy (see Methods) of 92.6% (M1: 10/13; CA1: 12/13; DG: 28/28). The few mismatches occurred at anatomical borders and remained within the error margin consistent with single-voxel MRI resolution. Notably, two M1-assigned neurons were mapped to the corpus callosum—an anatomically improbable location for single-neuron recordings—and one was assigned to CA1. To confirm robustness, we repeated the analysis in an independent dataset from a different animal and bundle with a more lateral/posterior trajectory (Supplementary Fig. 14). Here, 39 of 46 neurons were correctly matched, corresponding to an accuracy of 84.8% (Thal1: 6/7; Thal2: 8/12; CA1&CA3: 25/27). Within the thalamus, two functional clusters emerged, consistent with its multi-nuclear organization, although we did not attempt nucleus-level assignments. In the hippocampus, CA1 and CA3 neurons exhibited high correlation and clustered as a functional unit, in line with established dHPC circuit architecture.

PCA–k-means clustering of neuronal firing patterns during SWR events revealed distinct activation profiles across anatomically labeled regions (Fig. 4g, Supplementary Fig. 15; see Methods). Neurons recorded from MRID-identified CA1 and DG contact sites showed robust changes in firing rates during SWRs, while those from M1 contact sites did not. Specifically, CA1 neurons exhibited strong activation (z-scored firing rate: SWR = 1.3084 ± 0.6125 vs. Spontaneous = −0.1539 ± 0.0147; *t*(20) = 7.9164, *p* = 1.3 × 10⁻⁷), and DG neurons also showed significant modulation (SWR = 1.0067 ± 0.3569 vs. Spontaneous = −0.1162 ± 0.0196; *t*(20) = 10.4181, *p* = 1.58 × 10⁻⁹). In contrast, M1 neurons showed no significant change in activity during SWRs (SWR = 0.1244 ± 0.1229 vs. Spontaneous = 1.0067 ± 0.3569; *t*(20) = 2.5446, *p* = 0.0193, *α* = 0.01). Neurons recorded from CA1 and DG sites are functionally engaged during SWRs. In contrast, M1 neurons did not exhibit SWR-related activity. These findings further confirm the accuracy of MRID-based localization.

After five months of chronic experimentation, we performed electrolytic brain lesions to image the trajectory of electrodes in immunostaining images (see Methods). We imaged the coronal brain sections, where the electrolytic lesion is visible. For the comparison, we visualized the localized electrode channels on diffusion-weighted WHS coronal images where laminar structures of dHPC are visible. The MRID-localized electrodes exhibited trajectories that are well-aligned to the lesion sites (Supplementary Fig. 16).

### Chronic in vivo stability of MRID-tagged electrodes

**Fig. 5:**
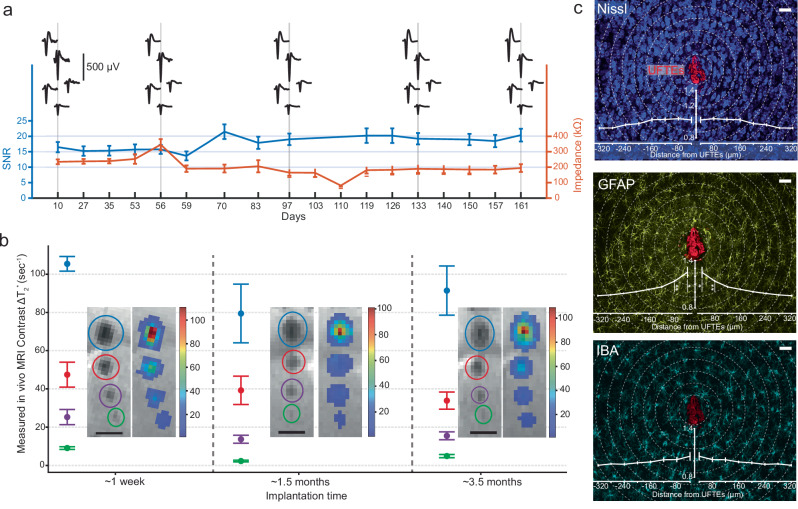
Chronic in vivo months-long electrophysiological- and MRI-contrast- stability of MRID-tagged ultra-flexible electrodes. **a** Chronic impedances from working electrode channels magnitude at 1 kHz <5 MΩ (*n* = 46 on day 10; *n* = 52 on day 161) are plotted, error-bars denote the standard error of the mean (s.e.m). Signal-to-noise ratio (SNR) values from single-units recorded each week are plotted, error-bars denote the s.e.m. 5 sample single-units are plotted chronologically on days 10, 56, 97, 133, and 161. **b** Chronic in vivo induced MRI contrasts over 3.5 months of a sample bundle. Mean and SD contrast intensities (5 most contrastive pixels) from each IONP island is plotted at 1-week, 1.5 months, and 3.5 months post-implantation (*blue*: IONP island #4, *red*: IONP island #3, *purple*: IONP island #2, *green*: IONP island #1), scale bars denote 1 mm. **c** Fluorescence immunohistology (animal #2) with Nissl, GFAP, and IBA-1 staining for neurons, astrocytes, and microglia, respectively. UFTEs are visible in red color (post-processing color assignment for illustrative purposes). From the border of UFTEs, 8 circular areas were defined with 40 µm separation, illustrated with circular dashed contours on the images. Scale bars denote 40 µm. The FI intensity changes are normalized with the mean of a large area >700 µm away from the border of the UFTEs and compared to the control condition (errorbars denote mean ± s.e.m, *n* = 13 brain slices, *n* = 4 bundles, Welch’s two-sample two-sided t-test, * and ** denote *p*-values, respectively, 0.0076 and 0.00016).

We carried out freely-moving electrophysiology recordings once a week (**n** = 3 bundles, **n** = 2 animals #1 and #3) over 5 months. In contrast, animal #2 was implanted chronically with four MRID-tagged UFTE bundles without external hardware (headstage, cranial screws, connector PCBs) solely for chronic MRI signal characterization and astrogliosis analysis. MRID-tagged UFTEs exhibit a robust and stable chronic recording performance. We measured the electrode-tissue impedances at 1 kHz before each recording session every week in vivo. Mean impedance magnitudes of 635.66 ± 84.16 kΩ (±s.e.m, working channels **n** = 119) and 234.80 ± 15.41 kΩ (± s.e.m, working channels **n** = 46) on days 13 and 10, respectively, from two animals, post-implantation. Mean impedance magnitudes of 122.71 ± 1.07 kΩ (± s.e.m, working channels **n** = 118) and 194.28 ± 25.34 kΩ (± s.e.m, working channels **n** = 52) on days 153 and 161, respectively, from two animals, post-implantation. The electrical electrode-tissue impedances drop and stabilize to a global mean of 144.60 kΩ over weeks. Mean ± s.e.m impedances from one animal are plotted chronically in Fig. 5a. More than 95% of contact sites remained functional throughout the five-month recording period (Supplementary Fig. 17).

We detected the chronically recorded and trackable single units (see Methods). Spikes were detected and sorted into clusters as described in our previous work^9,36^. Each cluster was identified as single- or multi-unit in a semi-automatic manner (see Methods). The clusters were separated from noise and neighboring clusters with expert judgment during the manual sorting. From the functional contacts (219 total out of 256, **n** = 3 MRID-tagged and **n** = 1 untagged UFTE bundle), we isolated 122 single units and 72 multi-units (see Methods for classification criteria). To evaluate long-term stability, we monitored electrode performance over several months (Supplementary Fig. 17). More than 50% of single- and multi- units were recorded over 5 months (**n** = 2 animals, **n** = 3 MRID-tagged UFTEs, Supplementary Fig. 17). Some selected single-unit mean waveforms (single-unit ID: 13, 23, 25, 31, and 42) were plotted over weeks in Fig. 5a. Electrodes exhibited a high-SNR stable recording over weeks. In Animal#1, we observed that SNR levels increased from 16.49 ± 1.47 (s.e.m) to 20.38 ± 2.08 (s.e.m) over a 23-week-long recording period, as shown in Fig. 5a. The increase in SNR is reversely correlated with the mean impedance values, such that as the impedances decrease, the SNR levels increase.

We investigated the induced MRI contrast from MRID-tags chronically (**n** = 5 bundles, **n** = 18 IONP islands, **n** = 3 rats), where MRID-tags remained detectable over long durations of chronic implantations. We measured and extracted the induced contrast heatmaps by each IONP island in MRID-tags (see Methods). Over 5 months long implantation duration, mean contrast intensities by MRID-tags showed 104.37%, 75.91%, and 99.56% changes compared to first MRI scans (first MRI: ~1 week after the implantation; for Animal #1, #2, and #3, respectively. Animal #2 implantation was for 3.5 months long; Supplementary Fig. 18). Two-sample dependent t-test is applied to the mean contrast intensities (**n** = 3 animals, **n** = 18 IONP islands, t-statistic = 1.663, *p*-value = 0.114 non-significant, df = 18, Shapiro-Wilk test for the normality of the data; statistic = 0.940 *p*-value = 0.260). Figure 5b shows the chronic MRI contrast induced by a single example MRID-tagged UFTE bundle. The contrast intensities from IONP islands, which are close to the insertion point, are most prone to intensity changes due to tissue healing from insertion.

We checked the immunohistology to analyze any adverse immune reaction to the MRID-tagged UFTEs. After 3.5 months of long implantation MRID-tagged UFTEs (**n** = 4 bundles, in Animal#2), the brain was dissected, prepared and immunostained to image the neurons (Nissl), microglia (IBA-1) and activated astrocytes (GFAP) densities (see Methods). From the borders of UFTE cross-sections, equidistance circular contours (40 µm thick) were defined (Fig. 5c). We statistically compared the mean normalized fluorescence intensity (FI) around UFTEs (<320 µm distance) to the control condition (>700 µm) at each defined circular contour (**n** = 13 brain slices). Neuronal density (Nissl) remained within a 10% change in the vicinity of UFTEs, with a mean increase of 2.63% ± 11.1% (s.e.m.) at the closest regions. These differences were not significant across all regions (Welch’s t-test). We measured an increase in activated astrocyte intensity within the 40 µm and 80 µm of UFTEs, with a mean percentage of 24.97% ± 19.76% (s.e.m) (Welch’s t-test, *p*-value = 0.0076) and with a mean percentage of 13.84% ± 4.70% (s.e.m) (Welch’s t-test, *p*-value = 0.00016). Activated microglia density also remained within 10% change at the surroundings of UFTEs, where, at the direct vicinity of UFTEs, there was a mean intensity increase by 5.89% ± 13.13% s.e.m (Welch’s t-test, non-significant for all regions).

In rat implantations, we assembled and prepared the electrodes in sterile environments without performing an additional sterilization step. However, for the MRID-tagged UFTE bundles’ further development and translation into NHP studies and clinics, we tested their sterilization and characterized them in vitro. Ethylene oxide (EtO) sterilization preserved the electrode impedances and silk-fibroin dissolution dynamics. Silk-fibroin was dissolved within 3 days in the protease enzyme when both sterilized and not-sterilized (i.e., control case). Moreover, there was no dissolution in phosphate-buffered saline in both sterilized and control cases (Supplementary Fig. 19). Electrode impedances were 49.94 ± 36.36 kΩ (mean ± SD) before sterilization and 54.65 ± 37.81 kΩ after sterilization, with the number of working channels remaining unchanged (**n** = 104 channels, Supplementary Fig. 19). The preliminary in vitro results exhibited that the functional and mechanical properties of MRID-tagged UFTE bundles remained preserved when sterilized with EtO.

## Discussion

Ultra-flexible electrodes are typically invisible in MRI, posing major challenges for accurate implantation, validation, and data interpretation in basic research and clinical applications. We developed MRID-tags for precision localization and identification of ultra-flexible electrodes.

We wafer-scale fabricate ultra-flexible electrodes with MRI-barcodes by coating IONPs using dot-matrix CCA technique. IONP dots do not form continuous films, which prevents uneven surface stress on individual ultra-flexible electrode fibers and preserves their intrinsic ultra-flexibility. This property is crucial for maintaining straight, linear bundle formation of fibers for implantation. The dot-matrix nanoparticle coating is a single-step technique without the need for intermediate stamps to transfer the nanoparticles onto the samples. We tested coating nanoparticles on different surfaces while minimally affecting the material properties and surface morphology. Using this technique, we can fabricate MRID-tags into any wafer-scale production ultra-flexible neural electrodes. Our technique is only limited by the available surface area (e.g., surface area of gold traces in this work), which determines the amount of coated nanoparticles. Some applications may require significantly larger quantities of coated nanoparticles (e.g., low field-strength MRI scanners). To overcome this, an intermediate thin layer of polyimide can be introduced (e.g., in a 3-layer polyimide structure), where IONPs are coated on gold traces and the intermediate polyimide layer effectively doubles the available surface area for coating.

We selected IONP to construct MRID-tags mainly due to their superparamagnetic properties. Our fabrication technique clusters contrast agent material (i.e., IONP) used, which makes the susceptibility-induced static dephasing effects dominant. Superparamagnetic IONPs exhibit strong susceptibility effects with small amounts^37^. Moreover, we encapsulate the contrast agent between polyimide layers, creating a large separation from water molecules in the tissue. This significantly reduces the inner- and outer-sphere effects between the contrast molecule and water proton. Therefore, the use of T1 contrast agent (e.g., Gd+3) is challenging with the current technique, which primarily relies on inner-sphere effects^37,38^.

We successfully imaged and detected MRID-tags chronically implanted in rat brains. We detected the induced contrast by MRID-tags spatially and temporally by taking multiple images at increasing TE. This allowed us to accurately identify the CoM of each IONP island in the MRI-barcodes. We showed that the detected CoMs align well with the calculated CoMs, allowing accurate reconstruction of implantation trajectories. We implemented a point-set registration algorithm to accurately determine the positions of individual electrode channels in 3D MRI space and to label them anatomically with the reference WHS atlas.

The peripheral implant apparatus, including cranial screws and electronics in the vicinity of the brain, can cause shadow artifacts in images. These large shadows might overlap with the contrast induced by the MRID-tags. To reduce such image artifacts, we used Polyether Ether Ketone (PEEK) and non-magnetic metals for the implant apparatus. MRID-tags were detectable everywhere in the brain except in the immediate vicinity of titanium cranial screws during such chronic implantation.

We observed a linear correlation between the measured MRI contrast and the density of coated IONPs. This linear correlation directly reflects the precision of our fabrication technique. Moreover, this linear correlation conforms with relaxivity equation of MRI contrast agents where the contrast agent concentration in medium linearly scales the induced MRI contrast^39^. Using this, we can measure the *observed concentration* of IONP in each IONP island. We named it as “observed” since the IONP dots are encapsulated in polyimide layers but not dispersed in the tissue. With this capability, specific contrast levels can be engineered depending on the applications, provided that conditions such as scanner type and implantation configuration are well defined.

We used the electrophysiological landmarks in the dHPC to validate our MRID-tagged electrode localization pipeline. The dCA1 PyL was identified based on peak ripple-band power during SWRs. The boundary with the DG, known as the HF, was defined by rapid decline in theta power distal to the SLM and a concomitant theta phase reversal, consistent with previous findings^6,25,26^. Using these electrophysiological landmarks as ground truth, we showed that MRID-based localization of contact sites closely aligned with well-defined neural structures.

Single-unit cross-correlations revealed functional groups where intra-area units exhibit high correlations in brain areas M1, CA1 and DG. SWR-associated neuronal activity showed clear modulation in CA1 and DG, while remaining unchanged in M1, which draws a distinct functional border between dHPC and cortex. We showed that the majority of anatomical labels from our MRI analysis matched the functional groups revealed by the single-unit firing rates.

MRID-tagged UFTEs exhibited stable impedances, high SNRs, reliable unit tracking, and consistent SNR over months. Impedances gradually decreased before stabilizing, likely due to post-implantation tissue recovery and integration of individual ultra-flexible fibers into the tissue. A high proportion of single- and multi-units remained trackable for over 5 month-long implantations. MRI contrast induced by MRID-tags remained stable over months, with only some changes in contrast intensity observed in superficial (i.e., dorsal) IONP islands, likely due to tissue healing from insertion. Stable MRI contrast also indicates that the polyimide fibers were not delaminated for 5.5 months of implantation in vivo.

We investigated the immune response in the brain. We observed only a mild increase in activated astrocyte density, and an unchanged density of activated microglia near the implant sites. Neuronal density remained unchanged near the implant sites without showing any loss of neurons. These results are consistent with our previous findings^9^, while our analysis in this work captures the variance across multiple bundles implanted  only in a single animal. A further group-level study with multiple animals is necessary to make statistical assessment of biocompatibility.

Ultra-flexible electrodes similar to UFTEs have been previously detected only in post-mortem tissues^8^. Radiopaque markers could be used to enhance contrast in CT^19^. However, the amount of metal (e.g., gold) used in radiopaque markers in DBS probes is orders of magnitude larger than the footprint of ultra-flexible probes (e.g., UFTEs) can accommodate. In this study, we demonstrate that ultra-flexible electrodes can be accurately and reliably identified and localized using MRID-tags in MRI without changing the footprints of electrodes. MRID-tagged ultra-flexible electrodes could also be potentially used during fMRI. However, the susceptibility-induced artifacts from MRID-tags might interfere with the quality of fMRI signal within the proximity of electrodes, depending on the particular implantation site, and fMRI imaging sequences used. These need to be tested in future studies.

The ability to reliably localize ultra-flexible, minimally invasive neural implants has broad translational potential. In research, the safe and stable long-term use of MRID-tagged ultra-flexible electrodes can enhance nonhuman primate studies by enabling precise anatomical localization, thereby bridging basic neuroscience and human clinical applications. In clinical practice, this technology could replace large, millimeter-scale electrodes with miniaturized, MRI-visible ones, improving both the targeting and monitoring of electrophysiological signals in deep-brain stimulation for movement disorders and in intracranial recordings for epilepsy diagnostics and therapy. Beyond these established applications, precise localization of MRID-tagged electrodes may further accelerate the development of next-generation, high-density brain–machine interfaces.

## Methods

All experimental and surgical procedures involving animals were approved by the local veterinary authorities of Canton Zurich, Switzerland, and were carried out in accordance with the guidelines published in the European Communities Council Directives 2010/63/EU.

### Microfabrication of the ultra-flexible electrodes and MRID-tags

We fabricated the UFTEs following the same layer-by-layer lithography-based recipe as in our previous work^9^. We coated a bare silicon wafer on its polished side with 1 µm thick PI2610 polyimide (PI) and cured it in the programmable oven (CLO-2AH-S, Koyo ThermoSystems). On the base polyimide layer, we deposited and patterned 10 nm titanium (Ti) and 150 nm gold (Au) to construct the electrode recording sites, wires, and soldering pads.

For the dot-matrix nanoparticle coating, we lithographically patterned a thin photoresist layer (AZ1505, Microchemicals GmbH) on the sample. After the development, dot-matrix traps exposing Au surfaces underneath were achieved. Development profiles and alignment of dots on the gold traces were checked under the optical microscope (Nikon Eclipse L200D). The sample is dipped into 1:99 diluted gold etchant (KI/I_2_, FIRST Micro- and Nanotechnology Center, ETH Zurich) in dH_2_O and agitated for 15 s to roughen the exposed Au surfaces in dot-matrix traps. The sample is rinsed and dried.

The sample is placed on the wafer-chuck attached to the linear motorized stage of the custom nanoparticle coating setup (Supplementary Fig. 2a). Electrode wires, which are to be coated with IONP dots, are positioned parallel to the movement of the linear stage. A glass microscope slide is placed above the sample with 500 µm separation. A micromanipulator is used for precise positioning of the glass slide guided with images from a horizontal microscope. Using a micropipette, 2 µl colloidal suspension of 0.25 mg/ml (25 nm average particle size) iron(II,III)-oxide nanoparticles (Sigma-Aldrich 900042) is injected between the sample and the glass slide where the electrode wires are. The meniscus that is formed by colloidal suspension is imaged with a horizontal microscope to measure the contact angle. The sample is moved in the direction where the meniscus contact line is, at a constant velocity of 5 µm/s until the meniscus is no longer on the dot-matrix. The remaining colloidal suspension is removed with a micropipette. The operation is repeated for each electrode bundle.

AZ100 Remover (1-aminopropan-2-ol and dipropylene glycol methyl ether, Microchemicals GmbH) in a 5-in.-wide beaker is heated up to 80 °C in a water bath on a hotplate in a controlled manner. The sample is placed in the 80 °C AZ100 Remover for 10 min. A gentle mechanical agitation is applied to the sample using a Pasteur pipette every 5 min. The process is repeated with a clean 80 °C AZ100 Remover bath for another 10 min. The sample is rinsed consecutively in isopropanol and running dH_2_O baths, and afterward dried with N_2_ air gun. Following the removal of photoresist and excess IONP, the coating results are validated in optical (Nikon Eclipse L200D) and scanning electron microscopy (Zeiss Gemini ULTRA 55).

We coated another 1 µm-thick polyimide layer on the sample and cured it. We etched the polyimide in between individual electrode fibers and at the outer borders^9^ to construct physically independent electrode fibers. We coated the sample with a 2.5 µm thick sacrificial parylene-C layer with chemical vapor deposition (PDS 2010 Labcoater 2; Specialty Coating Systems Inc.)^9^. We etched the parylene-C and top polyimide layers on active recording sites to expose the Au surface underneath. We checked the quality of etching under an optical microscope (Nikon Eclipse L200D).

We roughened the Au surface on the recording sites with gold etchant (KI/I2, FIRST Micro- and Nanotechnology Center, ETH Zurich) chemically. We spin-coated (three times at 650 RPM, ~450 nm final layer thickness) PEDOT:PSS (Clevios PH 1000, Heraeus Epurio; ethylene glycol, Sigma Aldrich; dodecyl benzene sulfonic acid, Sigma-Aldrich; and 3-Glycidyloxypropyl trimethoxysilane, Sigma Aldrich) on the sample and baked it at 140 °C for 1 h^9,40^. We peeled off the parylene-c sacrificial layer, leaving PEDOT:PSS only on the contact sites, which reduces the electrode-tissue impedance^9^.

### Assembly of the UFTEs

We soldered 70-pin mezzanine (AXF5A7012A, AXF6A7012A Panasonic) female and male type connectors on the custom design flexible and rigid PCBs, respectively. Two 36-pin (A79024, Omnetics) female Omnetics connectors are soldered from both sides at the other end of the rigid PCB (Supplementary Fig. 20a). We peeled the 64-channel MRID-tagged UFTE bundles off the silicon wafer while the individual fibers were immersed in silk-fibroin solution. We, then, soldered the UFTEs to the flexible PCBs (Supplementary Fig. 20a) using a standard solder and soldering iron. We applied a medical-grade epoxy (EP40MED, MasterBond Inc.) on the soldering area to mechanically secure the soldering between electrodes and PCBs. We cured the epoxy at room temperature for 48–72 h. Flexible PCB is plugged to rigid PCB with mezzanine connectors, and rigid-PCB is plugged to the 64-channel (RHD 64-Channel Recording Headstage, Intan Technologies) recording headstage. In vitro impedances at 1 kHz were measured in saline (Ringer’s solution, B.Braun) with a two-electrode configuration where an Ag|AgCl wire was used as a reference/counter electrode using the Intan RHD recording system (Supplementary Fig. 19c, 20a). After soldering and quality check of UFTEs, we assembled them with tungsten insertion shuttles as described in ref. ^9^.

### Modular *titaniumHelmet* and 3D-printed housing

The *titaniumHelmet* from the previous work is adapted in this work^9^. The titaniumHelmet spans a maximum of 23.95 mm in the anterior-posterior axis and 11.30 mm laterally as stated previously^9^. We replaced the base part (Supplementary Fig. 20c) with a PEEK-made base to prevent the MRI image artefacts induced by the base while keeping the outer shell as grade 5 Titanium (Ti-6Al-4V). The PEEK base has two anterior screw holes (1 mm diameter) and one posterior screw hole (1 mm diameter). The PEEK base is permanently screwed onto the skull with 0.9 mm cranial titanium screws (length 3 mm, M-5100.03, Medartis AG, Switzerland) and cemented. Five lateral screw holes with threads (M1.2) on the base are used for assembling the outer shell of titaniumHelmet to the base.

We 3-D printed a custom design inner housing to hold the connectors during freely-moving recording (Supplementary Fig. 20c). The 3-D printed inner housing has three parts assembled inside the titaniumHelmet (Supplementary Fig. 20c). These pieces have precise slots to position the connector PCBs in. Flexible-PCBs folded twice during the assembly of the helmet, and rigid PCBs are positioned in their dedicated slots, where Omnetics connectors face up (dorsal) (Supplementary Fig. 20c). This inner housing tightly holds the PCBs, ensuring a reliable plugging of Omnetics connectors to the Intan headstages before the freely-moving recordings, and maintains the connection throughout the recording. The total weight of the assembly is 35.3 g.

### Surgical procedures

We used female Long Evans rats (**n** = 7 rats, 24–80 weeks of age, ~300 g of weight at the time of the surgery, Janvier Labs and Charles River Laboratories). Female rats were chosen due to a lower chance of cage-mate fighting and to prevent single housing. The rats were housed in groups in standard IVC cages (Allentown) and had ad libitum access to food and water. The cages were kept at 23 °C room temperature, 52% room humidity, and 58% cage humidity, and on an inverted light cycle (12 h dark/12 h light).

All surgical steps are performed aseptically. We anaesthetized the rats with inhalation of isoflurane (Attane, Piramal Pharma Ltd) mixed with oxygen. We monitored the respiration rate, heart rate, and blood oxygenation levels of the rat throughout the entire duration when the animal was under anesthesia (Model 1030, Small Animal Instruments Inc.). We adjusted the level of anesthetics and analgesics during the surgery based on the vital signs readout (respiration rate, heart rate, and blood oxygenation). We monitored the body temperature of the animal with a rectal probe which is also connected to a closed-loop heating pad that is placed underneath the animal. Analgesia was provided as pre-operative Tramadol (p.o., Tramadol chlorhydrate, Sandoz) and Meloxicam (s.c. 2 mg/kg, Metacam, Boehringer Ingelheim) during surgery. A mixture of Ringer’s solution and glucose (Aequifusine, B. Braun) was injected subcutaneously (s.c.) in the torso at fixed intervals during the surgery to maintain the water, electrolyte, and sugar levels of the animal (up to 5 ml s.c. for the initial injection, and every 4 h up to 2 ml s.c. during the surgery). We trimmed and shaved the hair on the rat’s head and cleaned the scalp with Betadine (Mundipharma Deutschland GmbH) and protected the eyes with an eye ointment. A mixture of Bupivacaine (Bupivacaine Sintetica, Sintetica), Lidocaine used for local anesthetic (maximally 7 mg/kg bodyweight is injected under the skin at the site of the surgery), and NaCl solution is subcutaneously injected at the scalp at multiple locations. We fixed the rat’s head at the stereotaxic frame with earbars and incised the skin with a single cut in the anterior-posterior axis. We cleared the connective tissue to expose the skull surface, especially the Bregma and Lambda of the skull. We iteratively ensured the parallelity and levelness of the skull with the ground surface. We identified and drilled the craniotomy locations; three craniotomy holes were drilled for fixating the base with a 0.9 mm drill bit (Medartis), two lateral craniotomy holes that were used to ensure a strong cementing base were drilled with a 1.2 mm drill bit (Medartis), and two holes for the electrode implantation craniotomies were drilled with 0.9 mm dental drill bits. We fixed the PEEK base with screws and dental cements. MRID-tagged 64-channel UFTE bundles (*n* = 3) were through the dorsal hippocampus (dHPC) of rats (Animal 1: {AP = −3.24 mm, ML = 2.2 mm}, Animal 3: {Bundles coordinates left- and right- hemisphere; AP_Left_ = −3.0 mm ML_Left_ = 2.2 mm AP_Right_ = −2.7 mm, ML_Right_ = −2.45 mm}) same way as described in ref. ^9^. The ribbon parts of UFTEs were covered with dental cement on the skull. We positioned the lower end of the flexible PCBs on the skull and cemented them to their lateral anchor extensions (Supplementary Fig. 20b). Once we implanted all the MRID-tagged UFTEs and secured their PCBs with cement, we assembled the 3-D printed inner housing together with TitaniumHelmet. We cleaned the wound and sutured gaps in anterior and posterior parts of the scalp. We stopped the isoflurane flow and transferred the rat into a clean, warm cage with wet food pellets, bedding and nesting material. We closely observed the rat until it fully woke up. The rat recovered single housed with tramadol (Tramadol chlorhydrate, Sandoz), drinking water (0.25 mg/ml) readily available in the cage and 2 mg/kg per body weight Meloxicam administered twice a day.

### Imaging the rat brain in vivo in MRI

We scanned and imaged the rat’s brain and MRID-tags in MRI 10–14 days after the implantation and monthly throughout the experimentation. Before the MRI scanning, we anaesthetized the rats with inhalation of isoflurane (Attane, Piramal Pharma Ltd) mixed with oxygen (4% in 1l*t*/min oxygen flow for the induction). We disassembled the outer titanium shells and 3-D printed pieces, only leaving the PEEK base and connector PCBs on the animal’s head. We positioned the rat on the warm MRI cradle in head-prone position, fixated with earbars. We monitored the body temperature, respiration rate, heart rate and blood oxygenation levels of the rat while the animal was under anesthesia (Model 1030, Small Animal Instruments Inc.), and we adjusted the isoflurane concentration according to vital signs. The 30 mm diameter ring surface receives only coil (^1^H planar receive-only 30 mm surface coil, Bruker, Supplementary Fig. 20c) is positioned on the animal’s head. The flexible connector PCBs are guided through the inner loop of the coil and pulled towards as posterior as possible further away from the imaging region of interest (Supplementary Fig. 20c). The PCBs are gently fixed via a 3 M tape at the posterior. We positioned the head of the rat at the isocenter of the MRI (7 Tesla PharmaScan 70/16, Bruker) bore. We matched the impedance of the RF transmission coil per scanning session. We took a localizer scan and ran automatic adjustment settings, e.g., shimming. After localizing the subject, we took three TurboRARE spin-echo (SE) scans (TE = 33 ms, TR = 2500 ms, RARE factor = 8, echo spacing = 11, excitation angle = 90, refocusing angle = 180) in axial, coronal, sagittal slicing orientations (slice thicknesses: 800 µm, 450 µm, 550 µm respectively) covering the entire brain (field-of-views: 35 mm × 35 mm, 18.4 mm × 12 mm, 35 mm × 35 mm and image dimensions: 256 × 256, 180 × 120, 256 × 256, respectively). We mapped the B0 field homogeneity in the bore with B0MAP sequence (2 echo images TE_0_ = 1.64 ms and TE_1_ = 5.45 ms, flip angle = 30, TR = 20 ms, image size: 64 × 64 × 64, FOV: 45 mm × 45 mm × 45 mm). We took T2*Map multiple gradient echo (MGE) images (Echo images = 8, TE_0_ = 4.09 ms, echo-spacing = 4 ms, TR = 800 ms, flip angle = 50, FOV: 35 mm × 35 mm, image size: 256 × 256) in coronal and sagittal slice orientation where slices (slice thickness = 800 µm) are aligned to MRID-tags based on whole brain SE images. Using the same slice orientation and FOV from T2*Map MGE images, we took FLASH (TE = 6 ms, TR = 200 ms, flip angle = 30, image size: 512 × 512) and TurboRARE SE (TE = 33 ms, TR = 2500 ms, RARE factor = 8, echo spacing = 11, excitation angle = 90, refocusing angle = 180) images.

### Brain registration SAMRI

We registered the MRI images from each imaging session to the reference WHS atlas using the Small Animal Magnetic Resonance Imaging toolbox (SAMRI)^41,42^. We first converted all the images to NIFTI format from Bruker format. We bias-corrected the coronal whole-brain SE images before the registration (Supplementary Fig. 8a)^43^. After bias correction, we registered the coronal whole brain SE images to WHS T2* images in rigid, affine and elastic registrations consecutively^41,42,44^ (Supplementary Fig. 8a). After the registration, we checked the quality of the registration by looking at the outer borders, corpus callosum and olfactory bulb on the warped coronal whole brain SE images overlapped with WHS anatomical segmentation. We applied the resulting transformation^44^ to the other images within the session to have all images in the WHS reference space.

### COMSOL simulations

We designed a custom microstrip patch antenna to simulate radio-frequency (RF) excitation waves transmitted by MRI (~297 MHz Larmor Frequency at 7 T). We set the total RF power delivered by the antenna to the actual delivered value in the 7 T Bruker scanner. We used a spherical volume large enough as the perfectly matching layer. We used the human head phantom model (the relative permittivity *ε*_*r*_ = 58.13) provided by COMSOL^45^. We closely positioned the head phantom to the patch antenna (Supplementary Fig. 4a). We matched and tuned the antenna when the phantom head was closely positioned, where the S_11_-curve (Supplementary Fig. 4b) shows that the antenna is matched and tuned to 297 MHz.

First, we simulated the heating induced by RF waves in the phantom head without any implantation as the control. We modeled the implanted electrodes as one-dimensional strips (6 mm long) with polyimide and gold (from COMSOL material library) as the main materials. We positioned the electrodes at the center of the phantom head also well-aligned to the center of the patch antenna. We simulated the induced heating with implanted electrodes afterward (Supplementary Fig. 4c-d).

### IONP relaxivity

The induced contrast is linearly correlated with the concentration of IONPs ([CM]) in a medium defined by the relaxivity equation (Eq. 1)^39^. *T*_*i*_ denotes the observed relaxation time constant, *T*_*i*_^0^ denotes the relaxation time constant of the medium without any contrast in the medium ([CM] = 0), and *r*_*i*_ denotes the relaxivity constant of the contrast agent (in mM^−1^.s^−1^, where *i:* 1, 2, 2* i.e., MRI modalities).

1 $$\frac{1}{{T}_{i}}=\frac{1}{{T}_{i}^{0}}+{r}_{i}\cdot [{CM}]$$

We prepared phantoms at varying concentrations (i.e., 0.02 mM, 0.04 mM, 0.08 mM, 0.12 mM) of IONP (231.53 g·mol^−1^, Sigma Aldrich 900042) in dH_2_O. We imaged (four coronal imaging slices, pixel size = 136 × 136 µm, slice thickness = 800 µm, averages = 3) each IONP phantom separately in MRI with T2*map-MGE sequence (TE0 = 4.00 ms, TE_spacing = 4.09 ms, number of echo images = 32) as described previously. At each imaging session, we positioned a dH_2_O phantom next to IONP phantom as the control and imaged it in the same manner.

To obtain T2* relaxation times, we manually segmented images in ITK-SNAP^46^ into “control” and “IONP” region of interests (ROIs). Relaxation time is defined as the time point when pixel intensity drops to 37%^47^. From each ROI, we collected and calculated the mean pixel intensity values at each echo time. We fitted exponential decay curves (Eq. 2) to the decreasing mean pixel intensity values (*n* = 4 mean intensities from *n *= 4 imaging slices) to extract the relaxation curves (*S*_0_: initial signal intensity, T2*: relaxation time, *t*: echo time, *A*: bias, *S*: signal at echo time = *t*). “T2*” value (Eq. 2) in best-fit exponential decay gives the relaxation time of the ROI. We fit a line to the measured T2* values at increasing concentrations, where the slope of the line gives the relaxivity constant (i.e., *r*_2*_) of the IONP (Supplementary Fig. 9).

2 $$S={S}_{0}\,{e}^{\frac{-t}{T2*}}+A$$

### IONP-coated UFTE bundle phantom tests

We inserted four UFTE bundles (**n** = 2 IONP coated, **n** = 2 standard) into phantom (2% agarose in dH2O prepared in 50 ml falcon tube, Sigma Aldrich A5304-100G). We imaged the phantom in 7 T MRI (7 Tesla PharmaScan 70/16, Bruker) as described previously with the T2 TurboRARE imaging sequence. We imaged the phantom in 3 T clinical MRI (Philips Ingenia Elition X 3 T) with T2-weighted spin-echo imaging sequence (pixel size = 185 × 185 µm, slice thickness = 1.0 mm, echo time = 100 ms, repetition time = 3606 ms, number of averages = 4).

### MRID analysis

On unregistered raw T2*Map MGE images, we manually segmented the MRID-tags and surrounding anatomy using ITK-SNAP^46^ at the echo image where the patterns are the largest. We assigned a separate label for each IONP island (Supplementary Fig. 8b). We excluded the areas where strong magnetic field inhomogeneities (e.g., due to air gaps) are present from the segmentation. Furthermore, we segmented the same images entirely based on anatomy, ignoring the MRID patterns, such that each pixel in an MRID pattern has an assigned anatomical label. After manual segmentation, we have two segmentation images, namely, MRID-pattern segmentation and anatomical segmentation.

IONPs in MRID-tags are encapsulated in polyimide layers; therefore, we cannot measure the local concentration directly, but instead we have an observed concentration based on Eq. 1. The observed concentration is correlated with the number of dots (i.e., in the corresponding dot-matrix) in each IONP island.

Based on the anatomical segmentation, each pixel is assigned to an anatomical structure. We first calculate the T2* relaxation time of the given anatomical structure where the pixel is located (Methods, Eq. 2) as the baseline, i.e., *T*_2*_^0^. We, then, centered a 3 × 3 pixels window at the given pixel on segmented MRID patterns, collecting pixel intensity values (*n* = 9, pixel intensities) at each echo image (Supplementary Fig. 7). We calculated the T2* relaxation time within the window—the relaxation time *T*_2*_^j,k^ at pixel [j,k]—while fixing the *S*_0_ and *A* to the values from the baseline anatomical structure’s best curve fit (see Methods, Eq. 2). We made this assumption since IONPs are encapsulated into a tiny footprint, thus, *S*_0_ and *A* values should be identical to the baseline anatomical structure values.

The measured contrast level *c*_2*_^j,k^ at pixel [*j*,*k*] can be calculated with Eq. 3 (Supplementary Fig. 7). We created the heatmap contrast intensity images using *c*_2*_^j,k^ values (Supplementary Fig. 7). We transferred these images into 3D MRI space using the affine transformation from raw images (Supplementary Fig. 8b).

3 $${c}_{i}^{\,\,j,k}=\frac{1}{{T}_{i}^{j,k}}-\frac{1}{{T}_{i}^{0}}i:\{1,2,2*\}$$

We fitted a 2-dimensional Gaussian curve on each IONP island contrast heatmap in each imaging orientation (e.g., coronal, sagittal etc.). We extracted the 3-dimensional (in medio-lateral, dorso-ventral, and anterio–posterior axes) pixel indices of fitted Gaussian centers for each IONP island. We picked the pixel index in the dorso-ventral axis from the slice orientation (coronal or sagittal) at which the contrast intensity is higher.

### Bundle trajectory point-set registration

The loss function, to be minimized, of point-set registration is the summation of all Euclidean distances between calculated and estimated CoM pairs (Eq. 4, *k*: {1…*M*}, *M* is the number of IONP islands). UFTE bundle trajectories in vivo are not necessarily straight lines, which sometimes follow slight arc- or S-shapes. We assume that the relative distances between CoM pairs and electrode channels remain constant during implantation. Under these assumptions, we can formulate the bundle trajectory in the spherical coordinate system. We can define each calculated CoM as a node connected to other nodes with a fixed distance *r*_k_ but variable inclination and azimuth angles (*θ*_*k*_ and *φ*_*k*_). The first CoM_1_ is only defined in the Cartesian coordinate system, where it is initially positioned to the arbitrary *p*_*1intial*_ = (*x*_*1intial*_, *y*_*1intial*_, *z*_*1intial*_). The relation between each consecutive calculated CoM pair in the Cartesian coordinate system is given in Eq. 5. Then, our point-set registration task simply becomes a search on *p*_*1*_, *θ*_*k*_, and *φ*_*k*_ to minimize the loss function *L* (Eq. 6) (shown in Fig. 3c).

4 $$L={\sum }_{k}{d}_{k}$$

5 $${{CoM}}_{k+1}={{CoM}}_{k}+({r}_{k}\sin {\theta }_{k}\cos {\varphi }_{k},{r}_{k}\sin {\theta }_{k}\sin {\varphi }_{k},{r}_{k}\cos {\theta }_{k})$$

where $${{CoM}}_{1}={p}_{1}=({x}_{1},{y}_{1},{z}_{1})$$

6 $${{\arg }}_{{p}_{1},{\theta }_{k},{\varphi }_{k}}\min {\sum }_{k}{d}_{k}$$

To find the set of [*p*_*1*_, *θ*_*k*_, *φ*_*k*_] minimizing the loss function, we used Broyden–Fletcher–Goldfarb–Shanno iterative optimization method^48–51^. The first point is initially positioned, *p*_*1intial*_ near the first measured CoM with a randomly sampled slight shift to allow the search algorithm to converge fast.

### MRI contrast versus IONP dot coating density

To compare the IONP density to the induced contrast intensity, we centered a circular window (*r* = IONP island length/2) at the Gaussian centers. We calculated the relaxation times and contrast intensities from the circular window using Eqs. 1–3. We calculated the IONP density as the number of dots in the given IONP island divided by the number pixels in the circular window (number of IONP dots * pixel^-1^). For the fully coated bundle, we fitted a line along the trajectory of the bundle. Along this line, we slid regions of interest at different sizes and calculated the contrast intensity for each ROI. For each ROI, we calculated the number of dots in that region using the design layout.

### MRI-barcodes

We generated a fixed-length vertical (array length = 4500 i.e., 1 sample == 1 µm) white (all ones) rectangle template (4500 × 1000, an arbitrary width = 1000 solely for visualization) for MRID barcode reconstruction. At the bottom, we colored a horizontal black bar (zeros) in the empty rectangle template with a height of 2× sigma of the most ventral MRID pattern gaussian fit in the dorso-ventral axis. From the center of the black bar, we moved up with the distance between the most and the second ventral gaussian centers. We colored another horizontal black bar centered around that point with the height of 2× sigma of the second most ventral MRID pattern. We repeated this process for all the MRID patterns, filling up the barcode template upwards (i.e., towards dorsal). We generated the reference barcodes (e.g., duo, trio, quad, and penta) from design layouts in a similar manner, using the CoMs as the bar centers and length of the IONP island coating area as the bar heights (total barcode length is 4500, 1 µm == 1 sample).

To calculate the similarity score, we computed the manhattan distance between the reconstructed MRI-barcode (4500 × 1 array) to each reference barcode (4500 × 1 array) (k: duo, trio, quad, and penta reference barcodes, Eq. 7). We normalized the similarity score with the total barcode length 4500 yielding a similarity score between 1.00 for perfect match and 0.00 for no match. We used a softmax algorithm to calculate the probability of an MRI-barcode belonging to each category in reference barcodes (Eq. 8). We computed the similarity scores of each reference barcode to other reference barcodes and set the sigma (i.e., temperature) parameter of the softmax algorithm to standard deviation of reference barcodes similarity scores.

7 $${s}_{k}=\frac{{\sum }_{i=1}^{4500}\left|{x}_{i}-{x}_{i}^{k}\right|}{4500}$$

8 $$p=\frac{{e}^{\frac{{s}_{k}}{2{\sigma }^{2}}}}{{\sum }_{k}{e}^{\frac{{s}_{k}}{2{\sigma }^{2}}}}$$

### In vivo recordings from rats

The rats were already familiar with the testing environment. We used one of three environments: (i) a 50 × 50 × 50 cm Plexiglas cage covered with copper mesh on its sides and bottom (with an open top) and a floor lined with bedding that was changed after each recording session, (ii) a square arena measuring 1.5 × 1.5 m covered with green PVC, or (iii) an 80 cm-diameter circular open-field desk, also covered with green PVC.

Recordings began within 6 days after surgery, at least twice a month. In all environments, a glass Petri dish containing a single drop of concentrated milk (Kondensmilch, Coop Switzerland) was a positive reward after connecting the recording system to the rat.

For data acquisition, we used an Intan Technologies 32-channel head stage (part# C3314) with RHX Data Acquisition Software (https://www.intantech.com) to record broadband data at a 20,000 Hz/channel sampling rate and 16-bit resolution. A high-pass filter with a 0.1 Hz cutoff frequency was applied at the hardware level to eliminate the DC component of the signal.

### Spike sorting and SNR calculation

Spike sorting for selected recording sessions was performed using JRCLUST 4.0.0^36^. First, the pipeline filtered the raw data with a 4th-order bandpass filter, with cutoff frequencies set at 300 and 5000 Hz. Next, common average referencing was applied by computing the median across all intact channels and subtracting this median from each channel’s filtered trace, minimizing artifacts from instrumentation or strong muscle movements. Spikes were then detected in the filtered and software-referenced traces as described by Quian-Quiroga et al. (qqFactor = 5, only negative peaks detected). Events detected within a 60 μm spatial and 0.25 ms temporal vicinity (“evtDetectRad,” “evtMergeRad,” and “refracInt”) were merged into a single spiking event to prevent duplicate detections across multiple recording contacts.

To reduce the dimensionality of the detected spike waveforms, JRCLUST applied principal component analysis (PCA) with three features per recording contact (“nPCsPerSite”). Spikes were then clustered automatically using the Density Peak clustering algorithm^52^, with logarithmic rho and delta cutoffs set at −2.5 and 0.6, respectively (“log10RhoCut” and “log10DeltaCut”). Manual curation was performed following automatic clustering to eliminate noise clusters and finalize spike cluster identities. A unit was classified as a single unit if fewer than 2% of inter-spike intervals (ISIs) were shorter than 2 ms, the waveform shape and amplitude remained stable throughout the recording period, and the spike cluster exhibited clear separation from noise in feature space. Cluster quality was further assessed using isolation metrics, with an Isolation Distance >20 or an L-ratio <0.05 considered acceptable for single-unit classification^53^.

Chronic single-unit SNR levels were calculated using single-units from concatenated spike-sorted data. SNR of a session was defined by the mean ratio of the peak-to-trough amplitudes of all single-units within that session to the session *V*_*rms*_.

### Local field potential data processing

To extract and analyze local field potentials (LFPs), we implemented an offline data processing pipeline, illustrated schematically in Supplementary Fig. 21, which shows the workflow for pyramidal layer (PyL) and hippocampal fissure (HF) localization.

To extract and analyze LFPs, we applied a low-pass filtering and down-sampling procedure to the raw electrophysiological data recorded at 20 kHz. The raw data were stored in binary.dat files in a 16-bit integer format, containing signals from all recording channels. The data were processed using a custom MATLAB script with several steps. First, the recording file was memory-mapped for efficient access, and a binary output file (.lfp) was created to store the processed signals. To isolate the LFP component, we applied a 600th-order finite impulse response (FIR) low-pass filter with a 245 Hz passband frequency and a 250 Hz stopband frequency at a 20 kHz sampling rate. The filter was designed using MATLAB’s designfilt function and applied bidirectionally using filtfilt to minimize phase distortion. Each recorded channel was extracted and converted to microvolts using a scaling factor of 0.195 µV/bit, and the low-pass filter was applied sequentially to each channel. After filtering, the data were downsampled by a factor of 10, reducing the sampling rate from 20 kHz to 2 kHz, which was appropriate for LFP analysis while maintaining computational efficiency. Finally, the filtered and down-sampled signals were stored in a.lfp binary file in the same 16-bit integer format for further analysis.

### Sharp-wave ripple detection, alignment, and frequency decomposition of local field potentials

LFP signals were recorded from eight selected channels spanning CA1 strata: stratum radiatum (SWR-negative deflection), pyramidal layer (ripple detection), and stratum oriens (SWR-positive deflection). Signals were downsampled to 2 kHz for LFP analysis.

SWRs were detected using a convolutional neural network (CNN)-based SWR detection method^54^. For manual curation, a MATLAB-based graphical interface was developed, enabling interactive browsing of multichannel LFP traces, bandpass-filtered signals, ripple power, and wavelet-transformed data across hippocampal layers. SWRs could be manually inspected and annotated by specifying event onset and offset points. To standardize SWR alignment, we converted detected SWR intervals to a central alignment time point. This involved a two-step procedure: (1) Identifying the peak of SWR bandpass power (averaged across all channels). (2) Aligning a fixed-length window of mean bandpass LFP around the power peak using cross-correlation maximization, with a shift limit of 5 ms (10 frames at 2 kHz) to maintain phase consistency at 200 Hz. Aligned SWR events were used for frequency decomposition via wavelet transformation to identify the site of maximum ripple power across all LFP channels along the dorso-ventral axis. The CA1 pyramidal layer (PyL) was identified as the peak ripple site (184.14 Hz), serving as the reference point (0 µm) for subsequent analyses. To detect theta segments, the Matlab script for theta-state detection based on power-based segmentation was adapted from the Buzsáki Lab’s buzcode repository (GPL‑3.0)

The LFP signal was then bandpass-filtered (4–10 Hz), and theta cycles were identified using the Hilbert transformation. Theta peaks (0° phase) in PyL served as the reference for computing phase differences along the dorso-ventral axis.

### Identification of the hippocampal fissure via theta power amplitude analysis along the dorso-ventral axis

To identify significant changes in theta amplitude along the electrode axis, we implemented a computational approach in MATLAB comprising the following steps (1) The normalized amplitude of the signal was calculated across all channels, and the mean and standard deviation were computed. The maximum power was identified by locating the peak value in the averaged normalized amplitude of the theta oscillation on all channels. (2) A decline threshold was calculated to identify the point of a significant decline in amplitude after the maximum theta power peak. This threshold was defined as the mean difference between consecutive amplitude values minus three times the standard deviation of these differences. (3) Starting from the index of the peak of the theta power, the algorithm iterated through subsequent amplitude values to detect the first point where the difference between consecutive amplitudes fell below the calculated decline threshold. This point was defined as the onset of a significant decline. (4) A one-sample *z*-test was performed at the identified decline point to statistically validate the significance of the decline. The test compared the absolute difference between consecutive amplitude values at this point against the distribution of all amplitude differences. The following parameters were extracted: z-value, *p*-value, and Confidence intervals. (5) The algorithm reported the index of the decline onset, the corresponding distance along the electrode axis, and the amplitude value.

### Hierarchical clustering analysis

Hierarchical clustering was performed on a symmetric correlation matrix to identify meaningful groupings among variables and visualize relationships in a dendrogram. The steps in the analysis are detailed below: (1) The spike trains of n neurons were binned into discrete time intervals to generate a binned activity matrix *A*, where ${A}_{i,t}$ represents the spike count of neuron i in time bin t (bin size 10 ms). (2) To account for differences in spike patterns across neurons, the activity matrix was normalized by z-scoring the binned spike counts across time. (3) A pairwise correlation matrix (C) was computed from the z-scored spike count matrix to quantify the similarity of activity patterns between neurons. To remove self-similarity, the diagonal elements of the correlation matrix were set to zero. (4) The correlation matrix was transformed into a distance matrix using 1 − *C*. The upper (or lower) triangle of the distance matrix was then extracted and converted into a condensed distance vector using the squareform function. This vector was used as input for hierarchical clustering via the linkage function. The optimal threshold for cluster separation was determined using the elbow method. The distances between successive merges were extracted from the third column of the linkage matrix, and the first-order differences ($\varDelta {d}_{i}$ = ${d}_{i+1}-{d}_{i}$) were calculated. This index is determined as:

9 $${elbowIndex}={\arg }\;{\max }_{i}\triangle {d}_{i}$$

Additionally, anatomical labels were assigned based on MRID-based-localized channels, corresponding to the neurons recorded from contact sites, thereby providing spatial context for identifying the putative anatomical labels of the clusters.

### Functional–anatomical concordance (overall classification accuracy)

To quantify the agreement between functional labels (see hierarchical clustering) and MRID-based anatomy, we defined overall classification accuracy as the proportion of neurons whose MRID-based anatomical labels (assigned via MRID-tag localization and WHS-atlas registration) matched the functional clusters obtained from hierarchical clustering of spike-train correlations. (*N*_*match*_ − number of neurons with matched anatomical labels, *N*_*total*_ - total number of neurons).

10 $${Accuracy}=\frac{{N}_{{match}}}{{N}_{{total}}}\times 100$$

### PCA-K-Means clustering of Z-Scored firing rates during SWR

PCA was performed on the neuronal population activity matrix to reduce its dimensionality while preserving the majority of the variance. This technique transforms the original high-dimensional dataset into a lower-dimensional space by identifying principal components—linear combinations of the original variables—that capture the maximum variance.

To retain the most meaningful structure of the data while minimizing information loss, we selected the minimum number of principal components (PCs) required to explain 95% of the total variance. This approach balances the need for reducing dimensionality with maintaining the integrity of the dataset. Following PCA, k-means clustering was applied on the selected principal components to categorize neuronal activity patterns during SWR. The Calinski-Harabasz criterion was used to determine the optimal number of clusters, ensuring that within-cluster variance was minimized while between-cluster variance was maximized. The final output consisted of cluster labels assigned to neurons based on their firing activity.

This combined approach of PCA-based feature extraction and k-means clustering enables an efficient and interpretable classification of neuronal population activity, facilitating a better understanding of how different neurons respond to varying around the SWR events. A 50 ms window centered on SWR events was compared to a 50 ms baseline window of spontaneous activity (250 ms earlier then SWR event) using a two-sample t-test (ttest2) with a significance level (*α*) set to 0.01.

### Electrolytical lesioning

We electrolytically lesioned the brain from selected and working channels (impedance magnitude at 1 kHz <5 MΩ) briefly before the perfusion. We applied, first, +7 µA direct current (DC) for 5 s, and then -7 µA DC for 5 s. After applying DC from each selected channel, we waited for 45 min for lesion formation in the tissue. The lesioning protocol was adapted from a protocol released for silicon probes *(Micro-lesioning protocol, NeuroNexus Technologies Inc.)*. We confirmed the lesions with post-mortem immunohistology.

### Chronic MRID contrast analysis

In one animal (Animal #1), the MRID-tag spanned the entire UFTE bundle along the implantation trajectory, which induced a single-body contrast. In MRI sessions of that animal, we aligned three imaging-slices (136 × 136 µm pixel dimensions, 800 µm thickness) in axial (i.e., horizontal) orientation that slices the trajectory horizontally. The other MRID-tags (**n** = 5, animals #2 and #3) were imaged with a single slice in coronal or sagittal orientation (136 × 136 µm pixel dimensions, 800 µm thickness). If an IONP island was imaged in multiple orientations, we chose the orientation that gives the maximum contrast. We sorted the measured contrast intensities within each IONP island in descending order. Then, we calculated the mean contrast intensity from the five most contrastive pixels in each IONP island in each session.

### Perfusion, brain sectioning and immunostaining

At the end of the in vivo experiments, animals were euthanized by intraperitoneal injection of sodium pentobarbital (300 mg/kg; Esconarkon, Streuli Tiergesundheit AG). Once deep anesthesia was confirmed, transcardial perfusion was performed with 1× PBS (pH 7.4, Gibco, Thermo Fisher Scientific, Cat. No. 10010056) followed by 4% paraformaldehyde (PFA) in PBS, freshly prepared from powder (Merck, Cat. No. 158127). Brains were carefully extracted, post-fixed overnight in 4% PFA, and washed three times in PBS. The perfusion and fixation protocol has been described in detail previously^9^.

Following fixation, rat brains were horizontally sectioned into 100 µm-thick slices using a vibratome (Leica VT1200S) at a speed of 50 µm/s. Sections were stored overnight in PBS 1x (pH 7.4, Gibco, Thermo Fisher Scientific, Cat. No. 10010056) at 4 °C before immunostaining. We selected 100 µm-thick slices to preserve 3D tissue architecture, which was essential for analyzing spatial fluorescence gradients around implants. Thicker sections minimized tissue loss and mechanical damage, while long working distance, high-NA objectives (Olympus UPLXAPO 20×/0.80 air, WD 0.61 mm; UPLSAPO 30×/1.05 silicon, WD 0.8 mm) enabled imaging through the full slice depth. Uniform antibody penetration was ensured through permeabilization and extended incubation steps (2–3 days).

For electrode localization, we left the electrode bundles inside the brain in Animal #2, which was used for astrogliosis analysis. Nevertheless, slight displacements of electrodes may have occurred during dissection and sectioning. For Animal #1 and Animal #3, electrodes could not be retained because they were fully embedded in the bone after ~5 months of implantation. For these animals, we performed electrolytic lesioning to identify electrode trajectories post-mortem.

For immunostaining, free-floating sections were first incubated in a permeabilization solution containing 0.3% Triton X-100 in PBS (pH 7.4, Gibco, Thermo Fisher Scientific, Cat. No. 10010056) for 30 min at room temperature with gentle shaking. Blocking was then performed using a buffer containing 1% Bovine Serum Albumin (BSA) and 0.3% Triton X-100 in PBS for 2 h at room temperature. Primary antibodies, Rabbit anti-IBA1 (019-19741, FUJIFILM Wako) for microglia and Chicken anti-GFAP (PA1-10004, Thermo Fisher) for astrocytes, were diluted to a concentration of 1:1000 in the blocking buffer and applied to the sections. The sections were incubated for 3 days at room temperature.

After the primary antibody incubation, the sections were washed once with 0.3% Triton X-100 in PBS, followed by three 10-min washes with PBS alone. For secondary antibodies, either Donkey anti-Rabbit Alexa Fluor 488 (A48283, Thermo Fisher) or Goat anti-Rabbit Alexa Fluor 555 plus Fab2 (A48283, Thermo Fisher) was used to detect Rabbit anti-IBA1, while Goat anti-Chicken Alexa Fluor 647 (A32933, Thermo Fisher) or Goat anti-Chicken Alexa Fluor 647 (A32931, Thermo Fisher) was used to detect Chicken anti-GFAP. The secondary antibodies were diluted to a concentration of 1:500 in the blocking buffer and applied to the sections, which were then incubated for 2 days at room temperature. Following this incubation, the sections were washed once with 0.3% Triton X-100 in PBS, followed by three 10-min washes with PBS alone.

The sections were then counterstained with either Neurotrace 530/615 Red Fluorescent Nissl Stain (N21482, Thermo Fisher) or Neurotrace 640/660 Fluorescent Nissl Stain (N21483, Thermo Fisher) at a concentration of 1:200 overnight. Subsequently, the sections were washed once with 0.3% Triton X-100 in PBS, followed by three washes with PBS alone for 10 min each, counterstained with DAPI at a concentration of 1:1000 for 30 min, and then washed once more with 0.3% Triton X-100 in PBS and three times with PBS alone for 15 min each. We chose fluorescent Nissl staining (NeuroTrace) instead of NeuN or MAP2, as it provided unbiased labeling of nearly all neuronal somata. NeuN labels only subsets of neurons, while MAP2 highlights dendrites rather than cell bodies. NeuroTrace enabled reliable neuronal counts within concentric analysis zones around implants.

After final washes, sections were mounted on Superfrost glass slides (Thermo Scientific Menzel-Gläser) using iohexol as the mounting medium, covered with rectangular coverslips (24 × 50 mm, No. 1, Menzel-Gläser) and sealed with nail polish.

### Imaging of histological brain slices and statistical analysis

Imaging of the prepared brain sections was performed using an Olympus IXplore Spin spinning disk confocal microscope. Serial 100 μm-thick brain slices were initially scanned with the Olympus UPLXAPO 4×/0.16 air objective to obtain single-plane overview images of the entire brain slice. This initial scan allowed for the precise localization of electrodes within the brain. High-resolution z-stacks of the identified electrode implantation sites were then acquired using either the Olympus UPLXAPO 20×/0.80 air objective or the Olympus UPLSAPO 30×/1.05 silicon objective. These high-resolution scans were used to quantify microglia, astrocytes, and neurons in the vicinity of the electrodes within the brain tissue.

The confocal setup utilized a Prime BSI Scientific sCMOS camera to capture 16-bit images at a resolution of 2048 × 2048 pixels. Fluorescence excitation was achieved with laser lines at 405, 488, 561, and 640 nm, and exposure times varied from 200 to 300 ms depending on the channel. Laser intensities were adjusted between 5% and 20%, corresponding to 0.5 to 5 mW, to optimize imaging conditions. Z-stacks were acquired with a z-step size of 5–10 μm.

From the borders of UFTE cross-sections, equidistance circular contours (40 μm separation, until 320 μm) were defined (Fig. 5c). We calculated the mean fluorescence intensity (FI) in all three imaging channels for the areas defined by eight consecutive contours (**n** = 13 brain slices). The mean FIs were normalized to the mean of a large reference area (>700 μm away from the UFTEs). To define the control condition, we handpicked a region further away from (700 μm) the UFTEs and defined identical contours. The mean FI from the control condition was also normalized. We masked the big vessels in the images. Welch’s two-sample t-test is applied at each circular area defined by the contour between the ROI (i.e., around the UFTEs) and the control. Raw immunostaining images are provided for all **n** = 13 brain slices in Supplementary Fig. 22.

### Statistics and reproducibility

We tested the dot-matrix nanoparticle coating (with optimized parameters e.g., coating speed) on three samples. Each sample contained 21 ultra-flexible electrode bundles, and nanoparticle coating was applied independently to each bundle (i.e., one ultra-flexible bundle coated at a time). The electron micrographs and optical microscopy images were acquired multiple times for each sample and for majority of ultra-flexible electrode bundles. Each ultra-flexible electrode bundle accommodates thousands of IONP dots. Different IONP dot diameters were evaluated using the same procedure. The results shown in Figs. 1b, 2d-f, Supplementary Fig. 2b–e were pooled from multiple samples. The corresponding panels are presented for qualitative demonstration only.

Z-test and Student’s two-sample t-test (two-sided) were performed to analyze electrophysiology data. Student’s two-sample t-test (dependent samples, two-sided) was performed to analyze chronic MRID-induced contrast data. The normality of the data was verified with Shapiro-Wilk test. Welch’s test (two-sided) was performed to analyze the immunohistology results. The statistical analyses were performed using MATLAB (MathWorks, Natick, MA, USA) and SciPy package for Python 3 (https://scipy.org). No statistical method was used to predetermine sample sizes. The experiments were not randomized. The sharp-wave ripple and theta oscillation detection and the contributing author in charge of the manual curation were blinded to the sharp-wave ripple power, theta oscillation power and phase analyses. The electrophysiology-based electrode localization analysis was performed by a different contributing author than who performed the electrophysiology data preprocessing. The MRID-based electrode localization analysis was performed by a different contributing author than who performed electrophysiology-based electrode localization analysis.

### Reporting summary

Further information on research design is available in the Nature Portfolio Reporting Summary linked to this article.

## Supplementary information

Supplementary Information

Description of Additional Supplementary Information

Supplementary Video 1

Reporting Summary

Transparent Peer Review file

## Source data

Source Data

## Data Availability

All data supporting the findings of this study are available within the article and its supplementary files. Additional data is deposited to the Zenodo repository at: 10.5281/zenodo.18917094^55^. Any additional requests for information can be directed to, and will be fulfilled by, the corresponding author. Source data are provided with this paper.

## Code availability

All custom code and preprocessed data used in this manuscript are available at the GitHub repository https://github.com/Neurotechnology-at-ETH-Zurich/MRID-code and on Zenodo 10.5281/zenodo.18917094^55^.
