# Supplementary material for: Magnetic resonance identification tags for ultra-flexible electrodes
Source: Nat Commun. 2026 Apr 28;17:5725. doi: 10.1038/s41467-026-71887-x (PMC13324162; doi:10.1038/s41467-026-71887-x)
Supplement: Supplementary file 1 — Supplementary Information [file 41467_2026_71887_MOESM1_ESM.pdf]

Supplementary

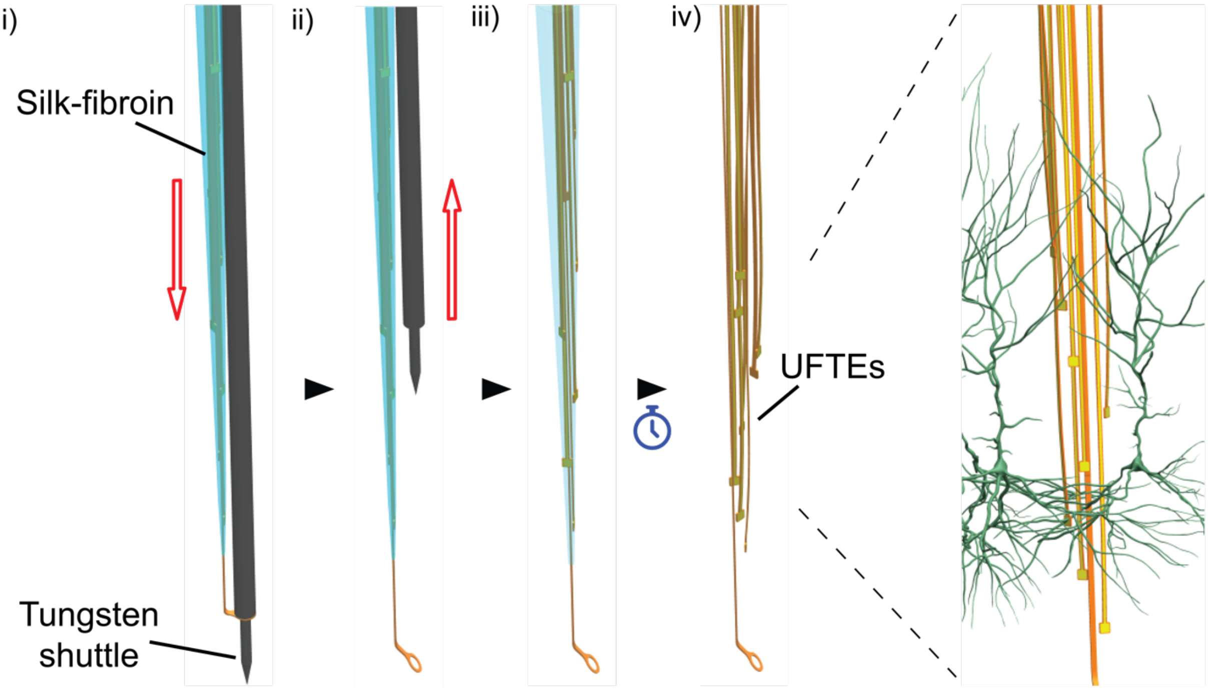

Suppl. Fig. 1. Insertion technique of UFTEs. i) UFTE bundles are coated with silk-fibroin and attached to a tungsten insertion shuttle via their loop-shape hook at the tip. Tungsten shuttle guides the UFTE bundle into brain at arbitrary depths. **ii)** Tungsten shuttle is retracted as the desired implantation depth is achieved **iii)** leaving the UFTE bundle in the brain. **iv)** Silk-fibroin dissolves in the tissue by the time which yields a seamless integration of each individual tentacle fiber into tissue. Adapted from Yasar et al., Nature Communications (2024), licensed under CC BY 4.0.

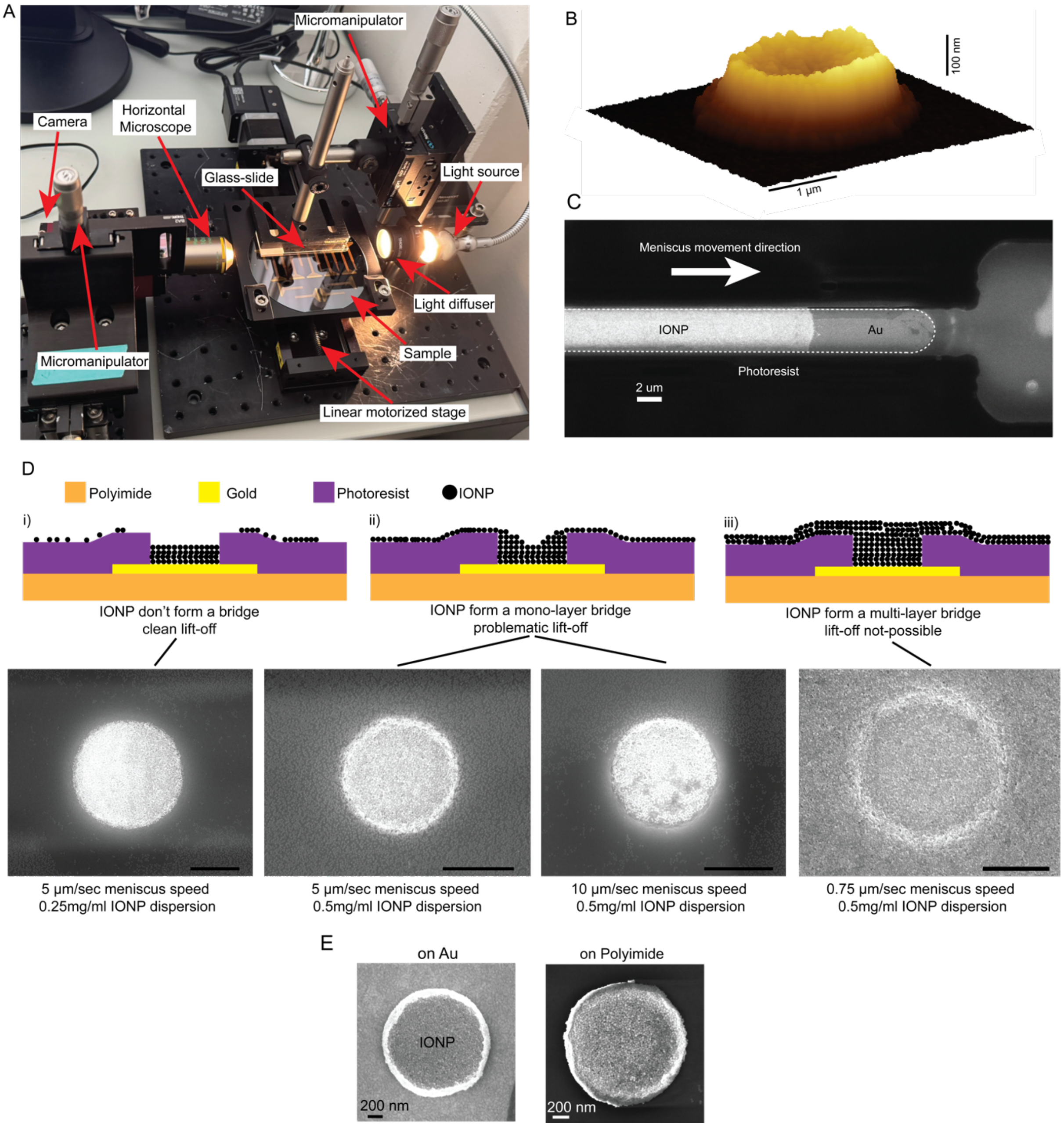

Suppl. Fig. 2. Custom-setup for IONP coating and coating characterization. **A.** IONP custom coating setup with a horizontal microscope on the side for contact angle measurement. **B.** 3D Atomic force microscopy image of a circular IONP assembly shows the crater-like shape. **C.** Dot-matrix CCA coating was tested with strip-shaped traps instead of dots. IONPs were coated homogeneously along the strip until the trailing edge. There is a gap (~15 µm) towards the trailing edge. **D.** Top-row shows the cross-section of the sample for possible outcomes of IONP coating at different meniscus speeds and IONP dispersion concentrations. Bottom row shows the exemplary SEM images for each outcome at given coating parameters. Scale bars denote 1 µm. **E.** IONP dots on unroughened Au and Polyimide surfaces.

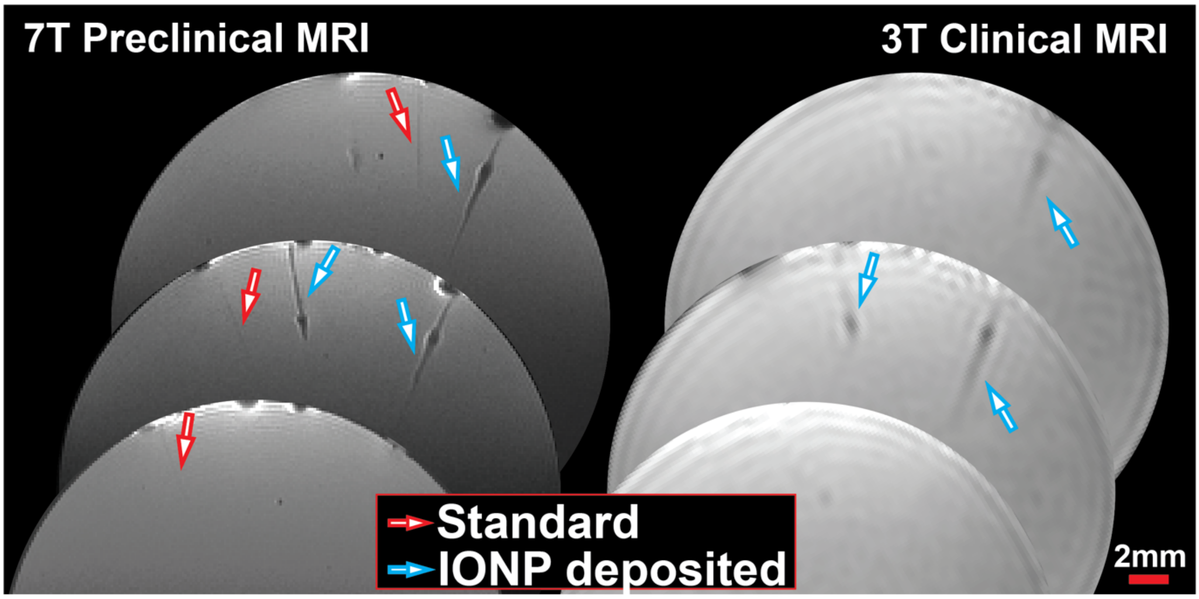

Suppl. Fig. 3. Four UFTE bundles are implanted into agarose gel phantom (n=2 IONP deposited, n=2 standard). Phantom is imaged in both 7T preclinical and 3T MRI scanners.

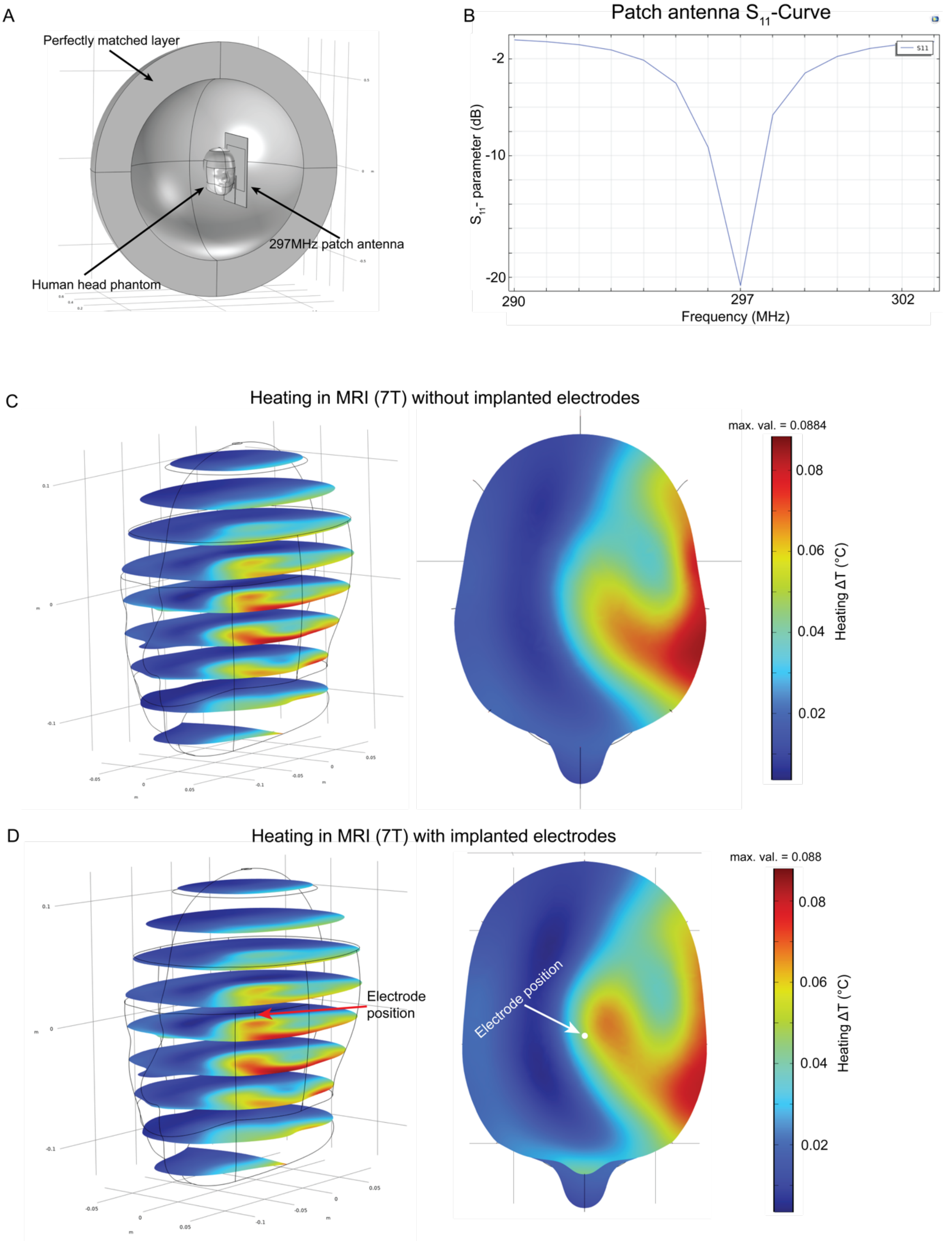

Suppl. Fig. 4. RF-induced heating of human-phantom in 7T MRI with and without the MRI-tagged UFTE electrodes under the RF-excitation frequencies used in this study. **A.** Full-wave simulation setup shows the perfectly matched layer, human head phantom and patch antenna placement. **B.** Custom patch antenna which is operating at 297MHz (7T MRI H1 frequency). **C.** Heating in MRI without MRI-tagged UFTE electrodes. **D.** Heating in MRI with MRI-tagged UFTE electrodes.

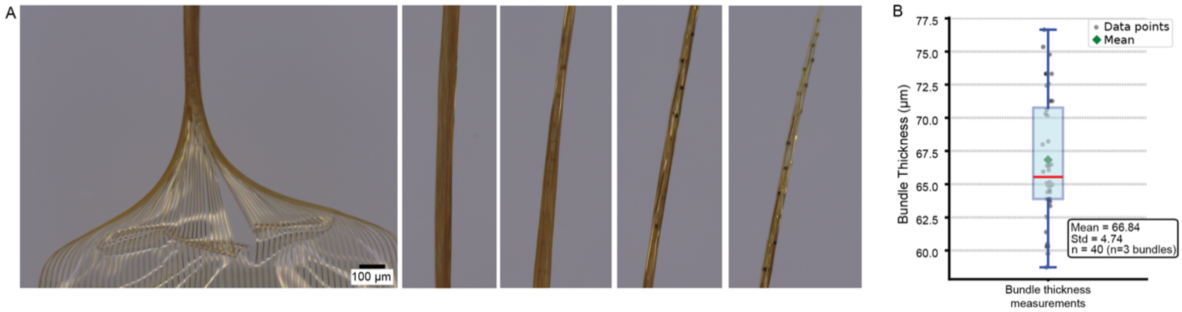

Suppl. Fig. 5. Silk-fibroin coated MRID-tagged UFTE bundle thickness measurement. **A.** Optical microscopy images of the electrode bundle from dorsal to ventral (left to right). **B.** Bundle thickness measurements (all 64-fibers) box plot and descriptive statistics (n=40 measurement points, n=3 electrode bundles).

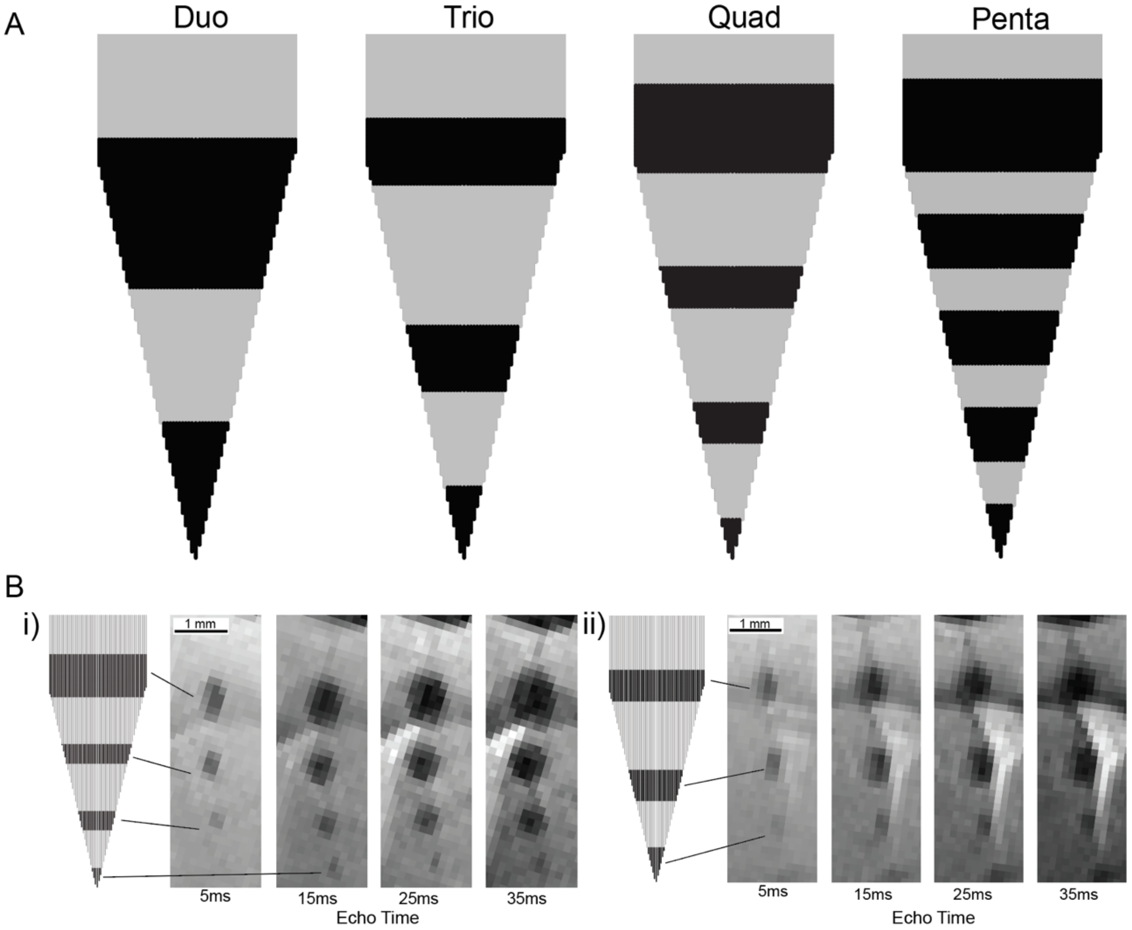

Suppl. Fig. 6. A. MRI-barcode designs used in this work; *duo, trio, quad,* and *penta* **B.** Raw MRI T2*-MGE images of two sample MRID-tagged UFTE bundles at increasing echo times (TEs). MRID-tag with the **i)** quad-pattern MRI-barcode and **ii)** the trio-pattern MRI-barcode.

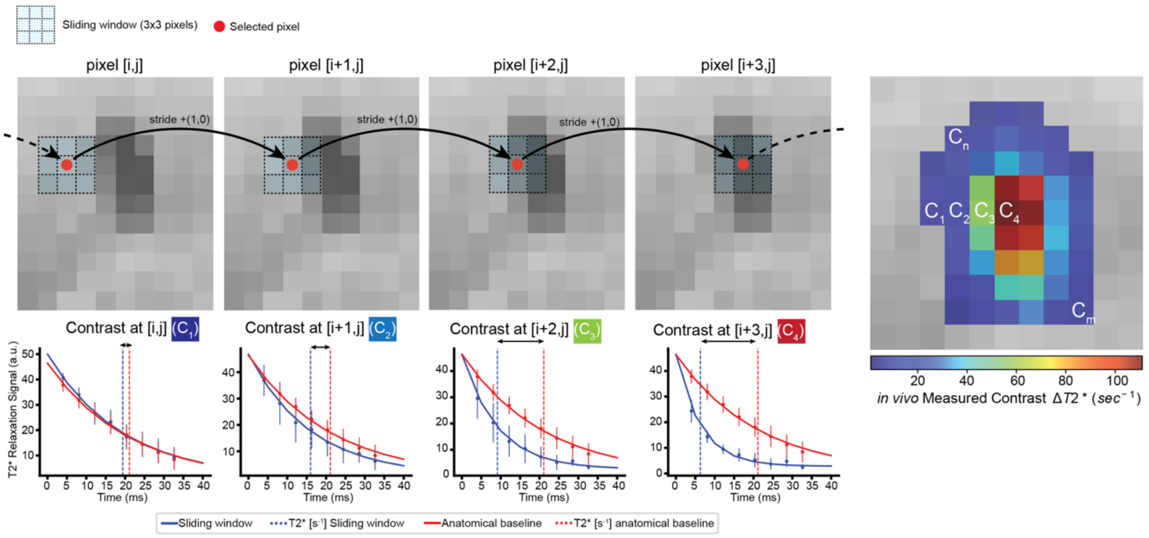

Suppl. Fig. 7. MRID Analysis, contrast heatmap generation. A sliding window is moved over (in both x- and y- axes, with stride:1) where the MRID contrasts are. At each pixel, mean pixel intensities from sliding window are compared to the mean pixel intensities from anatomical baseline across increasing echo times. Relaxivity time constants are calculated for MRID and baseline curves (mean ± SD pixel intensities, n = 9 pixels for sliding window, n > 100 pixels for baseline), where the difference gives the contrast intensity. A heatmap is generated from each measured contrast intensity.

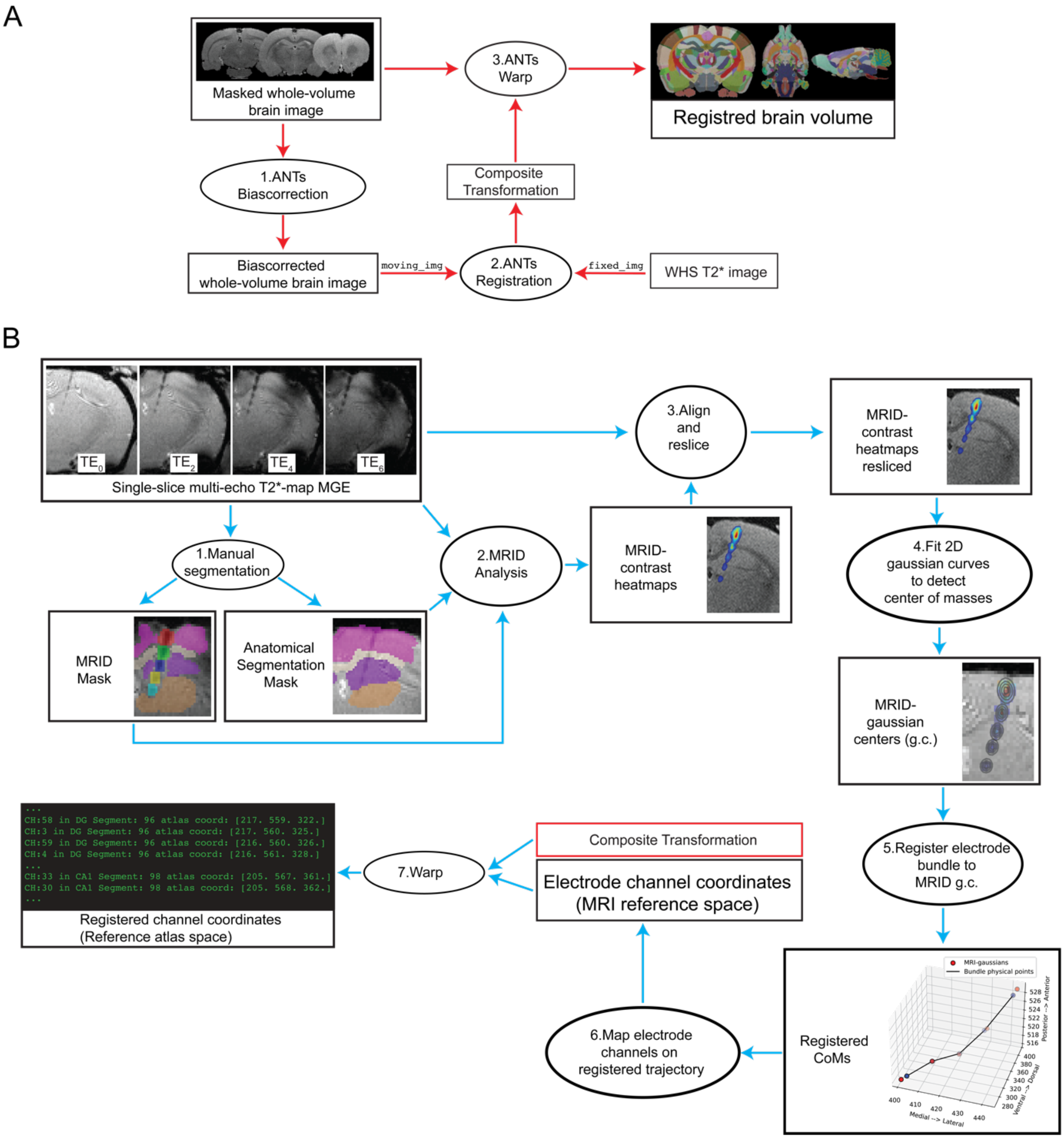

Suppl. Fig. 8. MRI image registration and MRID-tag image analysis pipeline explained. **A.** Whole-volume brain MR images are biascorrected and registered to WHS reference atlas outputting a composite (i.e. rigid, affine and elastic transformations combined) transformation. **B.** Single-slice multi echo time T2*-map MGE images of MRID-tags are analyzed with custom MRID analysis pipeline. Images are manual segmented to define region of interests for MRID-tags and anatomy. Contrast heatmaps are generated for each MRID tag and Gaussian curves are fit around the heatmaps which estimates the CoMs of IONP islands. Point-set registration finds the best fit bundle trajectory explaining the MRID-tag induced contrast in MRI. Electrode channels are mapped on the best fit bundle trajectory using the exact design layout dimensions. Composite transform from reference atlas registration is used to map each electrode channel into reference atlas space to identify the anatomical location.

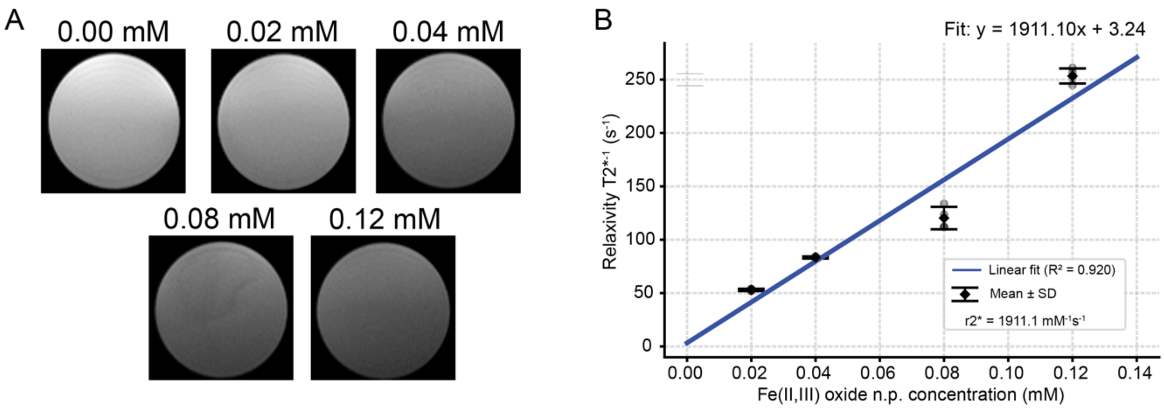

Suppl. Fig. 9. Characterization of induced contrast by IONP in 7T MRI at varying concentrations. **A.** T2*-MGE (TE step = 4.09ms) MRI images of phantoms at varying IONP in dH_2O concentrations. **B.** T2* relaxivity of IONP (25 nm avg diameter). Mean measurements are shown with **black** scatter plots (n=4 MRI imaging slice at each concentration). **Blue** line shows the best fit giving the relaxivity constant (i.e. *r2**) of IONP.

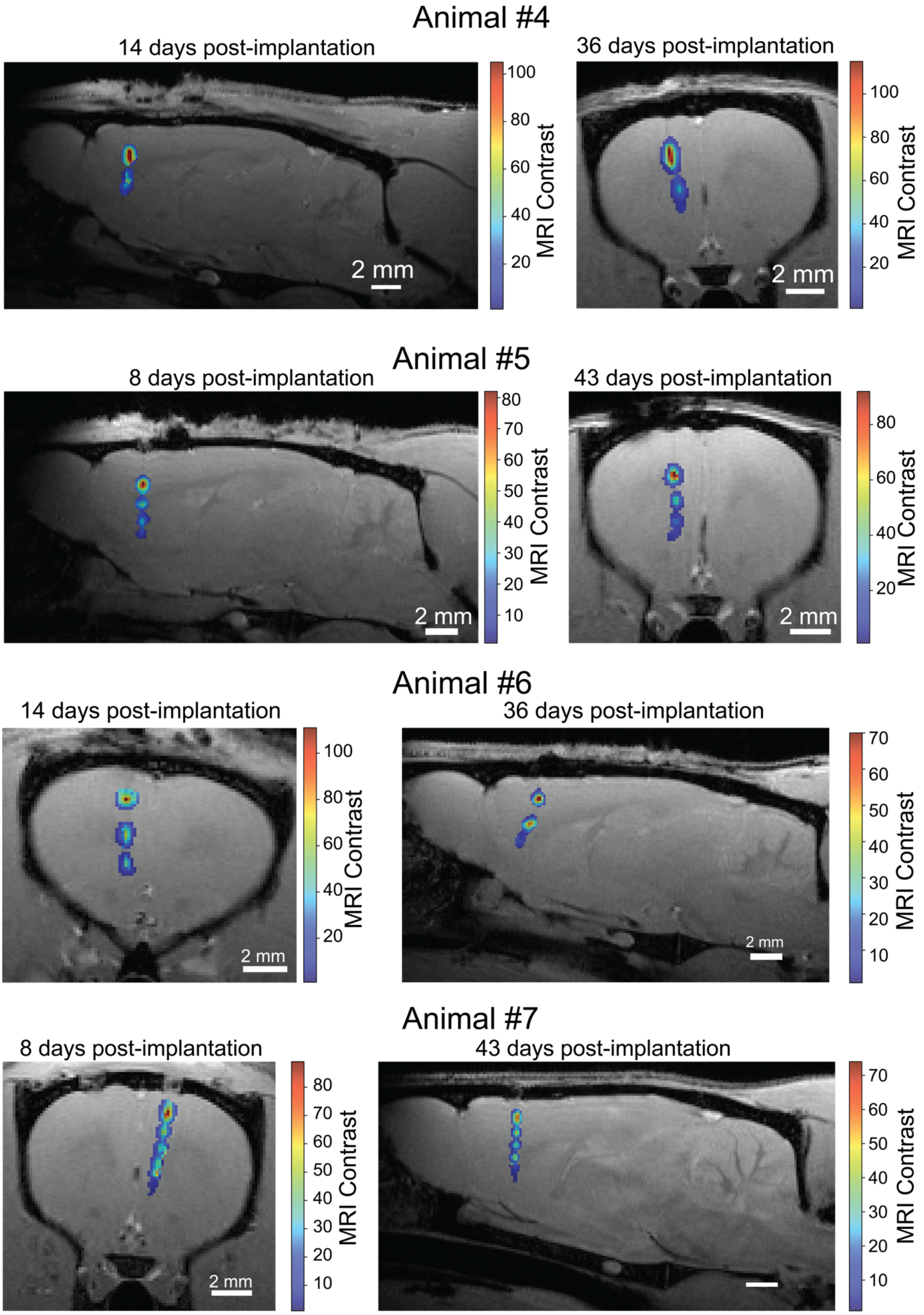

Suppl. Fig. 10. Four examples for chronically implanted MRID-tagged UFTE bundles to show the contrast heatmaps across animals, bundles and time.

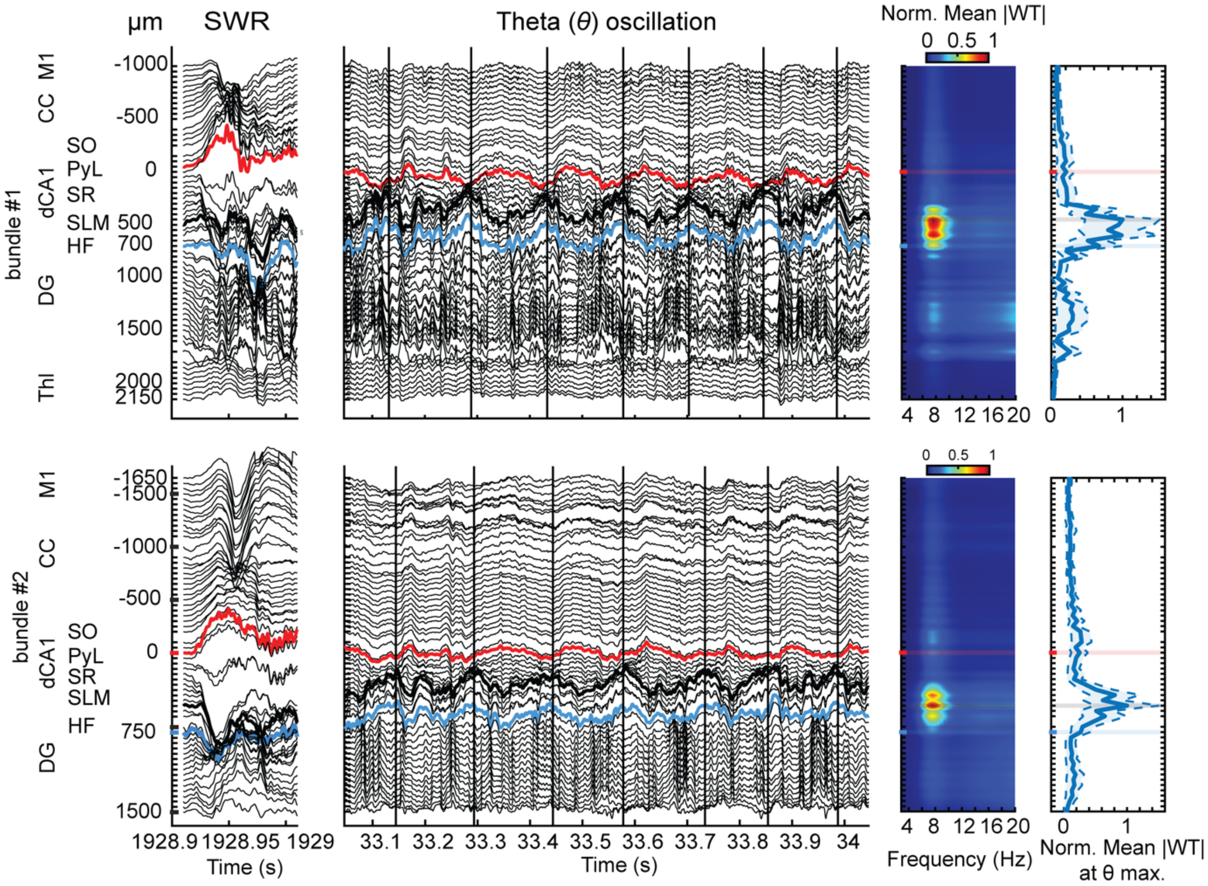

Suppl. Fig. 11. The first column shows a representative LFP traces (1–300 Hz) containing a sharp wave ripple (SWR). The second column displays a simultaneous 1-second LFP segment (1–300 Hz) illustrating theta oscillations recorded from bundle #1 (left hemisphere, top row) and bundle #2 (right hemisphere, bottom row). The x-axes of the rows are aligned, so that each tick mark represents the same time point across rows, ensuring that the SWR trace and the theta-segment LFPs correspond to simultaneous activity in the left and right hemispheres. The vertical solid black line indicates the peak of the theta oscillation in SLM. The third column presents the averaged continuous wavelet transformation of the theta segments (n = 2306 cycles) across all contact sites of the bundle. The fourth column shows the mean wavelet transformation (solid line) and ±SD (dashed lines) at the point of maximum theta power across all contact sites of the bundle. The y-axis represents the distance between contact site channels in μm. In the raw LFP data, red lines indicate the beginning of the pyramidal layer in the CA1 dorsal hippocampus, the blue line marks the hippocampal fissure (HF), and the thicker black line indicates the location of maximum theta power in SLM.

Abbreviations: M1 – motor cortex; CA1 – Cornu Ammonis 1; DG – dentate gyrus; SO – stratum oriens; PyL – pyramidal layer; SR – stratum radiatum; SLM – stratum lacunosum-moleculare; HF – hippocampal fissure.

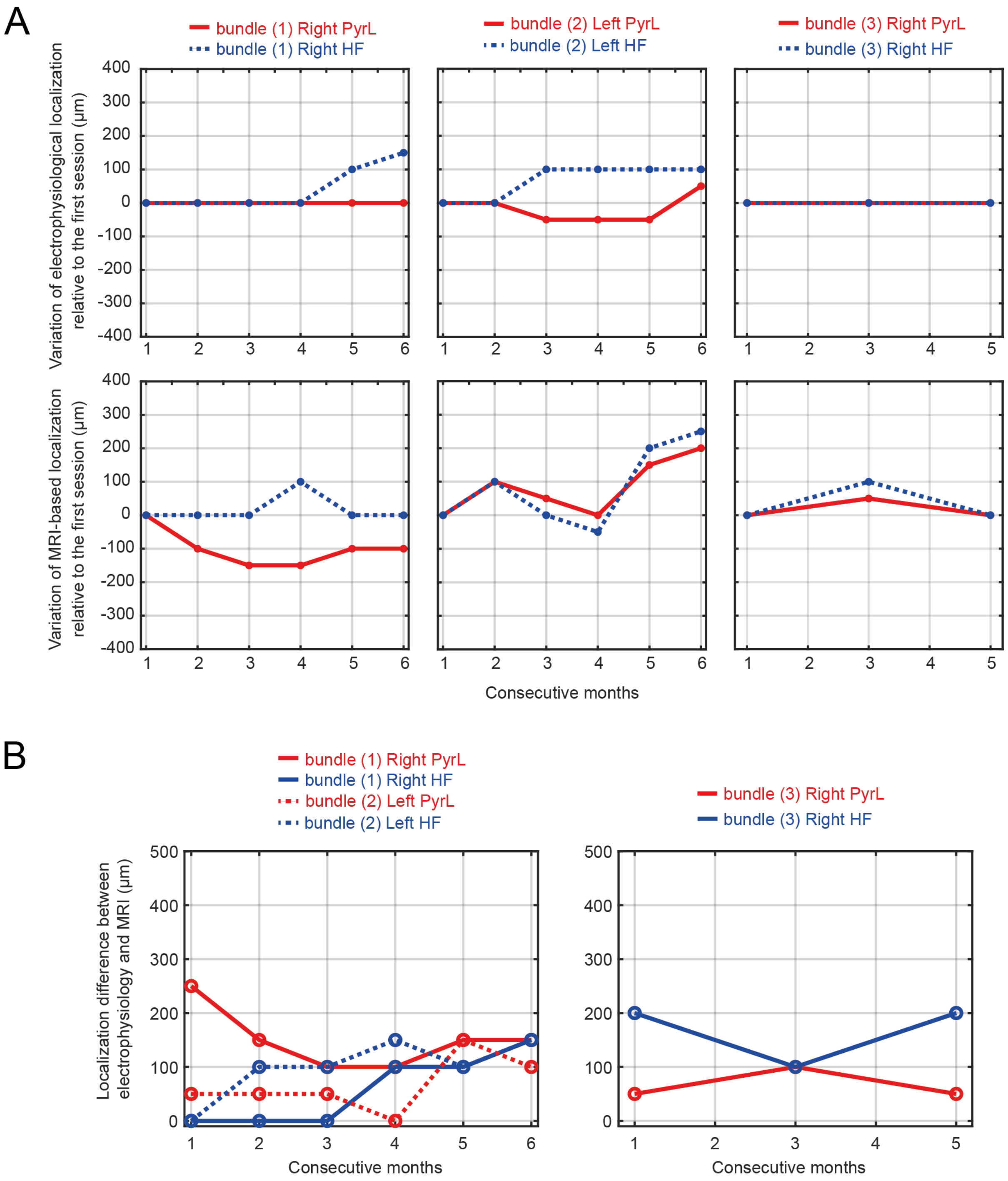

Suppl. Fig. 12. A. Top row: Three representative electrode bundles showing localization consistency across sessions based on electrophysiological (LFP-derived) landmarks, referenced to the first recording session (0 µm). The solid red line marks the CA1 pyramidal layer, and the dashed blue line indicates the hippocampal fissure at each measurement time point. Bottom row: Variation of consecutive MRI-based localization across sessions relative to the first session (0 µm reference). B. Localization differences between MRID–based localization and electrophysiological landmarks derived from LFPs, including the CA1 pyramidal layer (red) and the hippocampal fissure (blue). Solid lines denote the right hemisphere, and dashed lines denote the left.

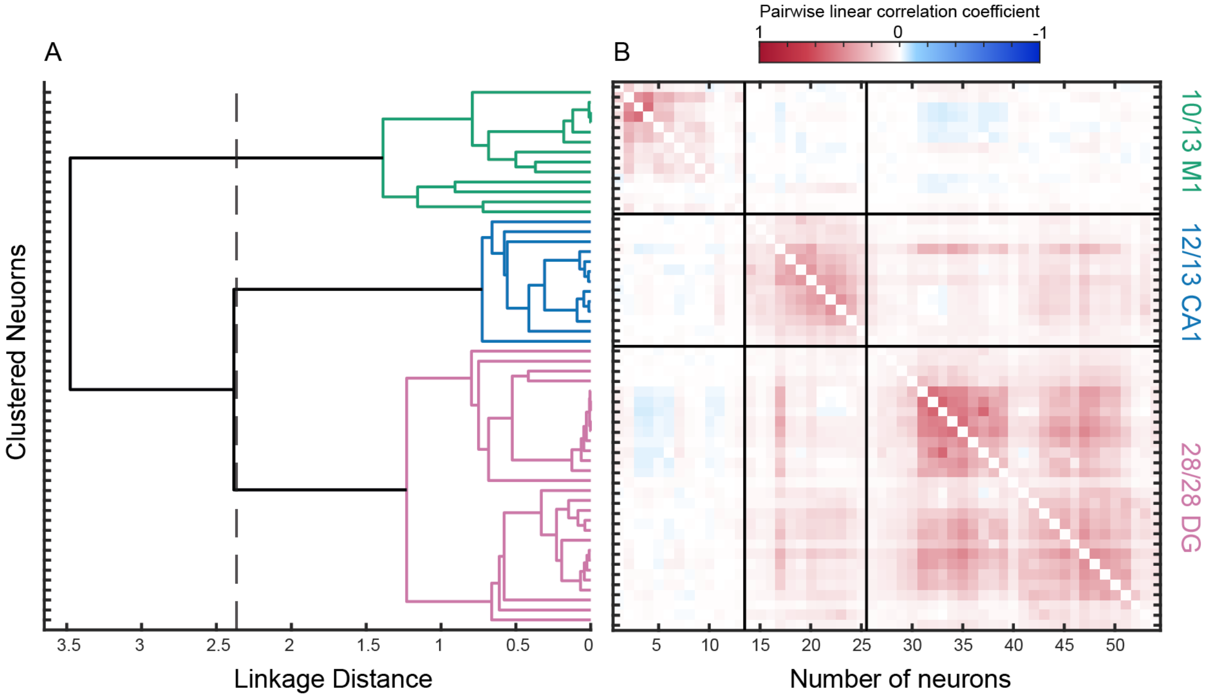

Suppl. Fig. 13. Hierarchical clustering of pairwise correlation of neuronal activity. A. Dendrogram of clustered neurons based on pairwise correlation of z-scored firing activity (bin size: 25ms). Hierarchical clustering was performed using the Ward linkage method, grouping neurons according to their pairwise correlation patterns. The x-axis represents the linkage distance, while the y-axis denotes individual neurons sorted by clustering results *Note: This does not represent the dorsoventral (D-V) axis anymore, as in Figure 4F*. The vertical dashed line indicates the threshold determined by the elbow method, which optimally defines the number of clusters. **B.** Heatmap of the correlation matrix displaying pairwise correlations between neuronal firing rates. Warmer colors indicate higher correlations, while cooler colors reflect weaker or negative correlations. Clusters identified in (A) represent functionally similar neuronal groups, delineating anatomical structure. The numbers on the right side of the pairwise correlation matrix indicate how many matched neurons were found within grouped neurons, based on hierarchical clustering of pairwise correlations using MRI localization labeling.

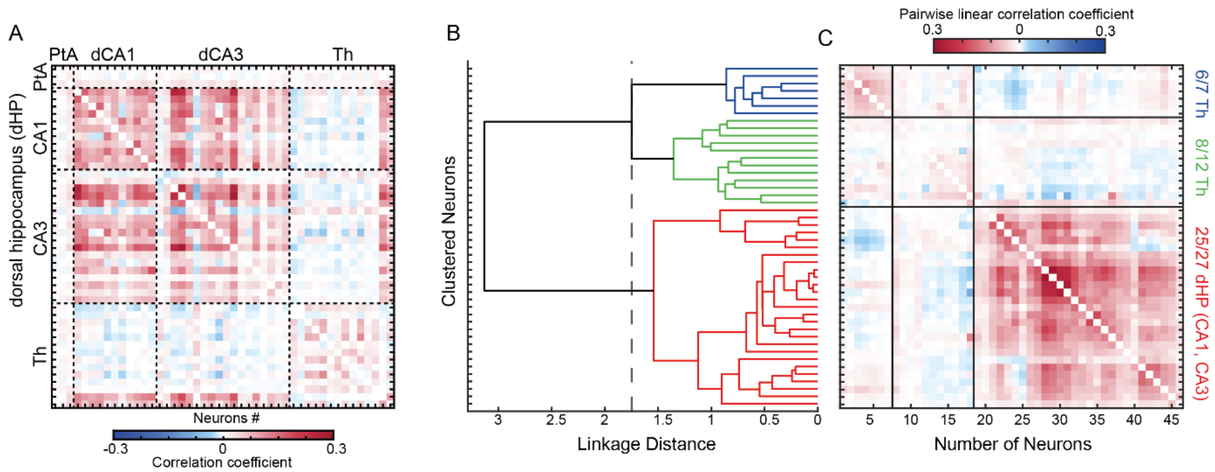

Suppl. Fig. 14. A. MRI- and function-based identification for a different bundle trajectory compared to Suppl. Fig. 4.1, with functional grouping determined by spike-train correlation and hierarchical clustering. Correlation matrix based on 25 ms binned z-scored firing rates. Neuronal IDs are derived from MRI-detected labels of contact sites (multiple neurons can originate from the same contact site). The plot preserves the dorso–ventral orientation of the brain. **B.** Hierarchical clustering of the correlation matrix. The dashed line indicates the threshold determined by the elbow method. **C**. Reordered correlation matrix according to the results of the hierarchical clustering. The hierarchical clustering revealed two thalamic clusters; units 6/7 and 8/12 were correctly labeled by MRI. In addition, 25/27 units from the dorsal CA1, identified as the third functional cluster, were correctly labeled according to the MRI localization.

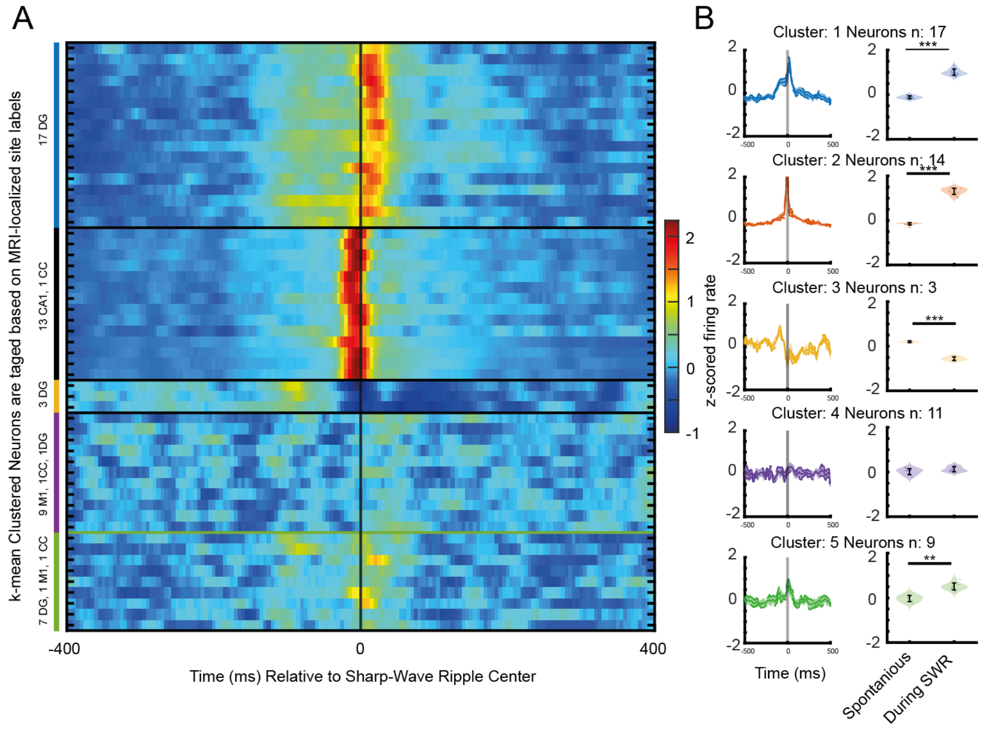

Suppl. Fig. 15. PCA based – K-means clustering of population activity during SWR. A. We identified patterns or clusters of similar neuronal activity in response to SWRs (*n* = 463). After identifying functional clusters, neurons were labeled with anatomical structures based on MRI tagging of channels. The Y-axis represents MRI-localized labels for neurons, while the X-axis shows the time interval from -400 ms to +400 ms around SWRs. **B.** In the first column, we present the average z-scored firing rate of the functionally clustered neuronal population around SWRs. In the second column, violin plots show the distribution of clustered z-scored firing rates before SWRs and during SWRs (50 ms window), with individual neuron values overlaid as dots. A t-test was used to compare firing rates between the pre-SWR and SWR periods. *p < 0.01, **p < 0.001, ***p < 0.0001.

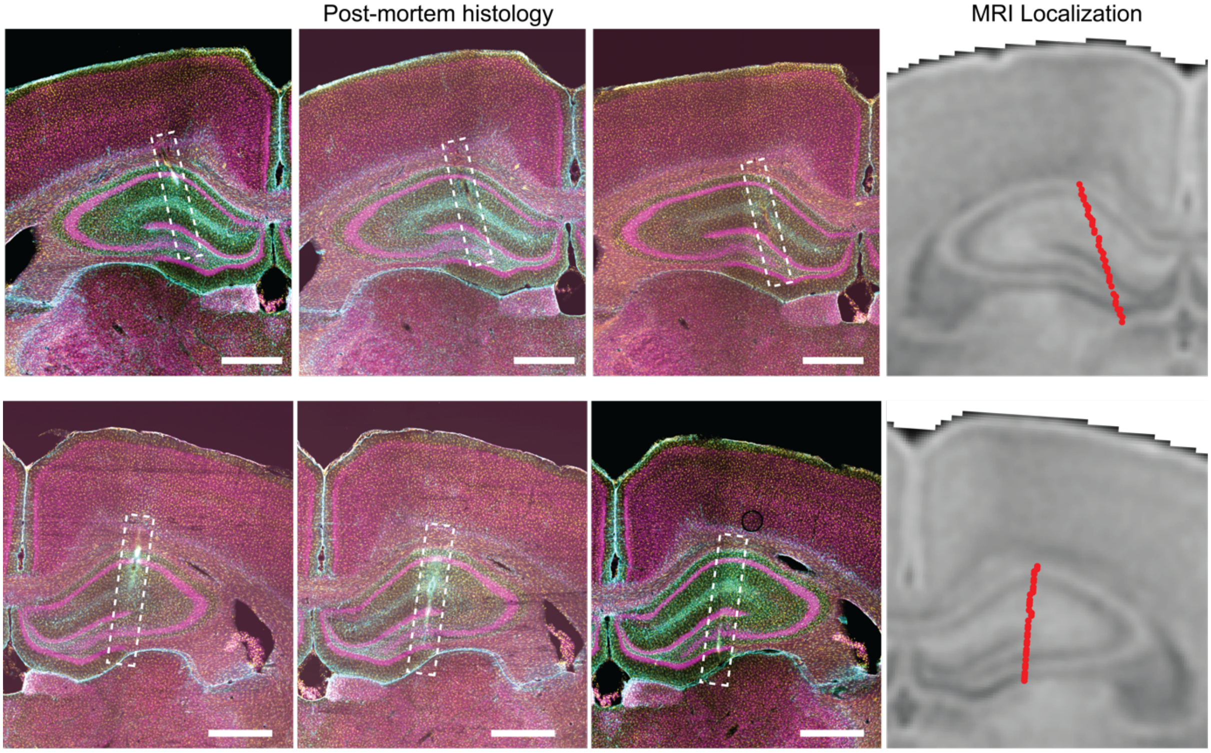

Suppl. Fig. 16. Post-mortem histology validates MRID-based electrode localization. Post-mortem histology slices with electrolytic lesions reveal the trajectory of the implantation (**white dashed-line rectangles**). Localized and registered electrode channels on the DWI WHS image (**red circles**) shows the corresponding electrode channel locations based-on MRID analysis. Scale bars denote 1 mm.

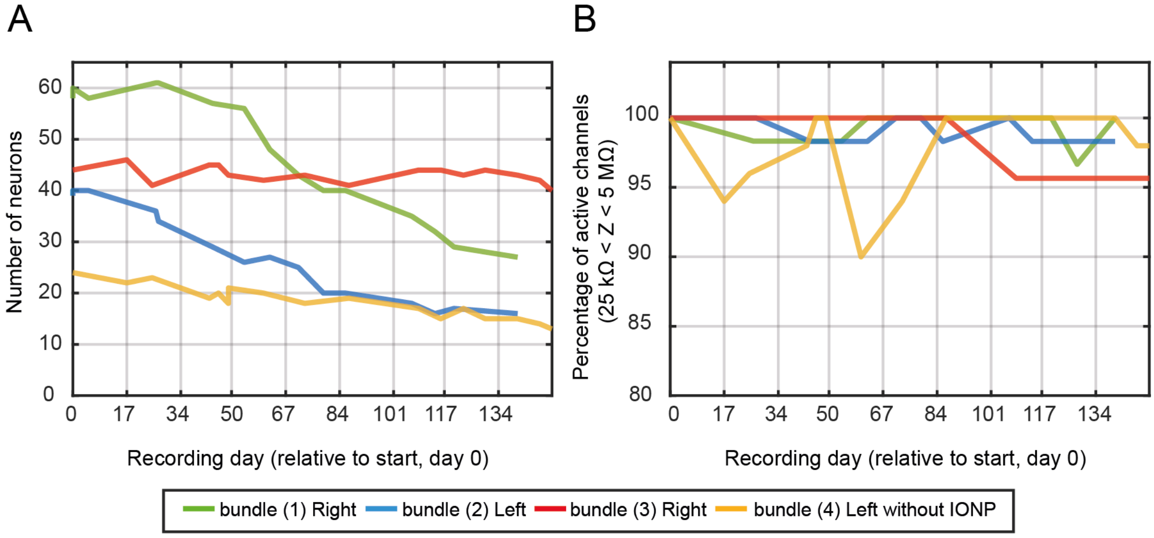

Suppl. Fig. 17. A. Number of neurons recorded across days from all bundles (color code: green – bundle 1, blue – bundle 2, red – bundle 3, and control bundle 4 without IONP).
B. Percentage of active channels (25KΩ < Impedance (Z) < 5MΩ) across days. Bundle 1: initial 60 channels = 100% active; Bundle 2: 59 = 100%; Bundle 3: 46 = 100%; Bundle 4: 50 = 100%.

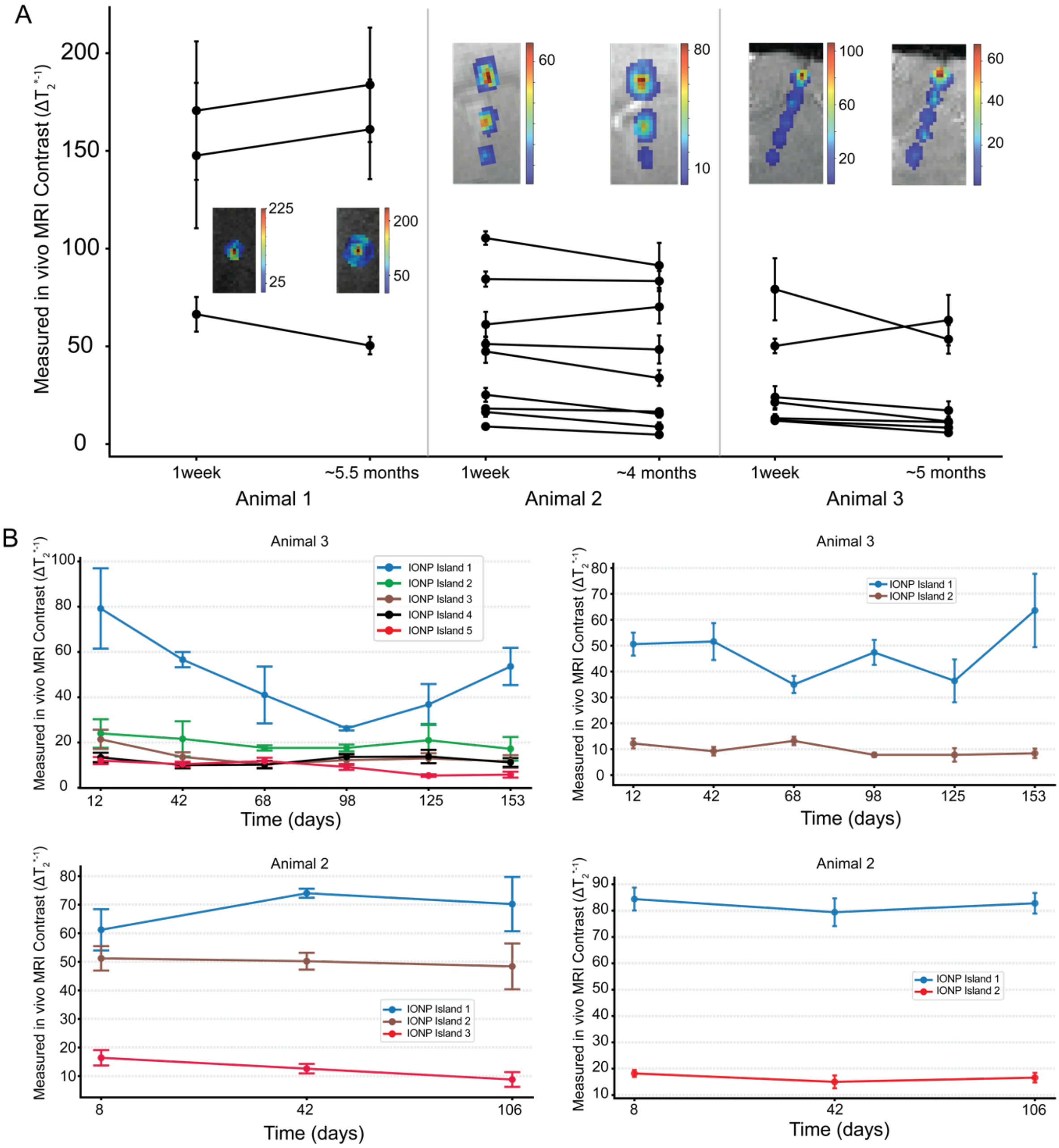

Suppl. Fig. 18. Chronic MRI contrast induced by MRID-tags. A. Post-recovery and prior to perfusion MRID-tag induced contrasts from all animals (mean ± SD, *n* = 5 pixels per point). **B.** Chronological MRI contrasts from four example MRID-tagged UFTE bundles from two animals shown.

A

	15min	30min	1h	2h	3h	4h	12h	24h	48h	72h
EO Protease	-	-	+	+	+	+	++	++	+++	+++
	-	-	++	++	++	++	++	++	++	++
Ctrl Protease	-	-	+	+	+	+	++	+++	+++	+++
	-	-	+	+	+	+	++	++	++	++
EO PBS	-	-	-	-	-	-	-	-	-	-
Ctrl PBS	-	-	-	-	-	-	-	-	-	-

B

-	bundle is intact
+	fibers disentangled slightly from bundle tip (~1mm)
++	fibers disentangled mid-bundle (~2-3mm)
+++	completely disentangled fibers (not bundled anymore)

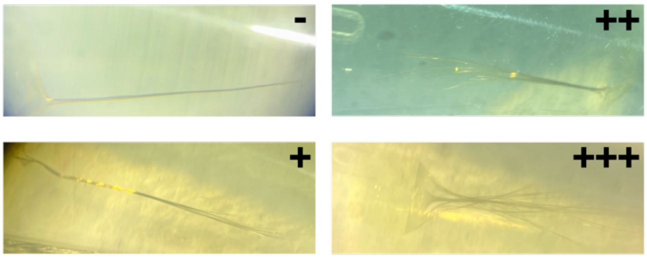

C

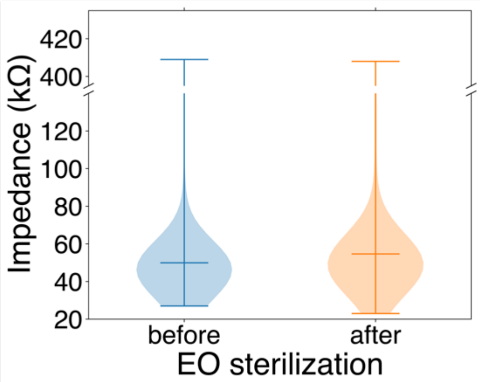

Suppl. Fig. 19 in vitro tests of sterilized MRID-tagged silk-fibroin coated UFTE bundles. **A** In vitro degradation of EO sterilized silk fibroin coating. Ctrl = Control, EO = ethylene oxide sterilization, PBS = incubated in PBS solution. **B** The scale used for the visual evaluation of the degradation. Example pictures of degradation stages are shown. **C** Impedance measured in two electrodes before and after EO sterilization. The distribution of the channel impedance is depicted in a violin plot. Only functional channels (defined as <500 kΩ or >25 kΩ before and after sterilization) are considered.

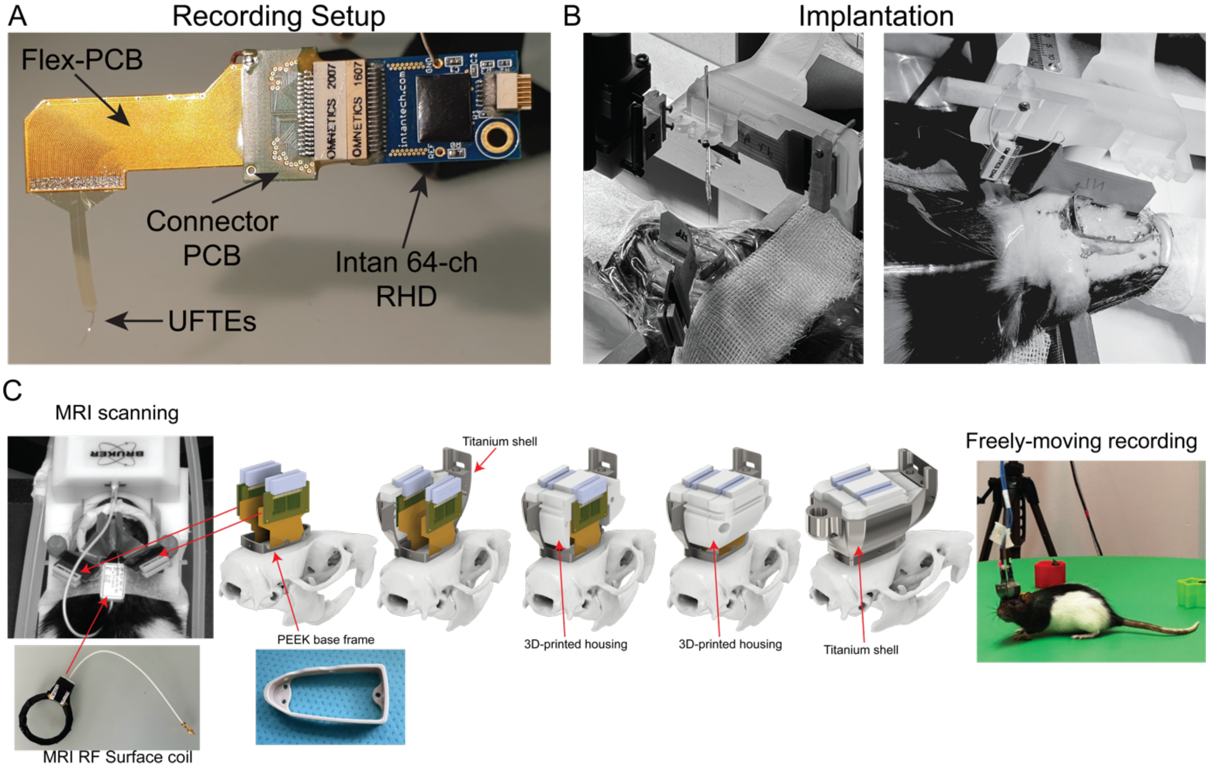

Suppl. Fig. 20. Chronic MRI-compatible recording setup. A. A sample electrode device assembled with connector PCBs and recording headstage. **B.** Implantation of UFTEs. During the implantation, flex-PCBs are cemented onto skull with their lateral anchors. **C.** Modular 3D-printed housing with titaniumHelmet for chronic MRI scanning and freely-moving awake recordings. Left-most side shows the MRI scanning setup. 30 mm surface receive-only ring coil is used. PEEK base frame is used to fixate the housing to the skull.

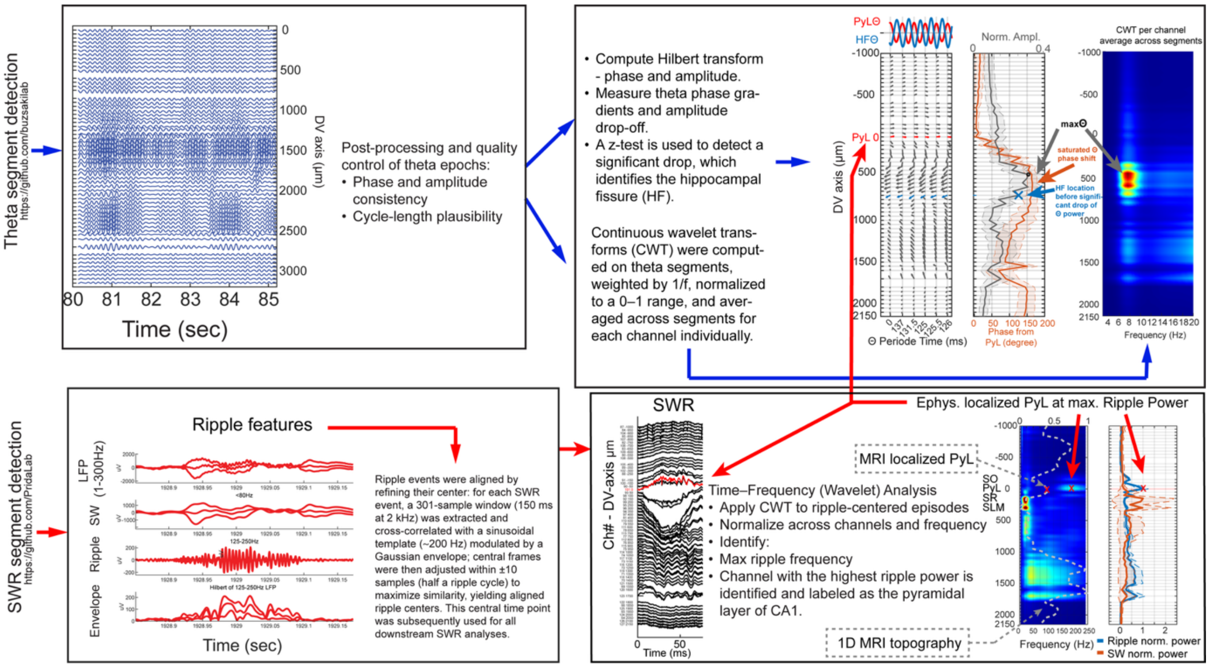

Suppl. Fig. 21. Workflow diagram illustrating electrophysiological localization of the pyramidal layer (PyL) of dorsal hippocampal CA1 and the hippocampal fissure (HF). The first row shows the workflow for HF localization, beginning with detection of the theta segment, followed by identification of the maximum theta power using the Hilbert transform of all channels along the dorso–ventral axis (grey arrow in the right panel, maximum theta in SLM). From this point, the algorithm iteratively determines the significant drop in theta power (blue arrow) together with the saturation of the theta phase shift (orange arrow) relative to the cycle zero (peak) of theta, recorded from the PyL. The second row illustrates the localization of the CA1 PyL in the dorsal hippocampus based on SWR events. The algorithm searches for the maximum ripple power along the dorso–ventral axis; once identified, the corresponding channel ID is passed to the HF detection algorithm to be used as the reference channel (0 µm) for phase difference calculations and as the starting point for detecting the drop in theta amplitude toward the dorsal direction from the PyL. Dashed grey frames and arrows indicate the corresponding points of MRI-based localization (1D MRI topography; see Methods), obtained independently by the MRI localization algorithm. Dashed grey frames and arrows indicate the corresponding points of MRI-based localization (1D MRI topography; see Methods), obtained independently by the MRI localization algorithm. Note that HF MRI localization is not labeled in the workflow, as it comes directly from the MRI localization algorithm. Finally, the difference between MRI- and electrophysiology-based localization (HF and PyL) along the dorso–ventral axis is calculated to assess accuracy (see Results).

Abbreviations: SWR – Sharp-wave Ripple, SW – Sharp-wave component of SWR (~20Hz), SO – stratum oriens; PyL – pyramidal layer; SR – stratum radiatum; SLM – stratum lacunosum-moleculare; HF – hippocampal fissure.

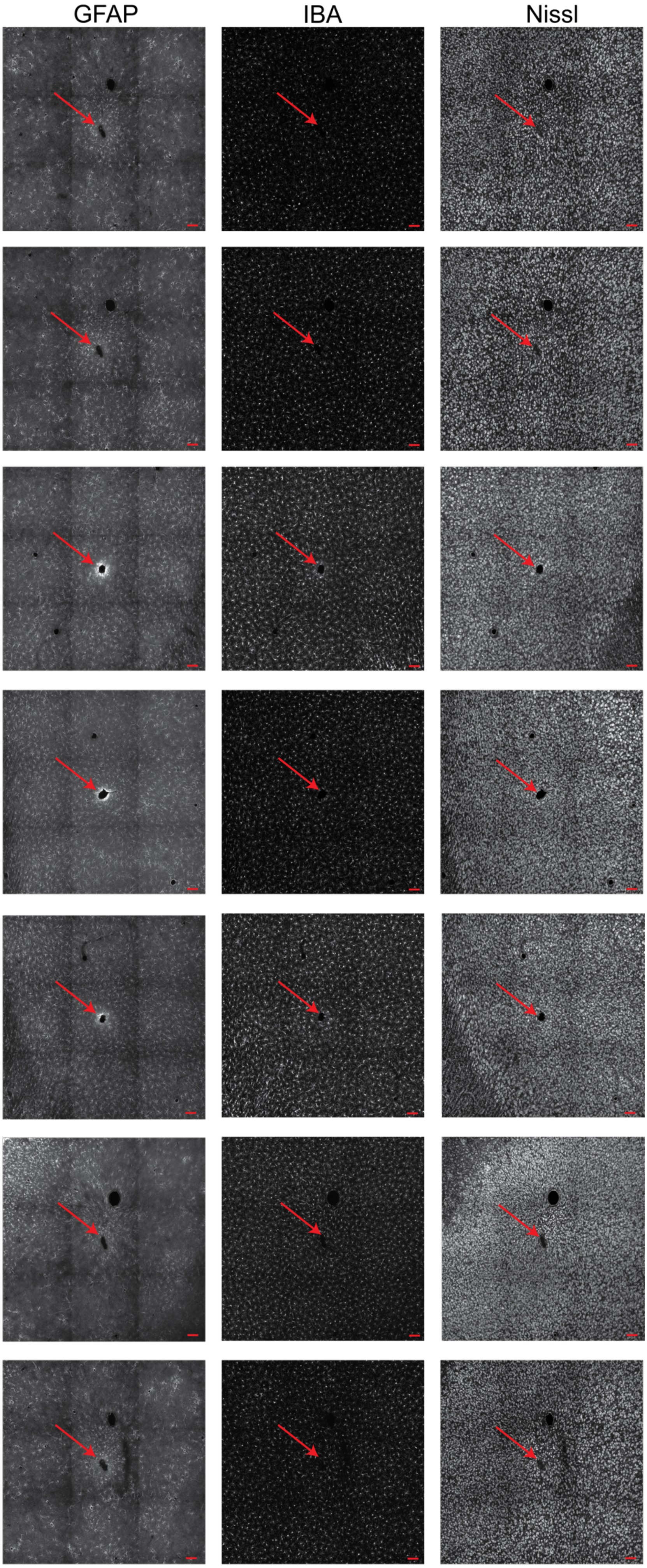
GFAP
IBA
Nissl

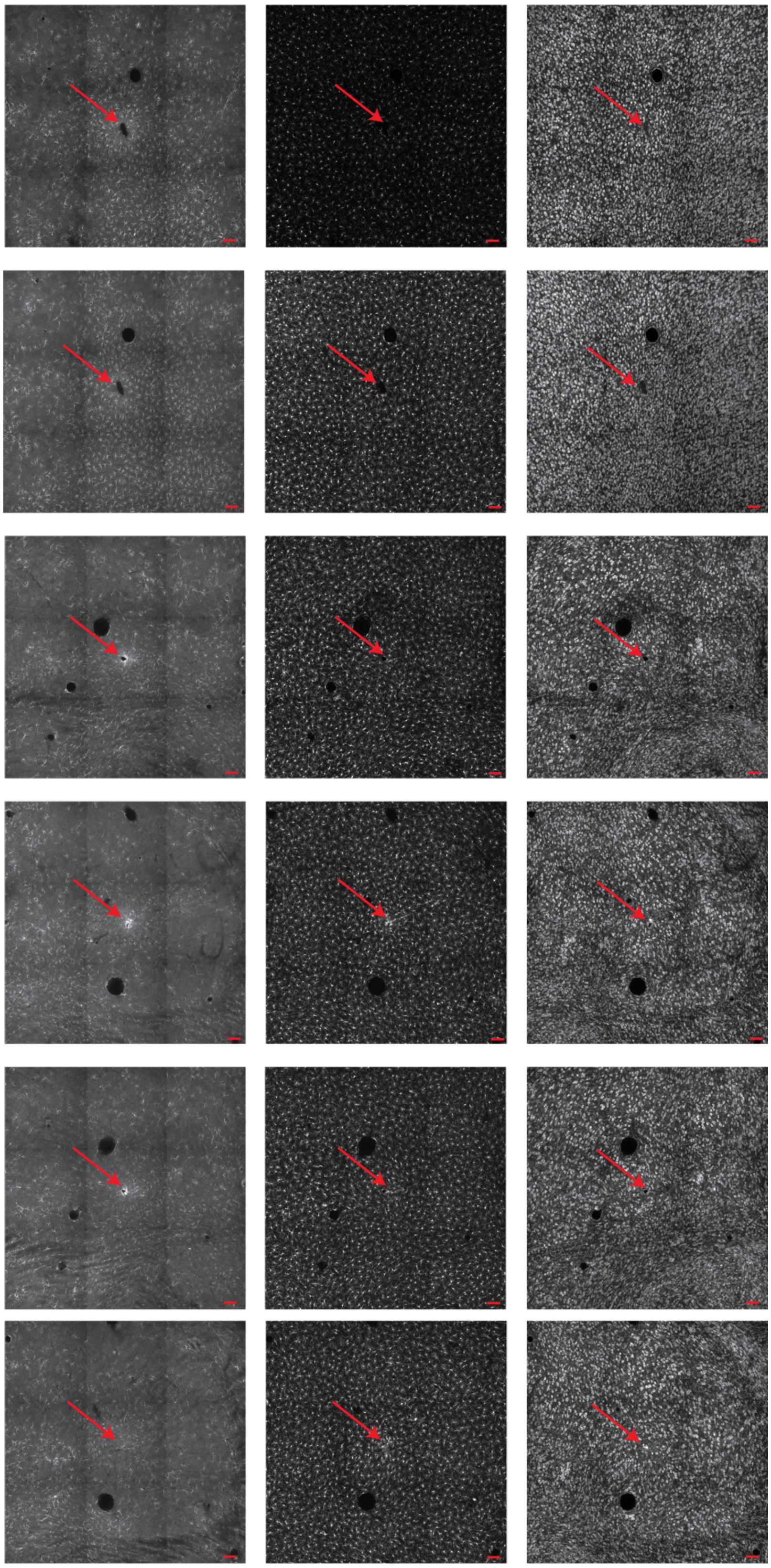

Suppl. Fig. 22. All histology images with split imaging channels (Nissl, IBA, GFAP) are shown. Red arrows point to the UFTE bundle positions. Scale bars denote 50 µm.

Supp. Video - Theta oscillations across hippocampal layers during exploratory behavior.

The opening title frame corresponds to the concept illustrated in Figure 1. The video is organized into three columns:

The left column shows the filtered local field potential (LFP) signals in the 4–10 Hz band, corresponding to theta oscillations. The x-axis represents time (s), while the y-axis represents the dorsoventral axis (μm) relative to the pyramidal layer in the dorsal hippocampus, showing theta activity across all active recording channels along the dorsoventral depth.

The middle column displays the theta phase progression across channels, referenced to the theta peak detected in the pyramidal layer. The x-axis represents one theta period time (sec) centered on the theta peak detected in the pyramidal layer, and the y-axis represents the dorsoventral axis (μm) relative to the pyramidal layer. This visualization illustrates the phase relationships across hippocampal layers during theta cycles.

The right column presents the synchronized behavioral video, allowing simultaneous observation of the rat's behavior. Theta oscillations are prominent during exploratory behavior and are interrupted during grooming (as marked in the legends below the video). Black vertical lines in the left and middle columns indicate the time points corresponding to the displayed video frame.

Abbreviations, color of text: M1, motor cortex (orange); CC, corpus callosum (green); Dorsal CA1 (cornu ammonis 1 - black) hippocampal layers: stratum oriens (Or - pink), stratum pyramidale (PyL - red), stratum radiatum (Rad - blue), stratum lacunosum-moleculare (LM - cyan); DG, dentate gyrus (brown). Thl, Thalamus (dark pink)
