# Supplementary material for: Magnetic resonance identification tags for ultra-flexible electrodes
Source: Nat Commun. 2026 Apr 28;17:5725. doi: 10.1038/s41467-026-71887-x (PMC13324162; doi:10.1038/s41467-026-71887-x)
Supplement: Supplementary file 2 — Description of Additional Supplementary Information [file 41467_2026_71887_MOESM2_ESM.pdf]

Description of Additional Supplementary Files

File Name: Supplementary Video 1
Description: **Theta oscillations across hippocampal layers during exploratory behavior**. The opening title frame corresponds to the concept illustrated in Figure 1. The video is organized into three columns:

The left column shows the filtered local field potential (LFP) signals in the 4–10 Hz band, corresponding to theta oscillations. The x-axis represents time (s), while the y-axis represents the dorsoventral axis (µm) relative to the pyramidal layer in the dorsal hippocampus, showing theta activity across all active recording channels along the dorsoventral depth.

The middle column displays the theta phase progression across channels, referenced to the theta peak detected in the pyramidal layer. The x-axis represents one theta period time (sec) centered on the theta peak detected in the pyramidal layer, and the y-axis represents the dorsoventral axis (µm) relative to the pyramidal layer. This visualization illustrates the phase relationships across hippocampal layers during theta cycles.

The right column presents the synchronized behavioral video, allowing simultaneous observation of the rat's behavior. Theta oscillations are prominent during exploratory behavior and are interrupted during grooming (as marked in the legends below the video). Black vertical lines in the left and middle columns indicate the time points corresponding to the displayed video frame.

Abbreviations, color of text: M1, motor cortex (orange); CC, corpus callosum (green); Dorsal CA1 (cornu ammonis 1 - black) hippocampal layers: stratum oriens (Or - pink), stratum pyramidale (PyL - red), stratum radiatum (Rad - blue), stratum lacunosum-moleculare (LM - cyan); DG, dentate gyrus (brown). Thl, Thalamus (dark pink)”.
