# Supplementary material for: Magnetic resonance identification tags for ultra-flexible electrodes
Source: Nat Commun. 2026 Apr 28;17:5725. doi: 10.1038/s41467-026-71887-x (PMC13324162; doi:10.1038/s41467-026-71887-x)
Supplement: Supplementary file 4 — Reporting Summary [file 41467_2026_71887_MOESM4_ESM.pdf]

nature portfolio

Corresponding author(s): Mehmet Fatih Yanik

Last updated by author(s): 10.03.2026

Reporting Summary

Nature Portfolio wishes to improve the reproducibility of the work that we publish. This form provides structure for consistency and transparency in reporting. For further information on Nature Portfolio policies, see our Editorial Policies and the Editorial Policy Checklist.

Statistics

For all statistical analyses, confirm that the following items are present in the figure legend, table legend, main text, or Methods section.

n/a	Confirmed	
☐	☒	The exact sample size (*n*) for each experimental group/condition, given as a discrete number and unit of measurement
☐	☒	A statement on whether measurements were taken from distinct samples or whether the same sample was measured repeatedly
☐	☒	The statistical test(s) used AND whether they are one- or two-sided<br>*Only common tests should be described solely by name; describe more complex techniques in the Methods section.*
☒	☐	A description of all covariates tested
☒	☐	A description of any assumptions or corrections, such as tests of normality and adjustment for multiple comparisons
☐	☒	A full description of the statistical parameters including central tendency (e.g. means) or other basic estimates (e.g. regression coefficient) AND variation (e.g. standard deviation) or associated estimates of uncertainty (e.g. confidence intervals)
☐	☒	For null hypothesis testing, the test statistic (e.g. *F*, *t*, *r*) with confidence intervals, effect sizes, degrees of freedom and *P* value noted<br>*Give P values as exact values whenever suitable.*
☒	☐	For Bayesian analysis, information on the choice of priors and Markov chain Monte Carlo settings
☒	☐	For hierarchical and complex designs, identification of the appropriate level for tests and full reporting of outcomes
☐	☒	Estimates of effect sizes (e.g. Cohen's *d*, Pearson's *r*), indicating how they were calculated

Our web collection on statistics for biologists contains articles on many of the points above.

Software and code

Policy information about availability of computer code

Data collection	Bruker 7T PharmaScan 70/16, Intan RHX 3.1 (Intan Technologies), Olympus IXplore Spin
Data analysis	Matlab (MathWorks), Python 3, SAMRI, ANTs, ITK-SNAP, ImageJ (NIH), cellSens (Olympus LS), JRCLUST 4.0.0,<br>Custom code will be provided in a github repository.

For manuscripts utilizing custom algorithms or software that are central to the research but not yet described in published literature, software must be made available to editors and reviewers. We strongly encourage code deposition in a community repository (e.g. GitHub). See the Nature Portfolio guidelines for submitting code & software for further information.

Data

Policy information about availability of data

All manuscripts must include a data availability statement. This statement should provide the following information, where applicable:

- Accession codes, unique identifiers, or web links for publicly available datasets
- A description of any restrictions on data availability
- For clinical datasets or third party data, please ensure that the statement adheres to our policy

All data supporting the findings of this study are available within the article and its supplementary files. Additional data will be made available. Any additional requests for information can be directed to, and will be fulfilled by, the corresponding authors. Source data are provided with this paper.

Research involving human participants, their data, or biological material

Policy information about studies with human participants or human data. See also policy information about sex, gender (identity/presentation), and sexual orientation and race, ethnicity and racism.

Reporting on sex and gender	n/a
Reporting on race, ethnicity, or other socially relevant groupings	n/a
Population characteristics	n/a
Recruitment	n/a
Ethics oversight	n/a

Note that full information on the approval of the study protocol must also be provided in the manuscript.

Field-specific reporting

Please select the one below that is the best fit for your research. If you are not sure, read the appropriate sections before making your selection.

[x] Life sciences [ ] Behavioural & social sciences [ ] Ecological, evolutionary & environmental sciences

For a reference copy of the document with all sections, see nature.com/documents/nr-reporting-summary-flat.pdf

Life sciences study design

All studies must disclose on these points even when the disclosure is negative.

Sample size	Number of MRID-tagged electrode shanks implanted n=11 in seven rats. n=3 MRID-tagged electrode shanks had active electronics connected for chronic electrophysiology recordings. n=8 MRID-tagged electrode shanks implanted without active circuitry solely for MRID-tag induced MRI signal characterization.
Data exclusions	Immunostaining brain slices where the tissue around electrode shank is not homogeneous due to differences of brain structures (e.g. electrode shank is located in between cortical and white matter) were excluded from the histology analysis.
Replication	n=11 MRID-tagged electrode shanks implanted in seven rats, and each MRID-tagged electrode shank imaged at multiple timepoints throughout implantation durations to ensure the reproducibility. For electrophysiological validation, we analyzed three bundles. For Bundles 1 and 2, six recording sessions were analyzed over a six-month period, while for Bundle 3, three sessions were analyzed across the same six-month interval.
Randomization	This was not relevant to our study due to the absence of multiple conditions/groups to compare.
Blinding	The contributing authors in charge of MRID-tag based electrode localization analysis were blinded to the electrophysiological analysis. The contributing authors in charge of electrophysiological electrode localization analysis were blinded to the MRID-tag based electrode localization analysis.<br>The contributing authors in charge of spike sorting, sharp-wave ripple detection, theta oscillation segment detection, and manual curation processes were blinded to analysis regarding electrode localization with electrophysiological landmarks detection.<br>The contributing authors in charge of manual MRI image segmentation were blinded to MRID-tag based electrode localization analysis.

Reporting for specific materials, systems and methods

We require information from authors about some types of materials, experimental systems and methods used in many studies. Here, indicate whether each material, system or method listed is relevant to your study. If you are not sure if a list item applies to your research, read the appropriate section before selecting a response.

Materials & experimental systems

n/a	Involved in the study	
[ ]	[x]	Antibodies
[x]	[ ]	Eukaryotic cell lines
[x]	[ ]	Palaeontology and archaeology
[ ]	[x]	Animals and other organisms
[x]	[ ]	Clinical data
[x]	[ ]	Dual use research of concern
[x]	[ ]	Plants

Methods

n/a	Involved in the study	
[x]	[ ]	ChIP-seq
[x]	[ ]	Flow cytometry
[ ]	[x]	MRI-based neuroimaging

Antibodies

Antibodies used	Rabbit anti-IBA1 (019-19741, FUJIFILM Wako), Chicken anti-GFAP (PA1-10004, Thermo Fisher), Neurotrace 530/615 Red Fluorescent Nissl Stain (N21482, Thermo Fisher) or Neurotrace 640/660 Fluorescent Nissl Stain (N21483, Thermo Fisher). Donkey anti-Rabbit Alexa Fluor 488 (A48283, Thermo Fisher) or Goat anti-Rabbit Alexa Fluor 555 plus Fab2 (A48283, Thermo Fisher). Goat anti-Chicken Alexa Fluor 647 (A32933, Thermo Fisher) or Goat anti-Chicken Alexa Fluor 647 (A32931, Thermo Fisher)
Validation	https://labchem-wako.fujifilm.com/europe/product/detail/W01W0101-1974.html<br>https://www.abcam.com/products/primary-antibodies/gfap-antibody-ab53554.html?productWallTab=ShowAll<br>https://www.thermofisher.com/order/catalog/product/N21483

Animals and other research organisms

Policy information about studies involving animals; ARRIVE guidelines recommended for reporting animal research, and Sex and Gender in Research

Laboratory animals	Rat: Long Evans, implanted at 3-16 months old
Wild animals	n/a
Reporting on sex	Rat: female
Field-collected samples	n/a
Ethics oversight	All experimental and surgical procedures involving animals were approved by the local veterinary authorities of Canton Zurich, Switzerland, and were carried out in accordance with the guidelines published in the European Communities Council Directives 2010/63/EU.

Note that full information on the approval of the study protocol must also be provided in the manuscript.

Plants

Seed stocks	n/a
Novel plant genotypes	n/a
Authentication	n/a

Magnetic resonance imaging

Experimental design

Design type	Structural brain MRI scans under anaesthesia
Design specifications	n/a
Behavioral performance measures	n/a

Acquisition

Imaging type(s)	structural
Field strength	7 Tesla
Sequence & imaging parameters	TurboRARE spin-echo (SE) scans (TE:33ms, TR: 2500ms, RARE factor: 8, echo spacing: 11, excitation angle: 90, refocusing-angle: 180; slice thicknesses: 800 µm, 450 µm, 550 µm; field-of-views: 35mm x 35mm, 18.4mm x 12mm, 35mm x 35mm; image dimensions: 256 x 256, 180 x 120, 256 x 256).<br>We mapped the B0 field homogeneity in the bore with B0MAP sequence (2 echo images TE0: 1.64ms and TE1: 5.45ms, flip angle: 30, TR: 20ms, image size: 64x64x64, FOV: 45mm x 45mm x 45mm). T2*Map multiple gradient echo (MGE) images (Echo images: 8, TE0: 4.00ms, echo-spacing: 4.09ms, TR: 800ms, flip angle: 50, FOV: 35mm x 35mm, image size: 256 x 256).
Area of acquisition	Whole-volume brain scan with coronal and/or sagittal and/or axial slicing. 3-/4- imaging slices sub-volume scans centered around detected MRID-tags where the center slice is aligned as much as possible to the MRID-tag.

Diffusion MRI ☐ Used ☒ Not used

Preprocessing

Preprocessing software	ITK-SNAP 3.6.0; Python 3; SAMRI; ANTs v2.3.5; FSLeyes v1.4.6; The custom code for MRID-analysis will be provided in a github repository.
Normalization	Prior to brain atlas registration, all images are biascorrected.<br>To compute the MRID-tag induced contrast intensity the anatomical region where the pixel is located as the baseline within the same imaging slice.
Normalization template	n/a
Noise and artifact removal	n/a
Volume censoring	n/a

Statistical modeling & inference

Model type and settings	n/a
Effect(s) tested	n/a

Specify type of analysis: ☐ Whole brain ☐ ROI-based ☒ Both

Anatomical location(s): Whole brain scans were taken following a localizer scan where the FOV was set starting from Cerebellum to the olfactory bulb of the rat brain.
ROI-based scans were taken according to the whole brain scans centered around the MRID-tags.

Statistic type for inference<br>(See Eklund et al. 2016)	voxel-wise
Correction	n/a

Models & analysis

n/a	Involved in the study
☒	☐ Functional and/or effective connectivity
☒	☐ Graph analysis
☒	☐ Multivariate modeling or predictive analysis
