# Supplementary material for: Magnetic resonance identification tags for ultra-flexible electrodes
Source: Nat Commun. 2026 Apr 28;17:5725. doi: 10.1038/s41467-026-71887-x (PMC13324162; doi:10.1038/s41467-026-71887-x)
Supplement: Supplementary file 5 — Transparent Peer Review file [file 41467_2026_71887_MOESM5_ESM.pdf]

nature portfolio

Peer Review File

Magnetic Resonance Identification Tags for Ultra-flexible Electrodes

Corresponding Author: Professor Mehmet Yanik

This file contains all reviewer reports in order by version, followed by all author rebuttals in order by version.

Version 0:

Reviewer comments:

Reviewer #1

(Remarks to the Author)
The authors present a novel approach to ultra-flexible electrodes, termed “magnetic resonance identification (MRID) tags”. Each electrode bundle carries a unique MRID tag with a barcode pattern that is visible in MRI, allowing for precise identification of the bundle and the anatomical localization of contacts. The manuscript reports in vivo testing of these electrodes, demonstrating their long-term functionality, MR visibility, and biocompatibility. This is a relevant and timely contribution to the field of neuromodulation, where precise anatomical localization is essential for targeted recording and stimulation, specifically for decoding network activity for BCI and closed-loop purposes.
The manuscript offers an important opportunity in the field namely, the ability to precisely localize contact positions non-invasively using MRI, which is essential for signal sorting. However, several concerns need to be addressed to strengthen the conclusions and improve the overall scientific rigor.

Comments to the authors

- The manuscript aims to demonstrate high spatial resolution of electrode contacts, by correlating the contact positions to functional signature of the anatomical structures. However, the current data do not convincingly support this claim.
The inter-contact spacing is in the range of hundreds of microns, but the functional resolution achieved in the recordings does not match the theoretical spatial resolution of the device.
The recordings were performed in M1, corpus callosum, CA1, and DG. However, these regions do not provide an optimal framework for validating spatial resolution in a functionally meaningful way.
A more suitable approach would involve targeting hippocampal CA1 laminae; for example, using a trajectory that passes through the stratum oriens, stratum pyramidale, and stratum lacunosum-moleculare.
Following stimulation of the schaffer collaterals, such a trajectory would allow detection of well-characterized laminar-specific responses (e.g., population spikes and EPSPs), with opposing polarity in dendritic (SO and SP- upward/positive EPSPs and spike) and SLM regions (downward/negative EPSPs and spike). This design would offer a classical and highly interpretable electrophysiological fingerprint that could validate both anatomical targeting and functional resolution of the electrodes.

- The authors conclude that the electrodes do not trigger a tissue reaction based on immunohistochemical staining for astroglial markers (GFAP and IBa-1). However, this conclusion is not sufficiently supported because:
No proper control condition is included for comparison. A reliable assessment of tissue reaction requires a comparative analysis with a conventional electrode or sham condition.
Furthermore, only three animals were used for in-vivo testing, and only one of them was assessed for astrogliosis! This is insufficient to draw any reliable conclusions about biocompatibility.
Unless a group-level analysis is performed with an adequate sample size and appropriate statistical power, this dataset should not be presented as definitive evidence of biocompatibility. At best, it should be framed as preliminary, qualitative data.

- The in vivo study is significantly underpowered. While three animals may be adequate for electrophysiological recordings, the sample size is insufficient for quantitative histological or MRI-based validation.
The authors should clearly acknowledge this limitation in the manuscript.

Claims related to MRI visibility and biocompatibility should be presented as qualitative observations rather than statistically supported conclusions.
The main conclusions of the paper should instead focus on electrophysiological performance, where the methodology appears more robust.

In summary, the technology introduced in this study has the potential to significantly advance the field of implantable neural interfaces by enabling MRI-based electrode localization. However, the current manuscript overstates several conclusions that are not fully supported by the data. To improve the manuscript, the authors should:
Clearly define the limitations of their histological and MRI analyses;
Focus the main claims on electrophysiological performance;
Reconsider the experimental design to more robustly validate spatial resolution.
I recommend major revisions before this manuscript can be considered for publication.

Reviewer #2

(Remarks to the Author)
The authors present the concept, development, and in vivo and chronic validation of magnetic resonance imaging (MRI)-compatible tags made from superparamagnetic materials. The MRI tags are IONP-coated structures embedded in flexible neural threads, each containing one electrode. The MRI tags generate a contrast detectable in MRI scans, which, together with the known dimensions and position of the electrodes in the original layout of the implants, allows the implementation of mathematical models to identify the anatomical position of individual electrodes in MRI scans. The accurate localization of individual electrodes was confirmed by the correlation of their position with electrophysiological landmarks of neural activity. While the use of MRI tags in this context is innovative, it is ambiguous how these tags function as barcodes, especially given their primary use as markers. Please consider revising the presentation of this concept in light of the definition of a barcode. As barcodes encode data, they are used as markers to identify positions in the context of this study. Furthermore, the authors should revise the following concerns:

General:

• Please introduce all abbreviations when they are used for the first time in the text.
• Introduction:
o The authors assume that the reader is an expert in MRI, however, this might not always be the case. Please briefly introduce MRI and the challenges of imaging state-of-the-art neural implants with MRI.
o The gap, the solution, and what is shown in the manuscript need to be synthesized better without revealing too many details of the main takeaways from the beginning. At this point, a concise introductory paragraph is needed.
• Discussing the results immediately after they are described makes it easier to read, as it avoids going back and forth between the results and discussion sections when presented separately.
• A summary/conclusion/outlook section is missing.
• Results:
o The order in which the results are presented makes it difficult for the reader to understand the concept. A suggestion would be to begin by describing the MRID tag concept and introducing the requirements for development to understand material selection, dimensions, and “barcode” design. Discuss microfabrication, followed by implementation and validation.
o To provide context for the rationale behind the ideation of the concept, it would be highly beneficial to introduce MRI abbreviations and concepts, such as “T2-weight” and “TEs” before using them in the text.
o It appears that part of the methodology used to decode the MRID-tag positions belongs in the Results section, as the reader must flip back and forth between the Results and Methods sections to understand what the authors have done. Please consider re-writing these sections to provide clarity to the reader.
o Did you check (e.g., simulate) if there is any heat transfer from the electrodes to the tissue during MR imaging? Can the front-end connectors (e.g., cables, PCBs) affect the MRI scan or pose a risk to the subject?
o The development rationale is unclear. Did the authors go directly from concept ideation to in vivo implementation? Were there any simulations carried out before? Or were there any in vitro or ex vivo/cadaveric tests carried out with brain phantoms or cadavers?
o How does the implementation of embedded IONP-coated structures affect the long-term stability (for more than six months in biological conditions) of the implants? Iron oxide is biodegradable, if there is any kind of water intrusion, there is a risk that IONP tags degrade. Were there any accelerated aging tests carried out?
o For chronic applications, and especially when considering human applications, device sterilization is of utmost importance. What sterilization method was used, or which sterilization method could be compatible with the approach presented? How can device sterilization affect long-term stability?
o More detailed discussion on individual results is missing. Results are not compared with other state-of-the-art imaging techniques used to localize electrodes, such as X-rays or CT? What about using devices with functional MRI? How does this approach compare with other approaches using MRI-compatible neural implants?
o What is the rationale for selecting IONPs over other materials (e.g., gadolinium) for the same purpose? The authors should justify and discuss material selection.
o The IONP tags are permanently implanted along with the neural threads. Are MRI scans still clear enough to distinguish the surrounding anatomical structures in the brain?
Line-by-line comments:

• Line 42-45: It reads like a random sentence. It should be mentioned as an example of flexible neural probes with a minimal footprint. It would fit better to annex the sentence at the beginning of the paragraph starting in line 60 'Here, we developed magnetic resonance identification (MRID) tags to identify UFTE bundles...'.
• Line 54-56: Can you also comment on the efforts to use markers for other imaging methods, such as X-ray?
• Line 56-58: Other pre-clinical methods might have been overlooked. For example, can you comment on the pros/cons of other imaging methods, such as fluorescent indexed barcodes mapped through confocal/multiphoton microscopy (Yang et al., Bionspired neuron-like electronics. Nat Mat, 2019)?
• Line 61-62: What are the challenges of introducing imaging tags in neural implants concerning material and imaging method? Additionally, could you first introduce MRI contrast agents? What are they used for?
• Line 64-66: These terms need to be introduced briefly before using them. What are “topography-controlled capillary” and “convective assembly”?
• Line 60-79: It reads more like part of the conclusion.
• Line 77: Please introduce the abbreviation when used for the first time in the text.
• Line 77-78: What are theta rhythms and SWRS? What are the main features that characterize these oscillations?
• Line 97: Although it was briefly introduced in the Introduction, what are IONPs? What are the requirements for their selection? For example, NP size, material biocompatibility, MRI compatibility, etc.
• Line 100: It reads as if the recording sites were connected to active electronics. Is this the case? If not, please remove the word 'active', as it implies the use of active electronics.
• Line 102-104: At this point, it's hard to get a sense of the dimensions of the coating (is it a monolayer of IONPs?) and the components of the barcode, e.g., the circular assembly, the individual dot of the dot matrix, and the barcode itself, what does it encode for. It would improve clarity if you avoided using different names for the same concept and just mentioned the exact dimensions or the range of dimensions. In addition, including labels for what a dot, or a circular assembly, and a barcode are in Figure 1A, would improve readability. Are there any constraints that limit the size of the IONP coating thickness, or dimensions?
• Line 102: It's unclear what modularly implies in this context. Please explain.
• Line 110: Please introduce the abbreviation of “dH2O”.
• Line 112-114: It would be helpful if CCA coating were introduced before, so that the method is clear. What is the difference with a 'doctor blade coating'? Are there nanoparticles left at the surface of the photoresist mask or only at the openings?
• Line 111: Can you elaborate more on the confinement slide? What is it? Fig. 2B is unclear in this regard.
• Line 117: Recessed dot-matrix topography = trap? Please be consistent along the text using the same words to avoid confusion when reading.
• Line 123: What is meant by "intermediate contact with the Au surface below (Fig. 2C)"?
• Lines 124-128: The SEM images provided do not give enough detail to observe this. please provide images with a higher magnification or with a better quality, e.g., the SEM image in Fig. 2C.
• Lines 134-137: Did you do adhesion tests of the IONP dots? Can the IONP coatings affect the adhesion of the PI sandwich?
• Line 134: As commented before, please revise the quality of the SEM inset image, Fig. 2C inset.
• Line 146: Please revise the quality of the SEM images, they look blurry.
• Lines 148-149: How do the quality of the IONP coatings differ? The quality of the SEM images is poor, therefore, it’s hard to tell if there is a difference between IONP coatings on smooth Au or on PI, as there is a clear difference with coatings on porous Au. Why do the IONPs need to be coated onto the Au surface? Could they be also encapsulated or embedded within the polyimide layer?
• Line 153: It seems that there is a misalignment of the barcode, or are these imaging artifacts? In the case of a misalignment, is this caused by the lithography of the deposition mask or by the lift-off/stripping of the photoresist? Also, what would be the effect of a misalignment on the reading accuracy of the barcode?
• Line 186: What is the exact MRI voxel size?
• Line 189: The comparison is not easy to imagine because thickness, area, and voxel are three different units. In this context, what is the bundle thickness? Isn't the thickness the same for each individual fiber? In the case where the thickness refers to the overall or cumulative thickness of all fibers, isn't it then affected by the assembly of the bundle on the shuttle together with the silk coating? How accurate are the dimensions of the coating and the spatial arrangement of the fibers forming a a bundle?
• Line 195: "T2*-weighted contrast" has not yet been introduced. Please add more context and a definition to the concept. The sample applies to "TEs".
• Line 202: Please specify that sigma is the standard deviation.
• Line 205: Are there any other patterns used? Is there a rationale behind the pattern design of the barcode? What do they encode?
• Line 207-209: Could you please give an example of the baseline taken or please rework the wording of the sentence to provide clarity.
• Line 207: In relation to Fig.3B, please provide the original images without the green annotations referring to the quad pattern detected MR image pixels in the supplementary figure or in the same figure panel.
• Line 212: Could you please briefly expand the description on the sliding window?
• Line 214: "heatmap contrast map" reads redundantly. Perhaps it sounds better referring to it as "contrast heatmap". Would it be possible to include additional information in the supplementary explaining how the heatmap is generated with sample data in between steps?
• Line 220-221: What does this mean experimentally? Would it be possible to use such tags in highly-dense electrode arrays that have more than one electrode per fiber closely packed? What are the limitations?
• Line 227: These predefined constraints need to be described from the beginning.
• Lines 231-233: It's unclear how the MRID tags allow identification of individual electrode fibers within a bundle. It appears that MRI is good enough to identify the IONP island or cluster of electrodes, but not to accurately identify the location of

individual electrodes, which is still a computational method based on the design features of the device and not the imaging method per se.

• Line 236, Fig. 3E: How do these measurements vary between samples? Is there a way to show the variability (e.g., standard deviations) between samples visually? Could you perhaps add the additional example plots as in Fig. 3B, but for different samples (bundles and animals)?
• Line 267-291: Please introduce the figures of merit used to validate the physiological landmarks of the activity recorded. What is the biological meaning of the ripple power, or the phase differences and amplitude? It's unclear how these were used to validate the electrode localizations using the MRID-tags.
• Line 270: Should it be referring to Figure 4A?
• Line 272: Could you please give a brief introduction to the 1D MRI-registered trajectory or topography? This will help people who are not familiar with MRI.
• Line 288-291: Do you refer here to the MRI- and Ephys-detected locations of the electrodes at the anatomical regions of PyL and HF? To make the sentence clearer, please add 'electrode locations' somewhere in the sentence. Additionally, how do you explain the high standard deviations? "n=3 bundles" across how many animals? or is it bundles within the same animal? Please comment.
• Line 293-294: How were these contact site locations determined? Due the electrophysiological landmarks of the activity recorded or to the MRID-tags? or both?
• Line 297-298: Figure 4E, first column. How many electrodes within the shown bundle were accurately localized? Is it one electrode per unit (spike waveform) detected?
• Line 310: Please explain what "overall classification accuracy" means, how was it defined? How was it calculated? What is the accuracy of the classification or the localization yield of the electrodes across the samples? Could you do the same analysis as in Figure 4 for another bundle in a different animal and add it to the supplementary? This would show any differences between the samples.
• Line 311-321: The paragraph reads as if a couple of bullet points were just strung together without any connection between them. Could you please rework the paragraph to make it clearer?
• 322: Could you please comment and discuss the results of the electrolytic lesions observed during post-mortem histology? What did you find out? How does it correlate with the MRI scans and prediction of electrode positions?
• Line 349-355: What is the ratio of working electrodes across time?
• Line 346: What happened to the third animal?
• Line 361-365: How many single- or multi-unit activity spikes were detected by each electrode, and how did this change over time? Did you notice any change in the position of electrodes over time? Perhaps you could provide a collage of MRI images at the same anatomical location in which a bundle is detected but at different points in time (e.g., monthly). How did the CoMs change over time?
• Line 367-369: This sentence belongs to the Methods. Please check also the wording for clarity.
• Line372-373: If there's a significant decrease in impedance, it could mean that the electrodes are failing. What was the ratio of functional electrodes over time?
• Line 376-379: It seems that this sentence belongs to the Methods, what is the main takeaway here? Why did you slice the trajectory horizontally?
• Line 386-392: If the trajectory was sliced differently, is the MRID contrast comparison between Animal#1 and Animals#2-3 equivalent? From Animal#1 it seems hard to distinguish the spatial distribution of the bundle, I would've expected to see a more enlarged or circular MRI contrast profile, could you please comment how is the imaging slicing orientation affecting the localization of the MRID-tags?
• Line 396: typo? "...to image neurons".
• Line 399-401: Could you please provide an overview of all 13 slices in the supplementary? Why is the implant red? How is the footprint (cross-sectional area) of the implant changing across depths?
• Line 404-409: How do these results compare to the literature? or to your previous publication without the MIRD-tags?
• Line 443-445: To which structure do you refer here?
• Line 445-446: In which cases?
• Line 446-447: Do you mean an intermediate layer of IONPs encapsulated with PI? Is that the IONPs would be directly on PI and not anymore on the Au traces of the electrodes? Why would it doubled?
• Line 451: Replace center of masses for CoM.
• Line 460: Please introduce the abbreviation PEEK when first used in the text.
• Line 464-468: Please discuss what is the smallest INOP-coated feature size that can generate a measurable MRI contrast. This part of the discussion should be included in the Results and Discussion section.
• Line 464-468: Please discuss what is the smallest INOP-coated feature size that can generate a measurable MRI contrast. This part of the discussion should be included in the Results and Discussion section.
• Line 483: What is the expected dissolution rate of silk fibroin? Is it expected to be longer than one month?
• Line 485-486: According to Figure 5A, impedances were stable for 53 days, decreased at day 59, were stable again until day 83, decreased again, and had a prominent decrease at day 110. However, such impedance fluctuation seemed not have affected the quality of the recordings, as SNRs even increased over time. How do you explain this? Is there any fluctuation in the electrode position? As questioned before, what was the ratio of functional electrodes?
• Line 492-494: The connection of electrode localization with such clinical applications was not made before. Can the authors elaborate more? CT is also used in those applications, is this method restricted to MRI?
• Line 492: Any electrode going inside the brain is always going to be invasive. Please reformulate the sentence.
• Line 494-496: What kind of “precisely targeted applications”? Is the localization of electrode clusters enough, or is the localization of individual electrodes needed when using imaging methods in combination with electrophysiological methods? Perhaps it was not clear, but only the localization of electrode clusters was demonstrated, and the localization limits of high-density arrays were not explored. How closely packed can the electrodes be to localize them accurately?
• Line 496-498: What is still missing so that this technology can be employed in NHP or human applications? Please

comment on, e.g., sterilization methods, front-end connectors, etc.
• Figures:
o Figure 1 should be referred to in the Results section and not directly from the introduction.
o Figure 1B: It is not representative of an implanted device in a rat brain, but rather a schematic of the insertion method; please revise its reference in line 68.
o Suppl. Fig. 1A is not referred in the text.
o Figure 2E: Is the scale bar needed? If so, what are its dimensions?
o Figure 3: Add labels to individual sections of each panel. E.g, Ai, Aii, or any other label to distinguish them.
o Figure 3: What is the difference between CoMs and curve centers? How do they relate? Please expand the explanation in the main text.
o Fig. 3A: What is $\mu 2$ or $\mu 1$, the mean of the Gaussian distribution? What is W1 and W2? Please define the variables in the figure caption and the main text.
o Suppl. Fig. 3: Please add an order to the data processing and analysis pipeline; it's overwhelming to see the schematic, and it's unclear where to start reading.
o Figure 4B: What are the units of the x axis?
o Figure. 4C: The figure caption says the first column shows raw SWR, however, it's unclear if raw means all frequencies (e.g., up to 3-5 kHz) or just the frequency range in which SWR are encountered (e.g., below 300 Hz)?
o Figure 4A and 4C, second column: Where is the color code and the corresponding units of the heatmaps?
o Figure 4: Label F is missing in the figure caption.
o Figure 4D: Is there a significant difference between the average localization difference across anatomical landmarks, e.g., PyL and HF? Is there an influence of depth on localization accuracy? Check possible typo, is it PyrL or PyL?
o Figure 4: Please add scale bars in MRI images.
o Figure 5B: It would be better to show just one animal, but with more data points over time (e.g., monthly). The data for the other animals should then be included in the supplementary. This would help to better observe how the MRI contrast is changing over time.
o Figure 5C: Please add the scale bars to the stainings.
o Suppl. Fig. 5: What is the thickness and distance between the slices? Please add scale bars and label of the different anatomical regions/layers for clarity. Such pictures should probably be better placed on the front page in the main manuscript, not in the supplementary, as they confirm the actual location of the implants.
Methods:
• Microfabrication of the ultra-flexible electrodes and MRID-tags:
o Did you explore other surface activation methods before considering roughening the Au surface with wet etching methods? For example, oxygen plasma or other surface coatings?
o Is there an adhesion promoter employed before spin-coating the second PI layer?
o Please provide details of the PEDOT:PSS blend. What is the concentration of PH100 versus EG, DBSA, and GOPS?
• Assembly of the UFTEs
o What kind of solder paste and method was used to bond the flexible implant onto the flexible PCB?
o Curing temperatures and duration of medical-grade epoxy are missing.
o Why was a two-electrode cell configuration used for impedance measurements? What equipment was used to measure impedances?
o What was the total weight of the assembly?
• Surgical procedures:
o What was the dose of the analgesic Meloxicam?
o It is mentioned that Ringer's solution and glucose were injected s.c. at fixed intervals. How often were these intervals, and what volume per weight was used?
o Did you monitor the temperature of the animal during the surgery?
o How did the animals recover? Was an additional analgesic given?
• Imaging rat brain in vivo in MRI
o Was the first scan one month post-implantation?
o What was the concentration of isoflurane used for inducing the animals?
o Did you monitor the temperature of the animal?
o Could provide pictures of the experimental setup during MRI scans. In line 631, it gives a reference to the "Supplementary", however, no reference is given, and none of the supplementary Figures is related to it.
• MRID analysis:
o Line 674: "ri denotes the relaxivity constant of the constant (where i: 1,2,2*)": Which constant? What is its value? What is i?
o Please specify that [CM] in Eq. 1 is the concentration of the contrast medium. Which units are used?
o What were the observed concentrations?
o It would give more clarity if the authors could give a full example on how the different equations were used, giving numbers to the variables in the equations, and correlating it with the images used. As mentioned before Suppl. Fig. 3 needs improvement, and the order in which the analysis is carried out is lost.
• In vivo recordings from rats
o What kind of filters were applied to the raw data? E.g., Butterworth, Notch, etc?
• Local Field Potential Data Processing
o Why were LFPs down-sampled?
o Line 821-822: What is the normalized amplitude?
• Adding diagram flows in the Supplementary to show the different offline data processing pipelines used in the electrophysiological data will give clarity to the reader.
• Electrolytical Lesioning:
o Can you provide references on this lesioning method.

o How did you confirm the lesion in vivo and post-mortem?
o Line 881: Explain what “for each polarity” means. Do you mean biphasic current pulses containing a cathodic and an anodic phase?
• Immunostaining:
o Were the rats perfused?
o Was the brain fixed with PFA or any similar?
o How was the dissection of the brain carried out? Were the implants still inside the brain during brain sectioning? Or was the implant retrieved?
o What is the “established protocol”? Please add references.
o Why did you section the brain into 100 µm-thick slices? Wouldn’t thinner slices be a better option to provide better details?
o What was the PBS concentration used? Was PBS self-made or was it acquired from a commercial supplier?
o Add details for suppliers and product numbers on all solutions, antibodies, etc.
o Are these free-floating stainings? Or directly on glass slides?
o Why was Nissl used instead of NeuN or MAP2, given that IHC was being carried out?

Version 1:

Reviewer comments:

Reviewer #1

(Remarks to the Author)
The authors have provided sufficient responses to my comments and have addressed the majority of them in the revised manuscript. Therefore, I find the revision satisfactory and recommend the manuscript for publication in Nature Communications.

Reviewer #2

(Remarks to the Author)
The revisions significantly improved the quality of the manuscript. However, a few open questions remain. Please see comments below:

• Regarding the simulations and analytical calculations carried out for the critical wire length that could induce heating: Did you correct the Larmor frequency for the surrounding tissue? 1 m and 50 cm would correspond to the wavelength and half-wavelength resonance conditions if the medium were air. Which relative permittivity did you consider in your simulations? This is an important parameter, given the complex nature of the permittivity in biological tissue, which leads to frequency-dependent values that could range between 30 to 100, depending on which source you consult. If you assume it to be 50, the half- and quarter-wavelengths are already 7 and 3.5 cm, respectively. Given that the electrodes are 6 mm long, what is the overall conductive length, including the feedlines connecting the electrodes to the pads (see also Suppl. Fig. 5. and Suppl. Fig. 20A, feedlines along polyimide implant), that may couple to RF fields and contribute to heating?
• Why were 2% agarose gel phantoms selected, instead of the typical 0.6% agarose w/w brain phantoms used to mimic the brain? What was the conductivity of the brain phantom used?
• Long-term stability: It seems that the impedance median is getting lower across months (nearly half after 6 months compared to 1-month post-implantation, see NEW Rebuttal Figure. Chronic stability example of an MRID-tagged UFTE, panel C), and it also seems that the contrast has a negative trend. Could this be an early indication of water intrusion between the layers (not necessarily delamination per se)?
• Regarding the question about possible electrode movement over time. Where can I find Suppl. Fig 6.1 and 6.2?
• According to New Suppl. Fig. “Localization differences between MRID electrode-based localization...”, localization drifts of up to 150 µm can be observed. While electrodes would still be in CA1, they could be moving across layers within the CA1. Is this a correct interpretation? According to Fig.5B, the electrodes do move.

Open Access This Peer Review File is licensed under a Creative Commons Attribution 4.0 International License, which permits use, sharing, adaptation, distribution and reproduction in any medium or format, as long as you give appropriate credit to the original author(s) and the source, provide a link to the Creative Commons license, and indicate if changes were made.
In cases where reviewers are anonymous, credit should be given to 'Anonymous Referee' and the source.
The images or other third party material in this Peer Review File are included in the article's Creative Commons license, unless indicated otherwise in a credit line to the material. If material is not included in the article's Creative Commons license and your intended use is not permitted by statutory regulation or exceeds the permitted use, you will need to obtain permission directly from the copyright holder.
To view a copy of this license, visit https://creativecommons.org/licenses/by/4.0/

RESPONSE TO REVIEWERS

We would like to thank all reviewers for very helpful and constructive feedback. Please find below our point-by-point reply to reviewers' comments. We highlighted all changes in the manuscript and supplementary information in red. We uploaded the revised manuscript text and supplementary information to the Manuscript Tracking System. In addition to the changes in Figures asked by the reviewers, we moved the Figure 1 panel B to the supplementary information to improve the readability of Introduction; we added informative texts and legends to Figure 2 panel A while adding sub-panel indices for better readability; we moved the Figure 2 panel D "IONP coating on different surfaces" SEM images to supplementary information while moving the inset SEM images from panels B and C to panel D with increased size to improve visibility; we replaced the SEM image in Figure 2 panel F part i with a more recent image with better IONP dot alignment during photolithography; we added a label in Figure 2 panel F part ii highlighting a sample dot-matrix to improve readability; we added a similar label in Figure 3 A part i to highlight dot-matrix on electrode gold tracks; we highlighted four exemplary channels in Figure 3 panel B part i with yellow rectangles to provide better explanation on their position relative to CoMs; we updated the Figure 3 panel C "MRID trajectory reconstruction accuracy data" with absolute differences and the data added from **four** new animals implanted for the revision; we moved Figure 3 panel E "MRI contrast vs IONP coating density" plot to Figure 3 panel G while adding **two new** figures for MRI-barcode analysis to Figure 3 panels E and F; we added labels to Figure 4 panel C to highlight the laminae of dHPC; we updated the Figure 4 panel D "accuracy barplots" with all data pooled from multiple chronic MRI sessions to improve the statistical analysis; we changed the spacing of illustrative contours in Figure 5 panel C from 50 µm to 40 µm to make it more consistent with statistical analysis; we updated the immunohistology fluorescence intensity plots in Figure 5 panel C solely cosmetically (mirrored vertically) for better readability. We added another example to "histology validated MRI-localization results" (Suppl. Fig. 16) to capture variance across bundles. Since the submission of our manuscript, we have successfully tested MRID-tags in **four** new animals (n=4 bundles) for the revision and **one** animal (n=2 bundles) for another ongoing experiment in our group, which we now mention.

We also uploaded each figure separately as PDF files since there were issues raised by reviewers regarding the image resolution of figures in the initial submission manuscript PDF file.

Reviewer #1 (Remarks to the Author):

The authors present a novel approach to ultra-flexible electrodes, termed "magnetic resonance identification (MRID) tags". Each electrode bundle carries a unique MRID tag with a barcode pattern that is visible in MRI, allowing for precise identification of the bundle and the anatomical localization of contacts. The manuscript reports in vivo testing of these electrodes, demonstrating their long-term functionality, MR visibility, and biocompatibility. This is a relevant and timely contribution to the field of neuromodulation, where precise anatomical localization is essential for targeted recording and stimulation, specifically for decoding network activity for BCI and closed-loop purposes.
The manuscript offers an important opportunity in the field namely, the ability to precisely localize contact positions non-invasively using MRI, which is essential for signal sorting. However, several concerns need to be addressed to strengthen the conclusions and improve the overall scientific rigor.

Comments to the authors

1) The manuscript aims to demonstrate high spatial resolution of electrode contacts, by correlating the contact positions to functional signature of the anatomical structures. However, the current data do not convincingly support this claim.
The inter-contact spacing is in the range of hundreds of microns, but the functional resolution achieved in the recordings does not match the theoretical spatial resolution of the device.

   The recordings were performed in M1, corpus callosum, CA1, and DG. However, these regions do not provide an optimal framework for validating spatial resolution in a functionally meaningful way.

   A more suitable approach would involve targeting hippocampal CA1 laminae; for example, using a trajectory that passes through the stratum oriens, stratum pyramidale, and stratum lacunosum-moleculare.

Following stimulation of the schaffer collaterals, such a trajectory would allow detection of well-characterized laminar-specific responses (e.g., population spikes and EPSPs), with opposing polarity in dendritic (SO and SP- upward/positive EPSPs and spike) and SLM regions (downward/negative EPSPs and spike). This design would offer a classical and highly interpretable electrophysiological fingerprint that could validate both anatomical targeting and functional resolution of the electrodes.

We thank the reviewer greatly for her/his very valuable comments.

We now clarified that the **inter-contact spacing is 50 µm**, not hundreds of microns. This fine spatial resolution, combined with analysis of endogenous network events such as SWR, or Theta oscillation, allows us to resolve laminar electrophysiological patterns without the need for exogenous stimulation:

We also clarified that **our recordings indeed covered the full laminar architecture of the hippocampus, including *stratum oriens, stratum pyramidale, stratum radiatum, stratum lacunosum-moleculare* of CA1,** as well as the molecular layer, granule cell layer, and hilus of the dentate gyrus (DG). While our electrode arrays span multiple other regions as well, the detailed laminar sampling of CA1 was a key design feature and aligns directly with the reviewer's suggestion. To further clarify, Figure 4 presents data specifically from the most functionally relevant laminae of the CA1 region, including stratum oriens, stratum pyramidale, stratum radiatum, and stratum lacunosum-moleculare. We have now also **updated figure 4 to indicate the inferred CA1 laminar organization**, based on the electrophysiological markers (explained below in further detail). This provides a clear and interpretable reference for the functional anatomy represented in our recordings.SWRs are generated through coordinated excitatory input from CA3 pyramidal neurons via the Schaffer collaterals, which target dendrites in the stratum radiatum of CA1, producing a large negative LFP deflection at that layer. This current sink is accompanied by a polarity inversion in adjacent layers, including a positive (upward) deflection in stratum oriens, and by high-frequency ripple oscillations (150–250 Hz) centered in the stratum pyramidale, where CA1 pyramidal somata are located. This well-established laminar activation pattern, observed in both evoked and spontaneous manner, provides a robust and physiologically grounded framework for localizing hippocampal layers. [1–5]

To further clarify, in our recordings, the upward and downward LFP deflections associated with SWRs are directly visible in the broadband SWR (see Figure 4C, first column), reflecting the laminar polarity inversion across CA1. The negative peak in the stratum radiatum and the positive deflection in the stratum oriens confirm the expected current flow direction during the sharp wave component.

In parallel, we used averaged wavelet decomposition of all SWR to analyze the spatial distribution of spectral power across layers (see Figure 4C, second column). This analysis revealed that the maximum power of the sharp wave localizes to the stratum radiatum, while the maximum ripple power occurs within the stratum pyramidale, where the sharp wave component is absent. This dissociation between sharp wave and ripple localization provides additional evidence of laminar resolution and validates our identification of hippocampal layers based on spontaneous SWR.

This layer-specific distribution is fully consistent with canonical hippocampal physiology and confirms that our recordings resolve functionally distinct laminae.

Regarding anatomical labeling, we note that the MRI-based registration (Waxholm Space Atlas) defines anatomical regions such as CA1 but does not distinguish individual layers. However, our electrophysiological data provide finer spatial resolution, enabling functional identification of stratum oriens, pyramidale, and radiatum based on established SWR features.

We have now modified the Introduction and added an introductory section to the Results to clarify the rationale and goals of our approach.

1. Buzsáki, G. Hippocampal sharp wave-ripple: A cognitive biomarker for episodic memory and planning. *Hippocampus* **25**, 1073–1188 (2015).

2. Buzsáki, G. Theta Oscillations in the Hippocampus. *Neuron* **33**, 325–340 (2002).

3. Lopes-dos-Santos, V., Brizee, D. & Dupret, D. Spatio-temporal organization of network activity patterns in the hippocampus. *Cell Rep.* **44**, 115808 (2025).

4. Santiago, R. M. M. *et al.* Waveform-based classification of dentate spikes. *Sci. Rep.* **14**, 2989 (2024).

5. Navas-Olive, A., Amaducci, R., Jurado-Parras, M.-T., Sebastian, E. R. & De La Prida, L. M. Deep learning-based feature extraction for prediction and interpretation of sharp-wave ripples in the rodent hippocampus. *eLife* **11**, e77772 (2022).

2) The authors conclude that the electrodes do not trigger a tissue reaction based on immunohistochemical staining for astroglial markers (GFAP and IBa-1). However, this conclusion is not sufficiently supported because:
No proper control condition is included for comparison. A reliable assessment of tissue reaction requires a comparative analysis with a conventional electrode or sham condition. Furthermore, only three animals were used for in-vivo testing, and only one of them was assessed for astrogliosis! This is insufficient to draw any reliable conclusions about biocompatibility.
Unless a group-level analysis is performed with an adequate sample size and appropriate statistical power, this dataset should not be presented as definitive evidence of biocompatibility. At best, it should be framed as preliminary, qualitative data.

We indeed used only 1 animal, and our analysis of UFTEs' biocompatibility in this manuscript, indeed, could only be stated as preliminary and qualitative in that regard. Because we extensively studied the biocompatibility of UFTEs in our previous paper (Yasar et al. 2024), we now referred to our earlier work. In the manuscript, we also now acknowledge the biocompatibility results as preliminary and qualitative data which addresses the variance only across multiple bundles in one animal but not across multiple animals/subjects.

3) The in vivo study is significantly underpowered. While three animals may be adequate for electrophysiological recordings, the sample size is insufficient for quantitative histological or MRI-based validation.
The authors should clearly acknowledge this limitation in the manuscript.
Claims related to MRI visibility and biocompatibility should be presented as qualitative observations rather than statistically supported conclusions.
The main conclusions of the paper should instead focus on electrophysiological performance, where the methodology appears more robust.

For the MRI visibility analysis, we had used n=7 MRID-tagged electrode bundles implanted in n=3 animals for (3.5 months in one animal, and 5 months in the other two) where we address and explain the variance both across multiple MRID-tagged electrode bundles and multiple animals chronically.

We now implanted and analyzed another 4 animals (**bringing total to n=7 animals and n=11 shanks**). Our new findings are consistent with our previous findings. Please find below the MRI visibility data from ~1.5-months chronic implantations of 4 MRID-tagged electrode bundles into 4 animals.

We now also included these in the supplementary materials. We have also revised the MRID-tag MRI contrast characterization analysis with four extra animals in the main manuscript (Figure 3C and 3G).

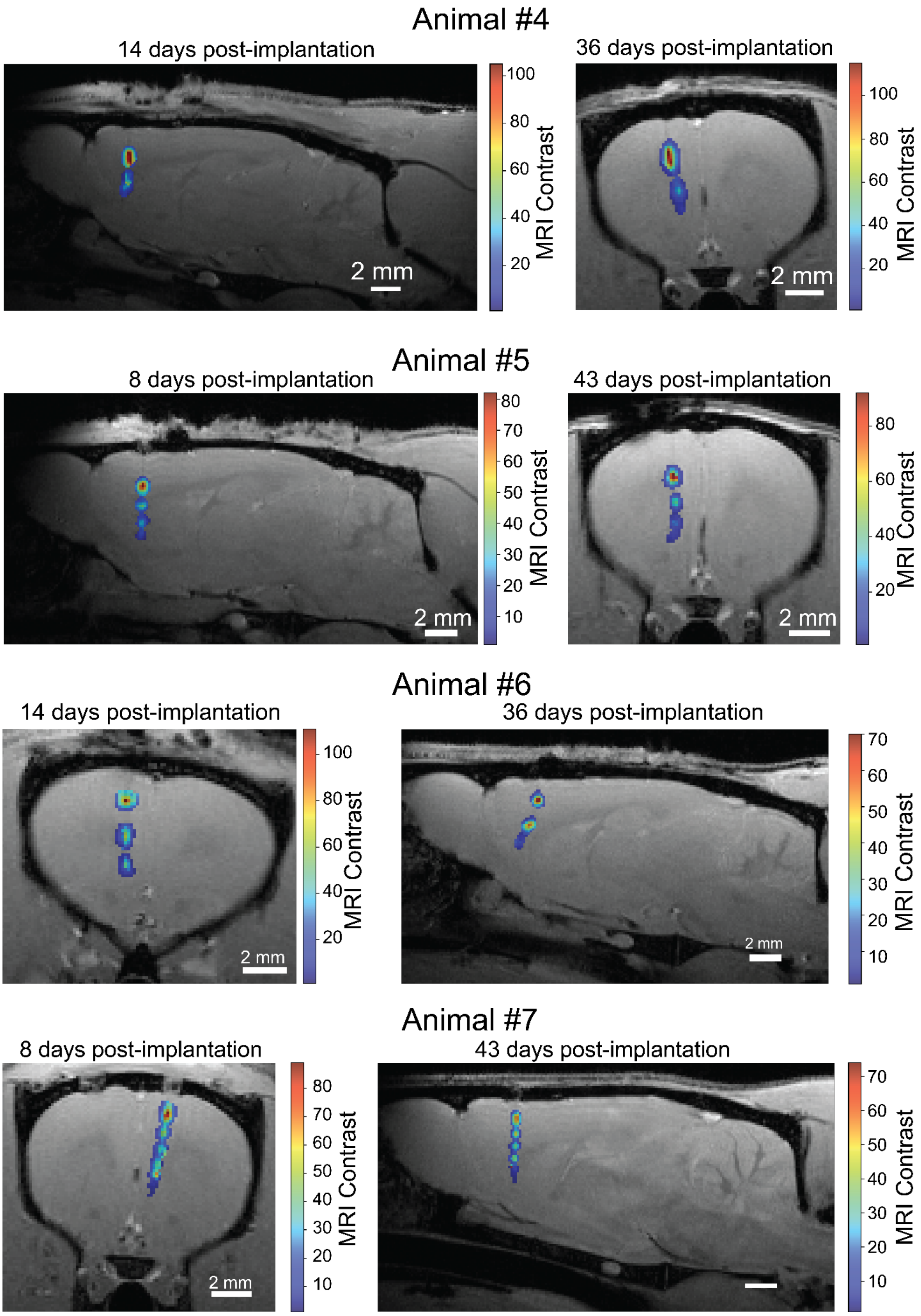

NEW Suppl. Fig. Four examples for chronically implanted MRID-tagged UFTE bundles to show the contrast heatmaps across animals, bundles and time.

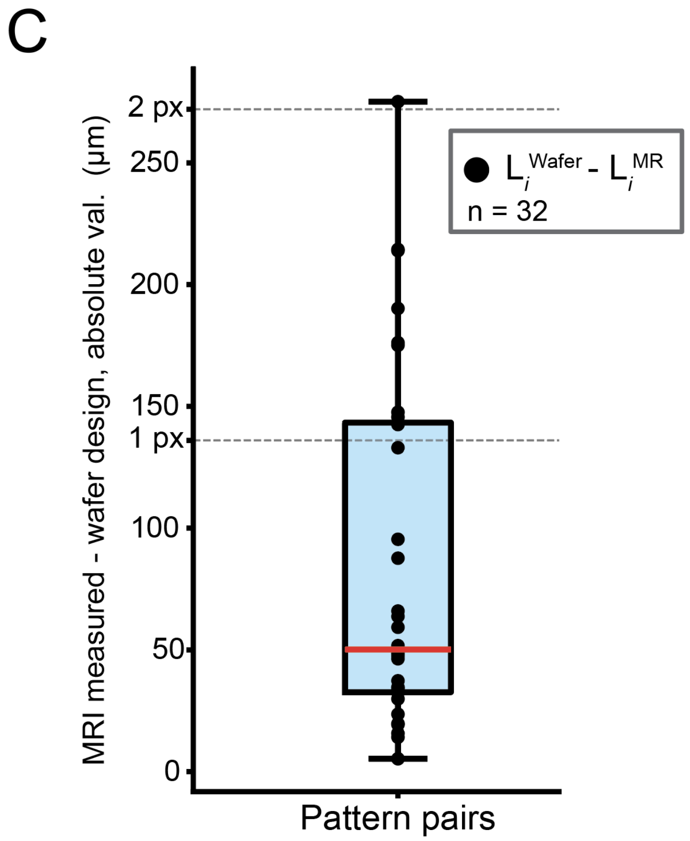

REVISED Figure 3 C. Accuracy of reconstructed bundles. Each dot represents the absolute length difference (µm) between consecutive CoMs.

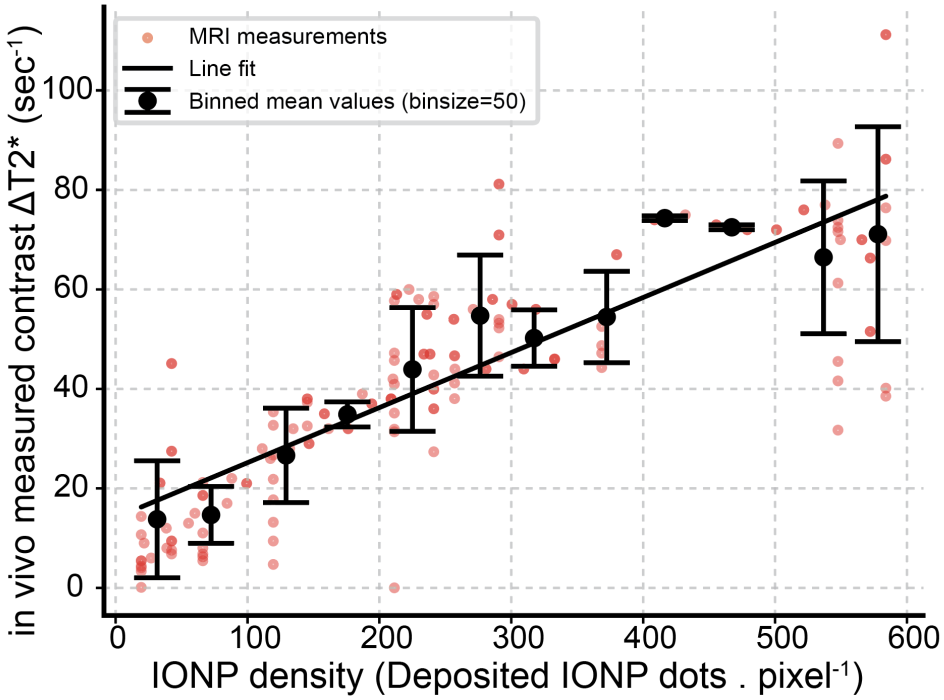

REVISED Figure 3.G. *in vivo* measured MRI contrast levels as a function of IONP coating density showing the linear correlation with best-fit line (**black**). The data from four new animals are added into the figure.

In summary, the technology introduced in this study has the potential to significantly advance the field of implantable neural interfaces by enabling MRI-based electrode localization. However, the current manuscript overstates several conclusions that are not fully supported by the data. To improve the manuscript, the authors should:
Clearly define the limitations of their histological and MRI analyses;
Focus the main claims on electrophysiological performance;
Reconsider the experimental design to more robustly validate spatial resolution.
I recommend major revisions before this manuscript can be considered for publication.

We greatly thank the reviewer for the very detailed and instructive comments and suggestions.

Reviewer #2 (Remarks to the Author):

The authors present the concept, development, and in vivo and chronic validation of magnetic resonance imaging (MRI)-compatible tags made from superparamagnetic materials. The MRI tags are IONP-coated structures embedded in flexible neural threads, each containing one electrode. The MRI tags generate a contrast detectable in MRI scans, which, together with the known dimensions and position of the electrodes in the original layout of the implants, allows the implementation of mathematical models to identify the anatomical position of individual electrodes in MRI scans. The accurate localization of individual electrodes was confirmed by the correlation of their position with electrophysiological landmarks of neural activity. While the use of MRI tags in this context is innovative, it is ambiguous how these tags function as barcodes, especially given their primary use as markers. Please consider revising the presentation of this concept in light of the definition of a barcode. As barcodes encode data, they are used as markers to identify positions in the context of this study. Furthermore, the authors should revise the following concerns:

We thank the reviewer very much for her/his insightful and highly detailed comments.

The **MRID barcodes are unique to each implanted electrode bundle**. Thus, they allow **unambiguous identification and differentiation of individual electrode bundles from others when multiple electrode bundles are implanted**. This is especially useful when the bundles are implanted densely in close proximity (e.g. Neuralink's threads). Please see below, where we tagged and identified bundles implanted within 300um of each other. Electrophysiologically alone, it is not possible to differentiate UFTE bundles in such an anatomical region (i.e. mPFC). Thus, to validate that the MRI barcodes are not simply false-positive matches to expected barcodes, we compared the MRI-barcodes to the reference barcodes from design layout and calculated the similarity scores. Using the similarity scores, we calculated the probability of MRI-barcodes belonging to each reference barcode category using the softmax function. We assigned the MRI-barcode to a reference category with highest probability. Once an electrode bundle's barcode is used to identify the bundle, we use the individual bars in the barcode as markers to accurately localize the individual electrode channels.

We have now added further clarification to the abstract and introduction. We have now also extended the Results chapter regarding MRI-barcodes analyses.

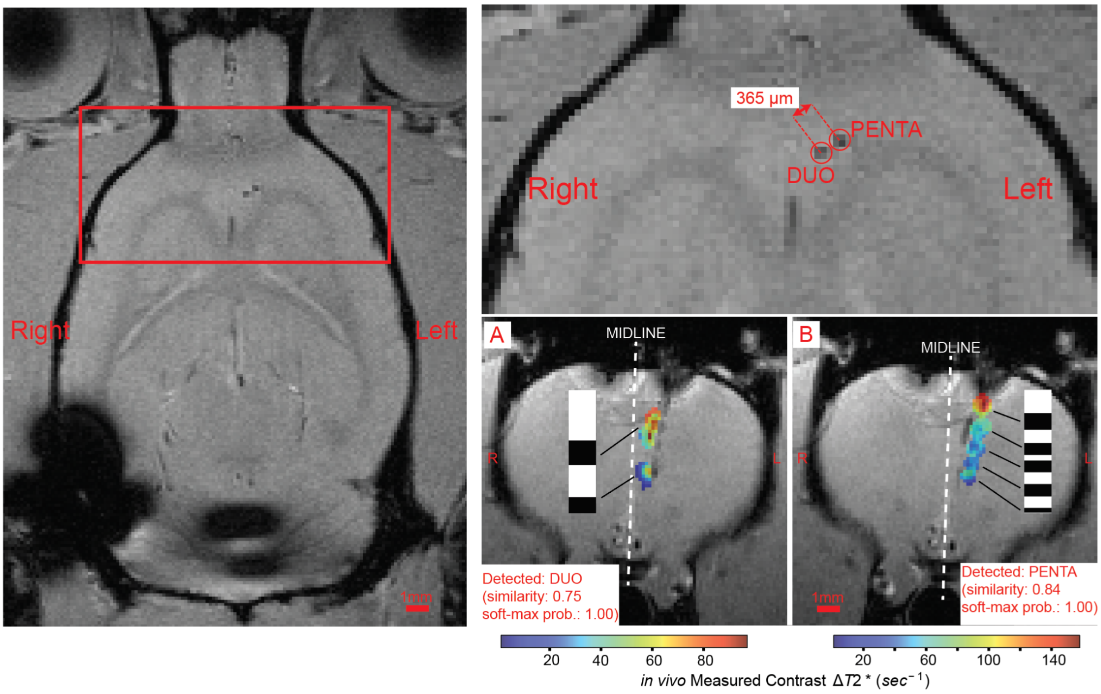

NEW Rebuttal Figure. Two MRID-tagged UFTE bundles implanted in close vicinity (365 um separation). The left panel shows the horizontal MRI image, and the top-right panel shows the zoomed-in image of UFTE bundle cross-sections. Panels A and B show the MRI-barcodes from each bundle. Insets in panels show the detected barcodes with similarity and probability scores.

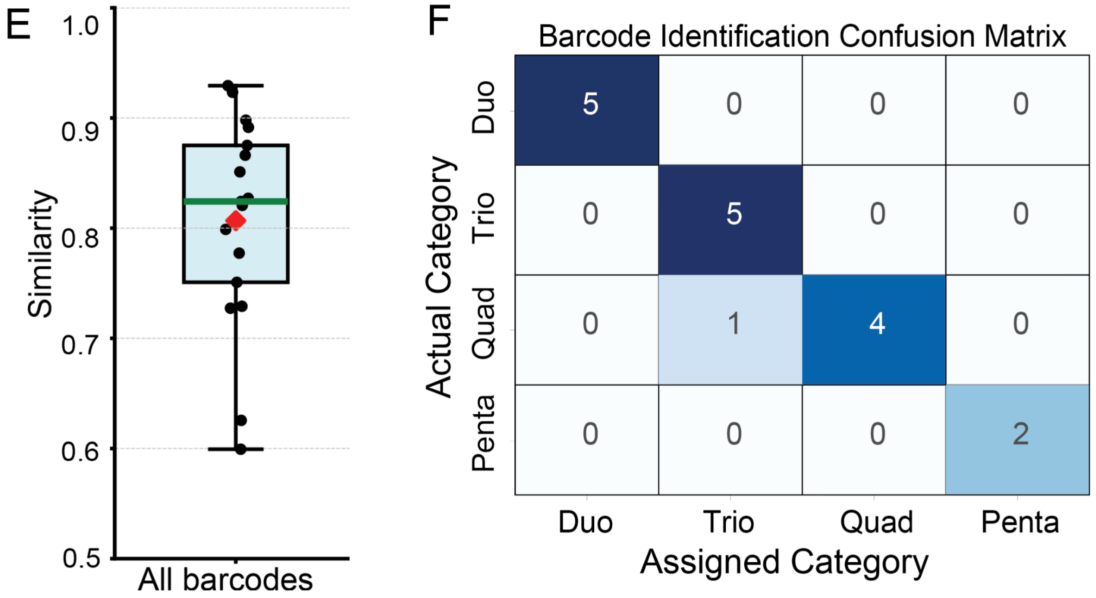

NEW REVISED Figure 3 E and F. E. Boxplot for similarity scores of reconstructed MRI-barcodes, **green** line denotes the median score, **red** diamond denotes the mean score. Data points jittered along the x-axis for better visualization (n = 17 MRI scans pooled from n = 5 animals and n = 7 UFTE bundles) **F.** Confusion matrix for MRI-barcode assignments. 94.11% of MRI-barcodes were assigned to the correct category.

General:

- Please introduce all abbreviations when they are used for the first time in the text.
  We have revised the Introduction and Results sections to define all relevant abbreviations and concepts. We also repeated the definition of abbreviations in the figures to help with readability.

- **Introduction**:

- The authors assume that the reader is an expert in MRI, however, this might not always be the case. Please briefly introduce MRI and the challenges of imaging state-of-the-art neural implants with MRI.
  We have now extended the description on MRI physics and the challenges in imaging implants both in the introduction and also in the discussion.

- The gap, the solution, and what is shown in the manuscript need to be synthesized better without revealing too many details of the main takeaways from the beginning. At this point, a concise introductory paragraph is needed.
  We now have reworked the introduction addressing the gap/problem and solution better.

- Discussing the results immediately after they are described makes it easier to read, as it avoids going back and forth between the results and discussion sections when presented separately.
  We have now reworked and discussed the results in their subsections.

- A summary/conclusion/outlook section is missing.
  We have now removed the extensive details to results' section.

  We have now reworked a conclusion/summary section.

- **Results**:
  - The order in which the results are presented makes it difficult for the reader to understand the concept. A suggestion would be to begin by describing the MRID tag concept and introducing the requirements for development to understand material selection, dimensions, and "barcode" design. Discuss microfabrication, followed by implementation and validation.
    We thank the reviewer again for this very helpful advice. We have now broken the paragraphs and reordered them as he/she suggested.

  - To provide context for the rationale behind the ideation of the concept, it would be highly beneficial to introduce MRI abbreviations and concepts, such as "T2-weight" and "TEs" before using them in the text.
    We have now revised the Introduction and Results sections to define all relevant abbreviations and concepts -including *T2-weighted imaging* (which refers to MRI contrast generated by differences in transverse relaxation time), and *TE* (echo time, the interval between the application of the RF pulse and the peak of the echo signal). These terms are now clearly introduced before their first use to ensure clarity for a broader audience.

  - It appears that part of the methodology used to decode the MRID-tag positions belongs in the Results section, as the reader must flip back and forth between the Results and Methods sections to understand what the authors have done. Please consider re-writing these sections to provide clarity to the reader.
    (We apologize that this problem arose due to the direct transfer of our manuscript from Nature Neuroscience (with strict word limits) to Nature Communications.)

    We have now expanded the results section carefully with the critical details that were previously covered only in the Methods section.

  - Did you check (e.g., simulate) if there is any heat transfer from the electrodes to the tissue during MR imaging? Can the front-end connectors (e.g., cables, PCBs) affect the MRI scan or pose a risk to the subject?

    We positioned and oriented front-end connectors avoiding formation of loop structures etc. to prevent induced Eddy currents. All front-end components can

be adjusted according to the subject and the MRI scanning configuration, and they are not in direct contact with the brain.

Our electrodes do not have large transverse surfaces that can induce Eddy currents in MRI gradients. Possible heating could occur due to RF antenna effect due to the geometry of the devices. We had carried out back of the envelope calculations considering the field strength of the scanner, Larmor frequency, our device geometries (as detailed below). In addition, we now performed full-wave COMSOL simulations to further confirm our findings

The following equation defines Larmor frequency ω:
$\omega = \gamma B$
Using B=7T and γ = 42.58 MHz/T for H1 gives ω = 298.06 MHz

For any wire to resonate and heat up under the RF field, the length of the wire should match the wavelength (λ) of the RF field by ½ λ (Konings et al. 2000).
λ=1m for ω = 298.06 MHz

Thus, the electrodes should be around 50cm long to resonate and heat up in the 7T scanner.
Since the electrodes we used in this work are 6mm long, such effects become negligible

We further did full-wave COMSOL simulations with a human-head tissue model where we observe no additional electrode-induced heating (max. +0.08°C).

We have now included the full-wave COMSOL simulation results in the supplementary materials. Please also see below the figure for your convenience.

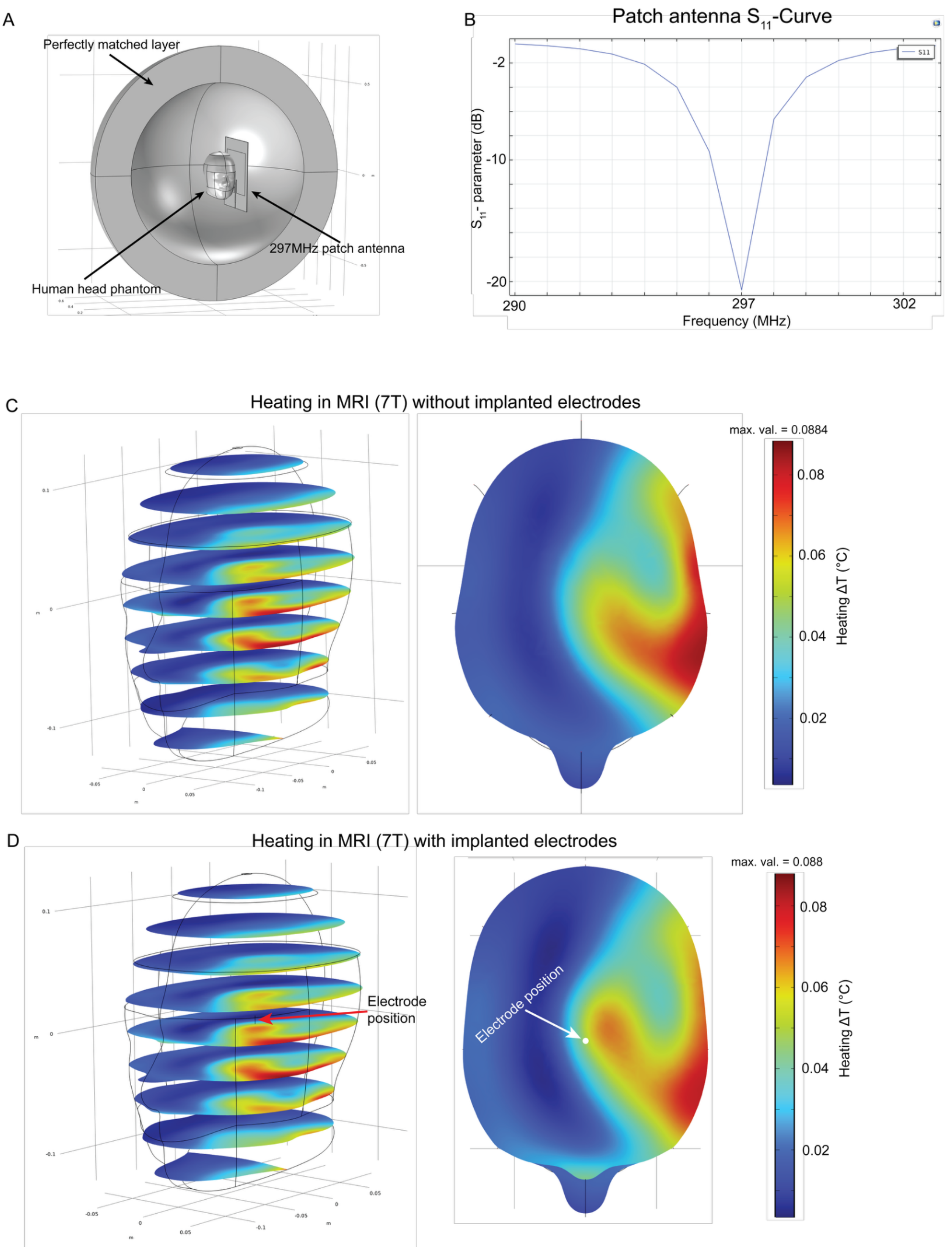

NEW Suppl. Fig. RF-induced heating of human-phantom in 7T MRI with and without the MRI-tagged UFTE electrodes under the RF-excitation frequencies used in this study. **A.** Full-wave simulation setup shows the perfectly matched layer, human head phantom and patch antenna placement. **B.** Custom patch antenna which is operating at 297MHz (7T MRI H1 frequency). **C.** Heating in MRI without MRI-tagged UFTE electrodes. **D.** Heating in MRI with MRI-tagged UFTE electrodes.

- The development rationale is unclear. Did the authors go directly from concept ideation to in vivo implementation? Were there any simulations carried out before? Or were there any in vitro or ex vivo/cadaveric tests carried out with brain phantoms or cadavers?

We again thank the reviewer for this very valuable advice. Indeed, prior to *in vivo* experiments*;* we had carried out many phantom tests (Agarose gel, 2% concentration in ultrapure water) in MRI (both 7T and clinical 3T scanner) where electrodes were implanted into phantoms. Electrodes were fully coated (not barcoded) with IONP in these tests. We have now included these details in the manuscript in the supplementary materials.

Please also see below an example figure from our phantom tests (Agarose gel, 2% concentration in ultrapure water) in MRI (7T pre-clinical scanner and 3T clinical scanner).

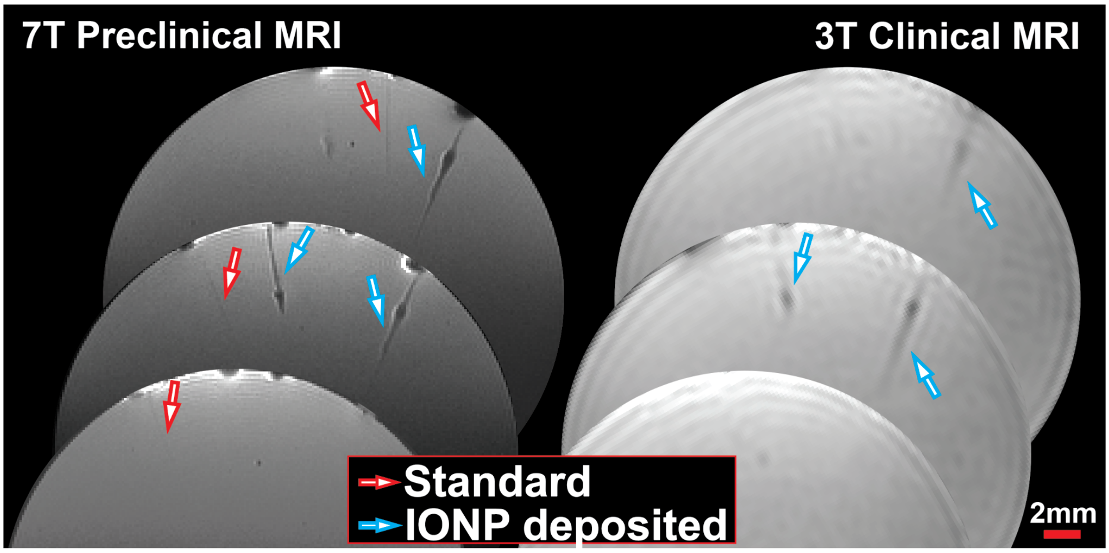

NEW Suppl. Fig. Four UFTE bundles are implanted into agarose gel phantom (n=2 IONP deposited, n=2 standard). Phantom is imaged in both 7T preclinical and 3T scanner.

- How does the implementation of embedded IONP-coated structures affect the long-term stability (for more than six months in biological conditions) of the implants? Iron oxide is biodegradable, if there is any kind of water intrusion, there is a risk that IONP tags degrade. Were there any accelerated aging tests carried out?

  We again thank the reviewer for this very valuable advice. We had not conducted accelerated aging tests. Indeed, in the event of delamination of polyimide layers, IONPs might get exposed to tissue, and IONP tags might degrade together with the electrodes, losing their recording functionality. Our preliminary data shows this is not the case. UFTEs were previously tested in mice for 1 year-long chronic implantations (Yasar et al. 2024) where there was no degradation nor delamination of polyimide fibers confirmed with regular impedance measurements.

  Please also find below an additional impedance data where we implanted an animal with two MRID-tagged UFTE bundles over 6-months (animal is still alive and implanted for another project). Please note that MRID-tags exhibit similar contrast intensity profiles even after 6 months-long implantation suggesting that there is no delamination.

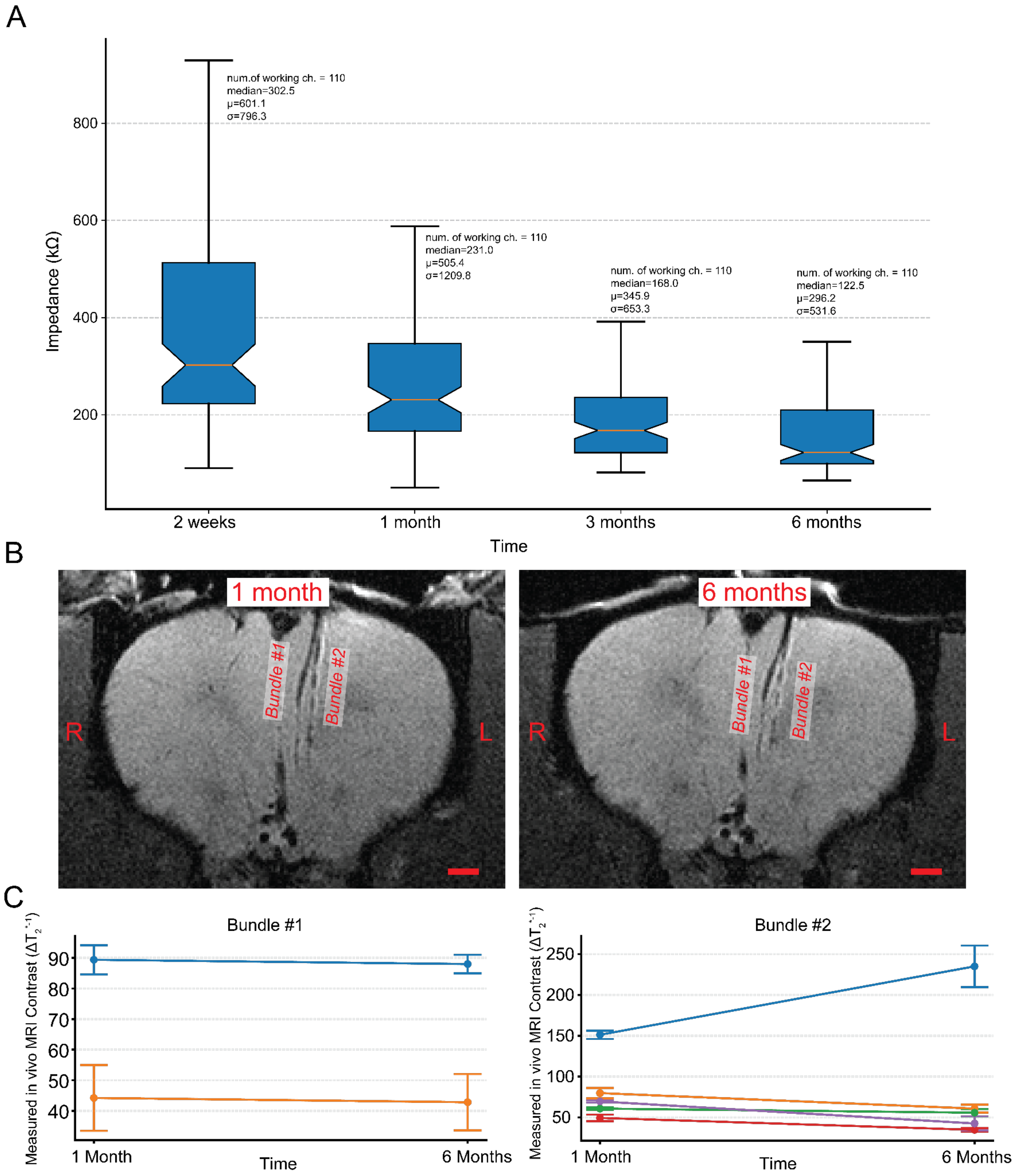

NEW Rebuttal Figure. Chronic stability example of an MRID-tagged UFTE A. Chronic impedances from two MRID-tagged UFTE bundles implanted in the rat brain over 6 months with descriptive statistics given at each time point (μ: mean and $\boldsymbol{\sigma}$: standard deviation in kΩ). **B.** Two MRI images show the two MRID-tagged UFTE bundles 5 months apart (6 months total implantation duration). Scale bars, 1mm. **C.** Chronic mean MRI contrast induced by MRID-tagged bundles given, each color denotes an IONP-island.

- For chronic applications, and especially when considering human applications, device sterilization is of utmost importance. What sterilization method was used, or which sterilization method could be compatible with the approach presented? How can device sterilization affect long-term stability?

  We thank the reviewer for raising this important point regarding device sterilization for chronic and translational applications.

  In the chronic animal experiments, electrodes were prepared under sterile environments, and animals received postoperative antibiotic treatment to prevent

infection. However, the electrodes themselves were not subjected to pre-surgical sterilization procedures.

For clinical studies, robust sterilization protocols are essential. To address this, we have been conducting a parallel study focused on the sterilization of MRID-tagged UFTEs. We demonstrated that ethylene oxide (EtO) sterilization works with our MRID-tagged UFTEs (as it is commonly used for temperature-sensitive biomedical devices and is compatible with polymer-based materials).

Please see below the data and figure from our sterilisation study for your convenience. We now added this data into Supplementary Figures of the manuscript.

A

	15min	30min	1h	2h	3h	4h	12h	24h	48h	72h
**EO Protease**	-	-	+	+	+	+	++	++	+++	+++
	-	-	++	++	++	++	++	++	++	++
**Ctrl Protease**	-	-	+	+	+	+	++	+++	+++	+++
	-	-	+	+	+	+	++	++	++	++
**EO PBS**	-	-	-	-	-	-	-	-	-	-
**Ctrl PBS**	-	-	-	-	-	-	-	-	-	-

B

-	bundle is intact
+	fibers disentangled slightly from bundle tip (~1mm)
++	fibers disentangled mid-bundle (~2-3mm)
+++	completely disentangled fibers (not bundled anymore)

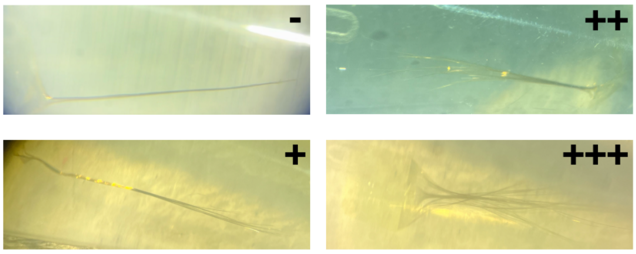

C

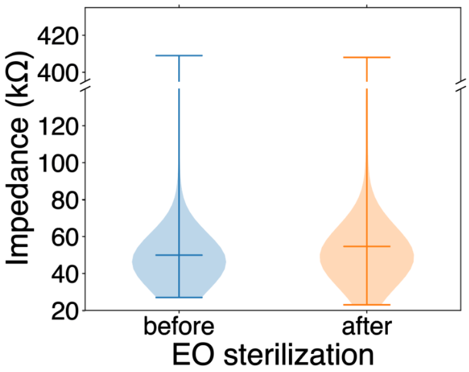

NEW Suppl. Fig. in vitro tests of sterilised MRID-tagged silk-fibroin coated UFTE bundles. **A** In vitro degradation of EO sterilized silk fibroin coating. Ctrl = Control, EO = ethylene oxide sterilization, PBS = incubated in PBS solution. **B** The scale used for the visual evaluation of the degradation. Example pictures of degradation stages are shown. **C** Impedance measured in two electrodes before and after EO sterilization. The distribution of the channel impedance is depicted in a violin plot. Only functional channels (defined as <500 kΩ or >25 kΩ before and after sterilization) are considered.

- More detailed discussion on individual results is missing. Results are not compared with other state-of-the-art imaging techniques used to localize electrodes, such as X-rays or CT? What about using devices with functional MRI? How does this approach compare with other approaches using MRI-compatible neural implants?
  In the discussion, we now included a comparison for localizing electrodes with other state-of-the-art imaging techniques:

  "Ultra-flexible electrodes similar to UFTEs have been detected only in post-mortem tissues (detected by micro-CT, Zhao et al. 2023). Markers could be used to enhance contrast in CT. For example, a radiopaque special-geometry marker was used (Hellerbach et al. 2018) to determine the orientation of bi-directional deep-brain stimulation (DBS) electrode leads post-operatively in CT scans. However, the amount of metal (e.g. gold) used in radiopaque markers in DBS probes is orders of magnitudes larger than the UFTEs can accommodate."

We also included a brief discussion of fMRI compatibility:

"MRID-tagged UFTEs could also be potentially used during fMRI. However, the susceptibility-induced artifacts from MRID-tags might interfere with the quality of fMRI signal within the proximity of electrodes, depending on the particular implantation site, and fMRI imaging sequences used."

- What is the rationale for selecting IONPs over other materials (e.g., gadolinium) for the same purpose? The authors should justify and discuss material selection. While our technique is readily adaptable to use other contrast agents, the primary reasons for selecting IONPs over other materials are the following:
    - T1 contrast enhancement (e.g. using Gd^{+3}) relies primarily on inner- and secondarily outer-sphere effects and requires close proximity between the water molecules and paramagnetic ions where the effect is inversely proportional to r^6 (r: distance between water proton and paramagnetic ion) (Rümenapp et al. 2012, Caravan et al. 1999). However, our technique encapsulates contrast agents in between polyimide layers (1 µm thickness each) that creates a large separation between the paramagnetic ions and water molecules. Such large separations significantly reduce the T1 contrast.
    - Our fabrication technique clusters contrast agents (IONP) where susceptibility-induced static dephasing effects become dominant (T2* contrast). IONP becomes a better choice due its superparamagnetic properties, inducing larger susceptibility effects with lesser amounts.
    - We use multiple scans (at different effective echo-times) throughout the relaxation to accurately pinpoint the center of mass of the IONP bodies. T2/T2* relaxation times are usually within <100ms, whereas T1 relaxation times are >500ms up to seconds. Therefore, using IONP allows shorter scanning times of the subjects.

  We have now added this to the discussion in the revised manuscript.

- The IONP tags are permanently implanted along with the neural threads. Are MRI scans still clear enough to distinguish the surrounding anatomical structures in the brain?

  Yes, MRI scans are clear enough to distinguish surrounding anatomical structures, such as the borders between the dorsal hippocampus (dHPC), corpus callosum (CC), and thalamus as shown below. The amount of IONPs deposited in the MRID-tags can be reduced (at the expense of total scan time) depending on the application and the target brain region, ensuring that anatomical contrast is preserved, and surrounding structures remain clearly visible.

  Please see below an example MRI image as an example where all anatomical structures and borders are clearly differentiable.

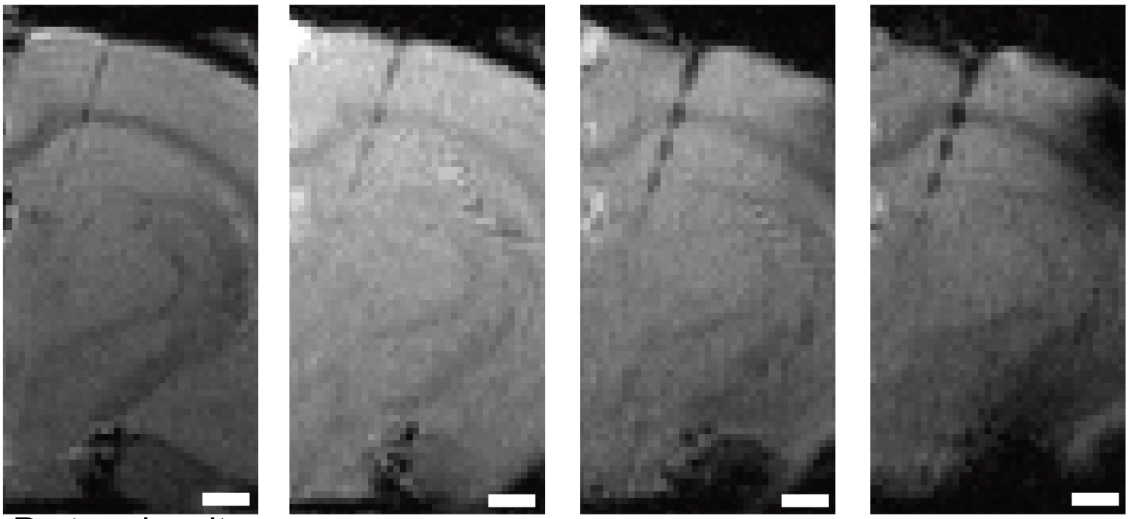

NEW Rebuttal Fig. Sample raw MRI images of an MRID-tagged UFTE bundle. From left to right; proton-density RARE image, increasing TE (4 ms, 12 ms, 20 ms) images from T2*-weighted MGE.

Line-by-line comments:

- Line 42-45: It reads like a random sentence. It should be mentioned as an example of flexible neural probes with a minimal footprint. It would fit better to annex the sentence at the beginning of the paragraph starting in line 60 'Here, we developed magnetic resonance identification (MRID) tags to identify UFTE bundles...'.

  We now modified the manuscript according to the reviewer's suggestion. The example of ultra-flexible tentacle electrodes (UFTEs) with minimal footprint has been moved to better support the context of our method development:

  "... Here, we developed magnetic resonance identification (MRID) tags to uniquely identify ultra-flexible electrode shanks while also localizing individual electrode channels in MRI scans. MRID-tags (made of superparamagnetic nanoparticles) generate custom barcode patterns visible in MRI images unique to each ultra-flexible electrode shank, which we call MRI-barcodes (Fig 1A, see Results). After the identification of an ultra-flexible shank using MRI-barcode, the individual bars along the MRI-barcode allow an accurate 3D reconstruction of the ultra-flexible shank trajectory. Subsequently, we localize the individual electrode channels along the reconstructed shank trajectory by using their fabricated distances relative to the patterned MRI-barcodes (Fig 1A), with mean 95 um accuracy validated electrophysiologically (see Results).

  Our MRID-tags are made of MRI (T2/T2-weighted) contrast agents i.e. superparamagnetic iron(II, III)-oxide nanoparticles (IONPs)[17–20] (Fig. 1B). MRI contrast agents are substances (e.g. IONP and gadolinium etc.) that change the local magnetic properties to enhance the image contrast to identify specific anatomies and pathologies. We coat IONPs into individual ultra-flexible electrode fibers in unique barcode-like patterns as dot-matrices (Fig. 1B). Each dot in the matrix is an assembly of thousands of IONPs, as shown in Fig. 1B. The IONP dot-matrix alters the local magnetic properties of neighboring tissue, thereby yielding MRI barcodes.*

  We tested the MRID-tags embedded in 64-channel (50 um center-to-center spacing between recording sites) ultra-flexible tentacle electrodes (UFTEs) (Yasar et al 2024) chronically implanted into rat brains to assess MRI-barcodes and electrode localization in vivo. UFTEs offer arbitrary depth and angle penetration capability, a minimal electrode

footprint, high-density and distributed recording sites, high SNR, and months-long brain activity recordings facilitated by a novel implantation technique[9] (Suppl. Fig 1). We used Waxholm space (WHS) rat brain reference atlas for anatomical labeling[24]. The electrode localization accuracy was validated against established dorsal hippocampal (dHP) electrophysiological landmarks—namely the pyramidal layer (PyL) and hippocampal fissure (HF) identified from local field potential profiles, with ripple power (150–200 Hz) components of sharp wave ripple (SWR) peaking in the pyramidal layer and theta power (7Hz) reaching a maximum near the HF.[6,25-27,28–31] …"

- Line 54-56: Can you also comment on the efforts to use markers for other imaging methods, such as X-ray?
  We addressed the reviewer's comment on this above in response to a related question where we added to the manuscript the following in the discussion:

  "...Existing clinical or NHP electrodes are readily visible in CT and MRI scans because of their large geometry and metal contacts, yet imaging markers on electrodes are still necessary for more accurate and quantified electrode localization. For example, specific-geometry radiopaque markers are utilized on deep-brain stimulation electrodes to determine the orientation of bi-directional stimulation leads in CT scans (Hellerbach et al. 2018)..."

- Line 56-58: Other pre-clinical methods might have been overlooked. For example, can you comment on the pros/cons of other imaging methods, such as fluorescent indexed barcodes mapped through confocal/multiphoton microscopy (Yang et al., Bionspired neuron-like electronics. Nat Mat, 2019)?
  Thank you for bringing this work to our attention. We now addressed this work in the manuscript:
  "...For post-mortem analysis (typically in small animal models), optical microscopy can be used to visualize electrode trajectories after coating the probes with fluorescent dyes prior to implantation[14] or performing electrolytic lesions. However, both methods yield only qualitative results due to dye diffusion and lesion sizes. Fluorescent barcodes incorporated to the electrodes have been proposed to achieve high-spatial resolution of the electrode locations[15], yet this requires electrodes to remain in place during tissue processing, which is too challenging, especially during slicing…
  "

- Line 61-62: What are the challenges of introducing imaging tags in neural implants concerning material and imaging method? Additionally, could you first introduce MRI contrast agents? What are they used for?
  We now moved the discussion and choice of MRI contrast agents to the results section (from Introduction) and gave a higher perspective view of the paper in this paragraph (as the reviewer suggested in previous comments). In the results section, we explained the different imaging MRI contrast agents used with the different imaging methods.

- Line 64-66: These terms need to be introduced briefly before using them. What are "topography-controlled capillary" and "convective assembly"?

  We have revised the manuscript to briefly introduce these concepts before their first use.

  "... topography-controlled capillary and convective assembly (CCA)[21–23] with a dragging meniscus of nanoparticle colloidal suspension. Convective assembly relies on evaporation-driven convective flow at low contact angles to deposit continuous nanoparticle layers, whereas capillary assembly uses meniscus pinning and capillary forces at higher contact angles to selectively trap and deposit particles in confined regions[21–23]…"

  These definitions have been added to the relevant paragraph in the Results section to improve clarity and accessibility for non-specialist readers.

- Line 60-79: It reads more like part of the conclusion.
  We have now revised this section to better fit the purpose of the Introduction.

- Line 77: Please introduce the abbreviation when used for the first time in the text.
  We have now revised the manuscript to ensure that the abbreviation is introduced at its first occurrence.

- Line 77-78: What are theta rhythms and SWRS? What are the main features that characterize these oscillations?
  Theta rhythms (4–10 Hz) are low-frequency oscillations that occur during exploration and REM sleep. In the CA1 region of the hippocampus, theta waves exhibit a laminar phase shift, with the phase gradually advancing from the pyramidal layer (stratum pyramidale) through the hippocampal fissure (HF) toward the dentate gyrus molecular layer. This systematic shift in phase and amplitude along the dorsoventral axis provides a spatially resolved marker of electrode depth and orientation.

  Sharp-wave ripples (SWRs) are brief (~100ms), composite oscillatory events that include: A low-frequency sharp wave (~1–20 Hz) originating from CA3 inputs, and A high-frequency ripple oscillation (150–250 Hz), localized primarily to the CA1 pyramidal layer. The sharp wave component is generated by synchronized depolarization of apical dendrites in stratum radiatum, where CA3 Schaffer collateral axons terminate. As a result, the largest negative deflection of the sharp wave is observed in the CA1 stratum radiatum, while the signal reverses polarity in adjacent layers (e.g., stratum oriens). The ripple component, by contrast, reaches its maximal amplitude in the stratum pyramidale and rapidly attenuates in deeper or superficial layers. The hippocampal fissure serves as a physiological boundary, where ripple activity drops and theta phase saturates. In the dentate gyrus, both ripple and sharp-wave amplitudes are minimal or absent.

  To further illustrate this, we have added a new supplementary figure (Suppl. Fig. 6.3), which shows data from bundle #1 (left hemisphere) and bundle #2 (right hemisphere) as raw LFP traces together with the corresponding averaged wavelet transformations of theta segments. For comparison, the averaged wavelet transformation of SWRs is shown in the main Figure 4C (second column). These additions, together with the **supplementary video** of theta during locomotion, make the physiological rationale more transparent and accessible to a broader audience. (See the NEW Suppl.Fig 6.3 below this line:)

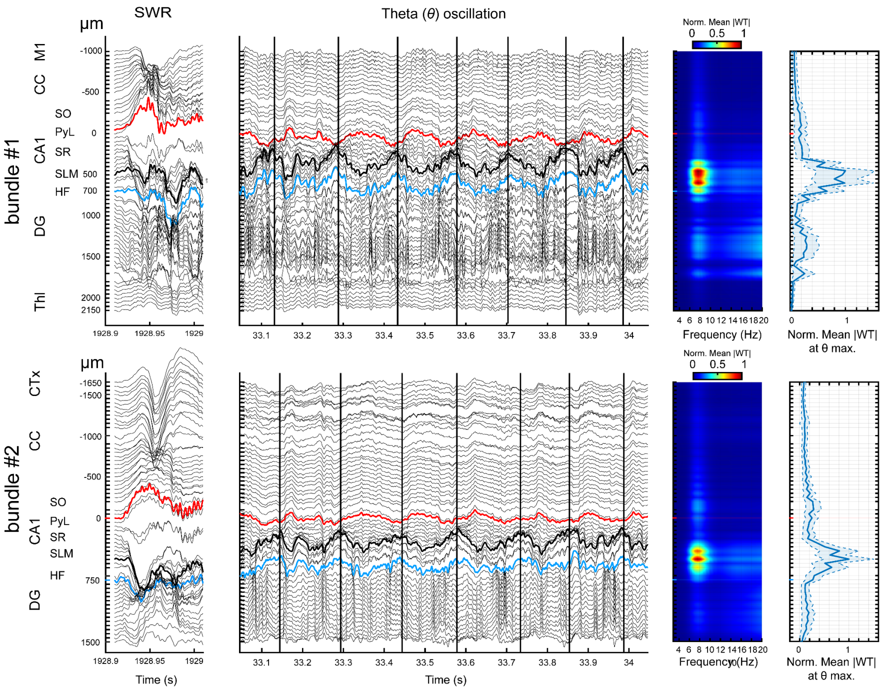

NEW Suppl. Fig. 6.3 The first column shows a representative LFP traces (1–300 Hz) containing a sharp wave ripple (SWR). The second column displays a simultaneous 1-second LFP segment (1–300 Hz) illustrating theta oscillations recorded from bundle #1 (left hemisphere, top row) and bundle #2 (right hemisphere, bottom row). The x-axes of the rows are aligned, so that each tick mark represents the same time point across rows, ensuring that the SWR trace and the theta-segment LFPs correspond to simultaneous activity in the left and right hemispheres. The vertical solid black line indicates the peak of the theta oscillation in SLM. The third column presents the averaged continuous wavelet transformation of the theta segments (n = 2306 cycles) across all contact sites of the bundle. The fourth column shows the mean wavelet transformation (solid line) and ±SD (dashed lines) at the point of maximum theta power across all contact sites of the bundle. The y-axis represents the distance between contact site channels in µm. In the raw LFP data, red lines indicate the beginning of the pyramidal layer in the CA1 dorsal hippocampus, the blue line marks the hippocampal fissure (HF), and the thicker black line indicates the location of maximum theta power in SLM.
Abbreviations: M1 – motor cortex; CA1 – Cornu Ammonis 1; DG – dentate gyrus; SO – stratum oriens; PyL – pyramidal layer; SR – stratum radiatum; SLM – stratum lacunosum-moleculare; HF – hippocampal fissure.

- Line 97: Although it was briefly introduced in the Introduction, what are IONPs? What are the requirements for their selection? For example, NP size, material biocompatibility, MRI compatibility, etc.
  IONPs (iron-oxide nanoparticles) are commonly used as MRI contrast agents. The main requirement for their selection is their size (i.e. diameter) which determines their superparamagnetism. We have now extended the introduction of IONP and the requirements of their selection in the introduction and discussion sections. As mentioned above, we also explained the choice of IONPs with respect to other potential contrast agents.

- Line 100: It reads as if the recording sites were connected to active electronics. Is this the case? If not, please remove the word 'active', as it implies the use of active electronics.
  We thank the reviewer for this advice. We now removed the word 'active' in the manuscript.

- Line 102-104: At this point, it's hard to get a sense of the dimensions of the coating (is it a monolayer of IONPs?) and the components of the barcode, e.g., the circular assembly, the individual dot of the dot matrix, and the barcode itself, what does it encode for. It would improve clarity if you avoided using different names for the same concept and just mentioned the exact dimensions or the range of dimensions. In addition, including labels for what a dot, or a circular assembly, and a barcode are in Figure 1A, would improve readability. Are there any constraints that limit the size of the IONP coating thickness, or dimensions?
  We thank the reviewer for this helpful suggestion. We have now implemented his/her feedback in the corresponding results section. We also now included the nomenclature we used in Figures.

  The IONP coating thickness is limited by the photoresist thickness and desired polyimide sandwich thickness, as thicker IONP dots contribute thicker fibers.
  The IONP dot dimensions are limited by the desired dimension of the individual electrode fibers as they must be fully encapsulated.

  Each barcode on a bundle encodes the identity of the individual bundle. Its geometry can be generated by a random generator using the predefined pitch-width, number of bars and total length of barcode in real-life units (e.g. micrometers, limited by electrode bundle length). In this manuscript, we showed randomized examples generated under these conditions. We have tested barcodes with 300 µm pitch-width, 4500 µm barcode length and 2,3,4, and 5 bars. We now explain this in the manuscript.

- Line 102: It's unclear what modularly implies in this context. Please explain.

“Modularly” means the IONP dots are physically separated from each other which has a minimal effect on the ultra-flexible electrode bundle’s mechanical properties (e.g. flexibility). We have now clarified this in the Results section.

- Line 110: Please introduce the abbreviation of “dH2O”.
  We have now introduced the abbreviation of dH2O as … ultra-pure deionized water.

- Line 112-114: It would be helpful if CCA coating were introduced before, so that the method is clear. What is the difference with a 'doctor blade coating'? Are there nanoparticles left at the surface of the photoresist mask or only at the openings?

  As the reviewer suggested earlier, now we moved the mention of CCA from the Introduction section to the Results section, where we further explain the CCA method and how we modified it.

  Difference from doctor blade coating: Although the two techniques are similar, we take advantage of the controlled moving meniscus here. When the right conditions are met (contact angle, colloidal suspension concentration, sample temperature, moving speed of meniscus), our technique ensures that laminar flows guide nanoparticles into recessed traps in the dot-matrix. Thus, the traps are much more uniformly filled with the nanoparticles.

  We optimized the colloidal suspension concentration and movement speed to prevent bridges from IONP assemblies in the traps to nanoparticles left on the photoresist mask. This ensures a clean lift-off performance. We had confirmed this by SEM. We now added SEMS images to the Supplementary Material section.

  Please see below the corresponding figure for your convenience.

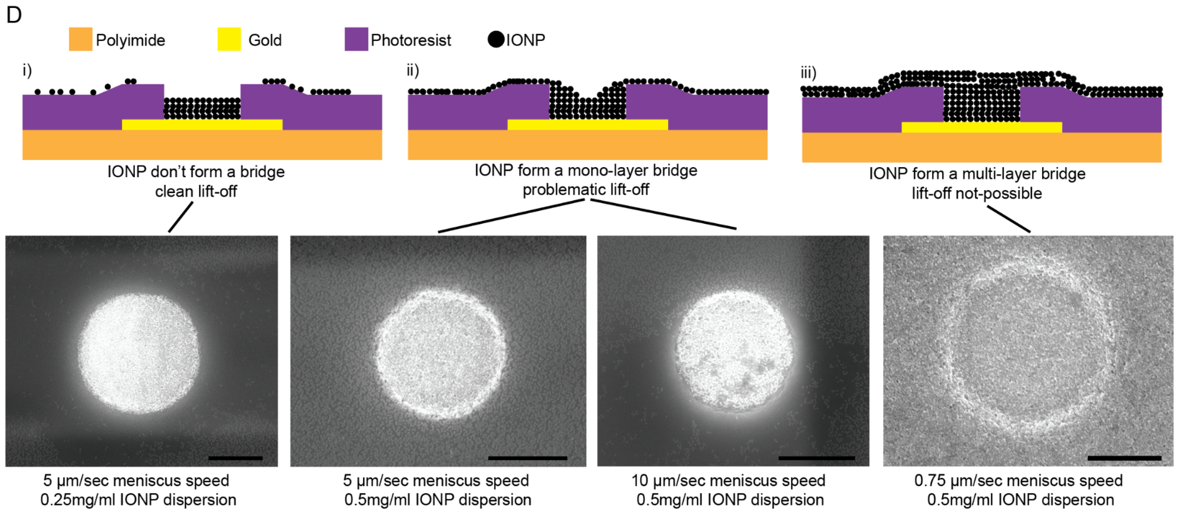

NEW Suppl. Fig. Top-row shows the cross-section of the sample for possible outcomes of IONP coating at different meniscus speeds and IONP dispersion concentrations. The bottom row shows the exemplary SEM images for each outcome at given coating parameters. Scale bars denote 1 µm.

- Line 111: Can you elaborate more on the confinement slide? What is it? Fig. 2B is unclear in this regard.
  We have now used the term ‘glass slide’ instead of ‘confinement slide’ and indicated it in the figure, and we explained in the figure caption that it is a glass-slide horizontally positioned 500 µm above the sample (i.e. wafer) used to confine the colloidal suspension (where we inject the IONP dispersion in ultrapure water in between this slide and the sample).

- Line 117: Recessed dot-matrix topography = trap? Please be consistent along the text using the same words to avoid confusion when reading.

In the text, the term “trap” refers to the “recessed trench-like feature” of the dot-matrix topography that is designed to capture and retain nanoparticles. To improve clarity and consistency, we have revised the text to explicitly state this connection earlier in the paragraph, and we now termed it as ‘dot-matrix traps’.

- Line 123: What is meant by "intermediate contact with the Au surface below (Fig. 2C)"?
  We changed ‘intermediate contact’ to ‘partial contact’, which is the contact that meniscus makes right after it breaks from the pinning edge at the photoresist, therefore it is a partial contact with the Au surface below. We have further clarified the description of this in the Results section.

- Lines 124-128: The SEM images provided do not give enough detail to observe this. please provide images with a higher magnification or with a better quality, e.g., the SEM image in Fig. 2C.
  We apologize for this inconvenience which is probably due to the PDF version of the submission. We have now included high-quality figures separately for better readability and also in response to the reviewers previous questions as mentioned above.

- Lines 134-137: Did you do adhesion tests of the IONP dots? Can the IONP coatings affect the adhesion of the PI sandwich?
  Indeed, during the lift-off, the IONP dots might detach from the smooth surfaces. To increase adhesion of IONP to Au, we increased the effective contact area with the Au by chemically roughening the Au surface as now further clarified in the Results/Methods.

  The delamination of the polyimide layers would reflect as changes in the impedances of the devices. The impedances of our IONP-coated UFTEs do not show any detectable impedance differences than our standard UFTEs both in vitro during aging tests and also in vivo months-long recordings. We now clarified this in the Results.

- Line 134: As commented before, please revise the quality of the SEM inset image, Fig. 2C inset.
  We have now revised the quality of SEM images, which were degraded during the submission of the manuscript. We also now uploaded these images separately as high-quality files. We will also double-check the SEM image qualities during the proofing stage of the manuscript if accepted.

- Line 146: Please revise the quality of the SEM images, they look blurry.
  We have now revised the quality of SEM images, which were degraded during the submission of the manuscript. We also now uploaded these images separately as high-quality files. We will also double-check the SEM image qualities during the proofing stage of the manuscript if accepted.

- Lines 148-149: How do the quality of the IONP coatings differ? The quality of the SEM images is poor, therefore, it’s hard to tell if there is a difference between IONP coatings on smooth Au or on PI, as there is a clear difference with coatings on porous Au. Why do the IONPs need to be coated onto the Au surface? Could they be also encapsulated or embedded within the polyimide layer?
  We now removed the SEM image of IONP coatings on polyimide because we only coat Au surfaces. We now briefly mention that our method can be used to coat polyimide and refer to the Supplementary Materials for the SEM image of IONP coated polyimide.

  The reason that we utilized Au surface instead of polyimide surface is because Au surface is an intermediate layer at a precise depth from the surface and covers most of the device area (including the pads and the cables). This helps us avoid the additional processing of polyimide etching and/or another layer of polyimide patterning. We now clarified this further in the Discussion.

- Line 153: It seems that there is a misalignment of the barcode, or are these imaging artifacts? In the case of a misalignment, is this caused by the lithography of the deposition mask or by the lift-off/stripping of the photoresist? Also, what would be the effect of a misalignment on the reading accuracy of the barcode?
  The image was from one of our earlier test devices. We now put a representative image of our devices. Such misalignments can be caused by the lithography of the deposition mask. Please however note that such misalignments (even if it is as or more significant than the one shown in the image) do not compromise the devices because:
  (1) The SEM image is showing only the metal layer and the barcodes. The polyimide layers (not observable in this SEM image) at the bottom and top indeed fully encapsulate the IONP traps. We know they are fully encapsulated from the impedance measurements as mentioned in response to the reviewer's previous questions.
  (2) We inspect each electrode bundle after the lithography, and do not tolerate misalignments larger than ±1 µm. Since our tolerance is 2 orders of magnitude smaller than the pixel size of MRI, this does not affect the reading accuracy of the barcode.

  We have now replaced the corresponding SEM image and also clarified these further in the manuscript and figure.

- Line 186: What is the exact MRI voxel size?
  The exact voxel size is 136 x 136 x 800 micrometers. We now clarified this in the Results section.

- Line 189: The comparison is not easy to imagine because thickness, area, and voxel are three different units. In this context, what is the bundle thickness? Isn't the thickness the same for each individual fiber? In the case where the thickness refers to the overall or cumulative thickness of all fibers, isn't it then affected by the assembly of the bundle on the shuttle together with the silk coating? How accurate are the dimensions of the coating and the spatial arrangement of the fibers forming a bundle?
  Thank you; we now corrected this inconsistency in units.

  Each bundle is formed from the individual electrode fibers (where the IONP barcodes are at identical longitudinal positions across all fibers within a bundle). Thus, the MRI image detects the barcode signal from all the fibers of a bundle. This is why we compare the bundle dimensions to the MRI voxel sizes.

  We now provide the thickness of the bundle after self-assembly in the silk coating (without including the exterior silk coating thickness which are not visible in the MRI anyways). This diameter is significantly smaller than the MRI voxel resolution. After the implantation, as the silk coating dissolves, the fibers get dissociated in the tissue. However, this does not affect our barcode readout (as we have shown in longitudinal MRI scans across weeks to months) indicating that the bundle diameter still remains within voxel dimensions.

  Fig 3.C shows that our barcodes measured by MRI match very well the barcode patterns expected from wafer-level layouts, indicating that our assembly process does not significantly disturb the spatial arrangement of barcodes.

  Please also find below the **new** supplementary figure for bundle thickness measurements.

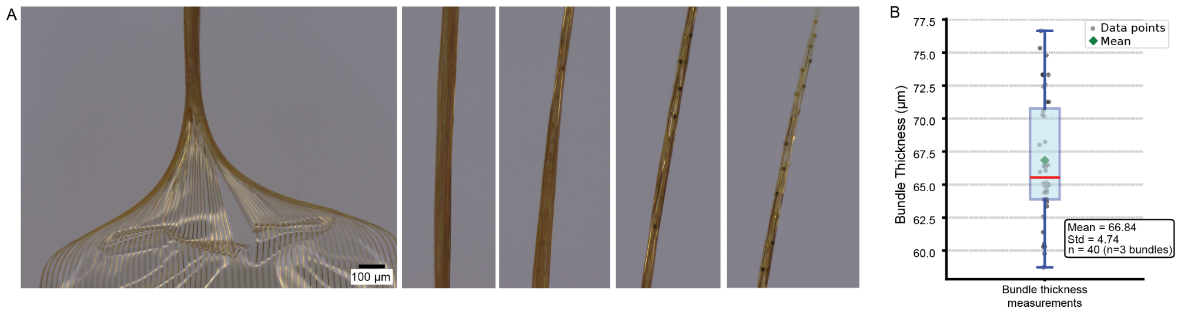

NEW Suppl. Fig. Silk-fibroin coated MRID-tagged UFTE bundle thickness measurement. **A.** Optical microscopy images of the electrode bundle from dorsal to ventral (left to right). **B.** Bundle thickness measurements (all 64-fibers) box plot and descriptive statistics (n=40 measurement points, n=3 electrode bundles).

- Line 195: "T2*-weighted contrast" has not yet been introduced. Please add more context and a definition to the concept. The sample applies to "TEs".
  We have introduced and explained more in depth the T2*-weighted contrast and TEs in the manuscript.

- Line 202: Please specify that sigma is the standard deviation.
  We have updated the manuscript at Line 202 to clarify that sigma refers to the sigma parameter of the Gaussian curve-fit (i.e. standard deviation for distributions), as requested.

- Line 205: Are there any other patterns used? Is there a rationale behind the pattern design of the barcode? What do they encode?
  The main role of the specific barcode pattern is to differentiate the electrode bundles from each other. In this work, we simply changed the number of stripes (or bars) in a barcode to encode and differentiate the individual bundles from each other (when multiple bundles were implanted into one animal).

  As we also mentioned previously: Each barcode on a bundle encodes the identity of the individual bundle. Its geometry can be generated by a random generator using the predefined pitch-width, number of bars and total length of barcode in real-life units (e.g. micrometers, limited by electrode bundle length). In this manuscript, we showed randomized examples generated under these conditions. We have tested barcodes with 300 µm pitch-width, 4500 µm barcode length and 2,3,4, and 5 bars. We now explain this in the manuscript.

- Line 207-209: Could you please give an example of the baseline taken or please rework the wording of the sentence to provide clarity.
  MRI provides the soft-tissue contrast which causes different anatomical regions to appear at different pixel intensities in the images. To accurately detect, if a pixel is contrastive, we first identify in which anatomical region that pixel is located. For example, corpus callosum appears darker (i.e. lower pixel intensities) than cortical or thalamic regions in T1 and T2/T2* weighted images.

  We have now clarified the wording.

- Line 207: In relation to Fig.3B, please provide the original images without the green annotations referring to the quad pattern detected MR image pixels in the supplementary figure or in the same figure panel.
  We have now provided the original images from two samples in the Supplementary Materials following the reviewer's comment.
  Please also see below the corresponding Supplementary Figure below for your convenience.

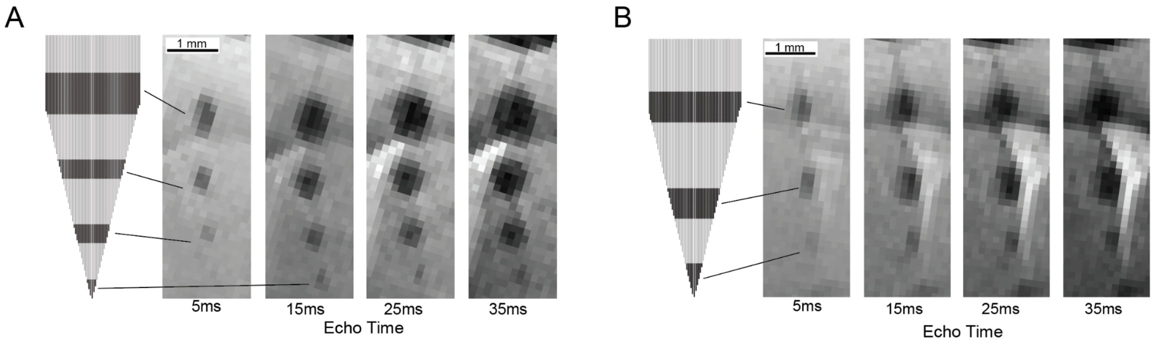

NEW Suppl. Fig. Raw MRI T2*-MGE images of two sample MRID-tagged UFTE bundles at increasing echo times (TEs). **A.** MRID-tag with the quad-pattern MRI-barcode. **B.** MRID-tag with the trio-pattern MRI-barcode.

- Line 212: Could you please briefly expand the description on the sliding window?
  We slide a 3x3 pixel image window across the MRI image. We have now expanded the description of the sliding window both in the Results and Methods sections.

- Line 214: "heatmap contrast map" reads redundantly. Perhaps it sounds better referring to it as "contrast heatmap". Would it be possible to include additional information in the supplementary explaining how the heatmap is generated with sample data in between steps?
  We revised the wording to 'contrast heatmap' for clarity and conciseness. We have now provided a Supplementary Figure showing step-by-step analysis on sample data.

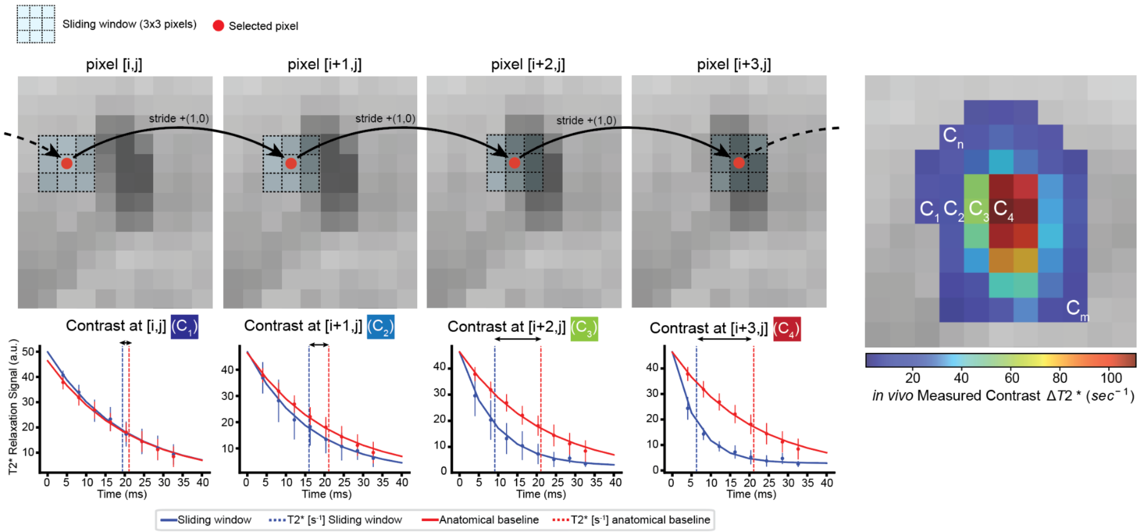

NEW Suppl. Fig. MRID Analysis, contrast heatmap generation. A sliding window is moved over (in both x- and y- axes, with stride:1) where the MRID contrasts are. At each pixel, mean pixel intensities from the sliding window are compared to the mean pixel intensities from anatomical baseline across increasing echo times. Relaxivity time constants are calculated for MRID and baseline curves, where the difference gives the contrast intensity. A heatmap is generated from each measured contrast intensity.

- Line 220-221: What does this mean experimentally? Would it be possible to use such tags in highly-dense electrode arrays that have more than one electrode per fiber closely packed? What are the limitations?
  This result means experimentally we can reconstruct the bundle trajectory by 28.70 µm median error in 3D, which is sufficient to approximate the relative spatial organization of our independent channels with 50 µm separation. (We now updated the results and corresponding Figure 3C with the new data added from revision experiments (n= +4 animals). We also changed the metric to “absolute difference” yielding an absolute median error of 50.15 µm with n-number doubled (n: 16 → n:32).)

If there are multiple electrodes on the same fiber, this approximation becomes even better because the electrode-electrode distances are fixed with respect to each other on each fiber.

- Line 227: These predefined constraints need to be described from the beginning.
  We have now described and further clarified these predefined constraints from the beginning in the Results and Methods section.

- Lines 231-233: It's unclear how the MRID tags allow identification of individual electrode fibers within a bundle. It appears that MRI is good enough to identify the IONP island or cluster of electrodes, but not to accurately identify the location of individual electrodes, which is still a computational method based on the design features of the device and not the imaging method per se.

  The MRID tag enables reliable identification of each electrode bundle, including the position of the IONP barcode along its length. While MRI does not directly resolve individual contact sites, the location of the barcode is known from the device design and fabrication process, relative to the electrode geometry and contact site layout. Therefore, once the electrode and barcode are detected in the MRI image, we infer the position of each contact site with high confidence based on this known spatial relationship.

  The accuracy of this inference is confirmed by our high-resolution electrophysiological recordings of well-defined activity signatures across CA1 laminar structures (as we discussed above in response to the reviewer's other questions and also in the manuscript).

- Line 236, Fig. 3E: How do these measurements vary between samples? Is there a way to show the variability (e.g., standard deviations) between samples visually? Could you perhaps add the additional example plots as in Fig. 3B, but for different samples (bundles and animals)?
  We now updated the Figures showing the variance including the new data shown below. We also added example contrast heatmaps from different animals and bundles into supplementary material.

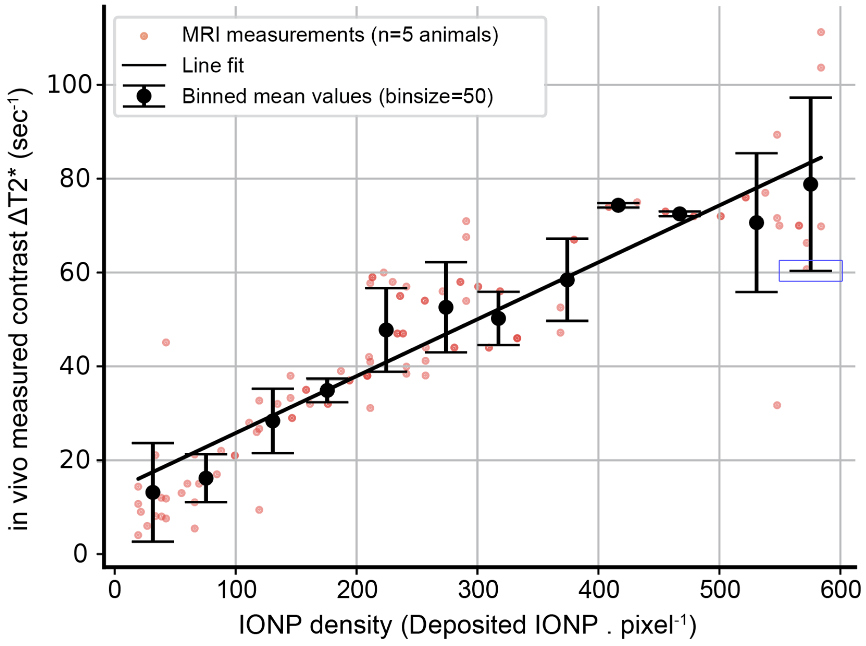

REVISED Figure 3.E. *in vivo* measured MRI contrast levels (n=5 animals) as a function of IONP coating density showing the linear correlation with best-fit line (**black**).

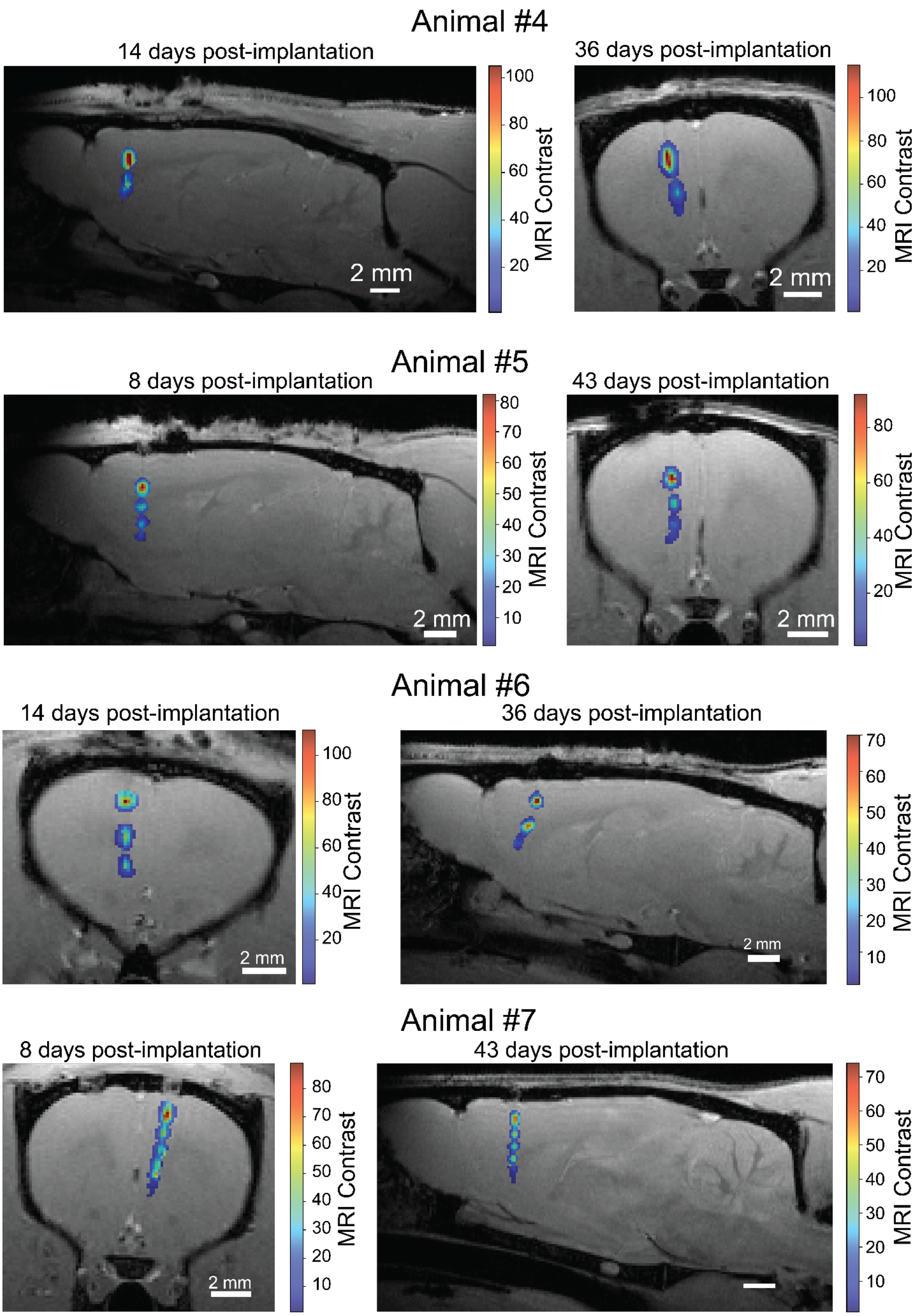

NEW Suppl. Fig. Four examples for chronically implanted MRID-tagged UFTE bundles to show the contrast heatmaps across animals, bundles and time.

- Line 267-291: Please introduce the figures of merit used to validate the physiological landmarks of the activity recorded. What is the biological meaning of the ripple power, or the phase differences and amplitude? It's unclear how these were used to validate the electrode localizations using the MRID-tags.

We thank the reviewer for this helpful comment. Although these physiological landmarks are relatively well-known within the hippocampus field, the text was not readily accessible to the broader community. We have now clarified these throughout the Results section. Specifically, we used spontaneous sharp wave-ripple (SWR) events and theta oscillations as physiological landmarks with well-established laminar signatures in the hippocampus. By identifying these physiological landmarks along the electrode array and knowing the fixed 50 µm inter-contact spacing, we estimated the absolute position of recording sites. We then compared these estimates to the positions inferred from MRID-tag locations in MRI. The agreement between MR-based and physiology-based contact localization serves as a key validation of our anatomical targeting. To support this, we now include a Supplementary Figure showing the difference between MRID-based and functionally inferred positions for the PyL and hippocampal fissure, tracked across a 5.5-months period**.**

- Line 270: Should it be referring to Figure 4A?
  Yes, thank you. We have now modified the text to clearly reference Supplementary Figure 3 and Figure 4A, also improving the explanation of how these figures support electrode localization.

- Line 272: Could you please give a brief introduction to the 1D MRI-registered trajectory or topography? This will help people who are not familiar with MRI.
  We modified the manuscript to include a clearer explanation of the 1D MRI-registered trajectory. This refers to the mapping of the electrode path onto a standardized MRI atlas, in our case, the Waxholm Space (WHS) atlas. Once the trajectory is aligned within atlas space, we extract a one-dimensional profile of grayscale intensity values along the electrode path. These values reflect tissue contrast in the MRI image, where regions of higher cell density, such as the CA1 pyramidal layer, appear darker than surrounding tissue. By inverting the grayscale profile, a peak becomes visible at the location of the pyramidal layer. This approach provides a 1D anatomical topography along the electrode trajectory, which we use to localize electrode contact sites relative to known anatomical landmarks.

- Line 288-291: Do you refer here to the MRI- and Ephys-detected locations of the electrodes at the anatomical regions of PyL and HF? To make the sentence clearer, please add 'electrode locations' somewhere in the sentence. Additionally, how do you explain the high standard deviations? "n=3 bundles" across how many animals? or is it bundles within the same animal? Please comment.

  Yes, this section refers to the MRI- and electrophysiology-detected electrode locations within the PyL and HF. To address the reviewer's suggestion, we have added the term *"electrode locations"* to the sentence for clarity.

  Regarding the high standard deviations, these are within the expected range given the properties of theta oscillations. Theta is not a perfectly sinusoidal rhythm; its frequency and period vary across cycles and brain states, so cycle-to-cycle variability in phase is natural. The observed mean and SD values for the PyL-to-HF phase difference (~151° ± 20°) are consistent with previously reported phase gradients across CA1 layers (Lopes-Dos-Santos & Dupret, 2025).

  Concerning anatomical localization, the ~100 µm SD for MRI-based electrode placement corresponds to only ~1 voxel at our MRI resolution, which is acceptable even for registering electrodes to thin cortical laminae in small animal models.

  Finally, we clarify the sample size: data were obtained from n = 2 rats, with a total of n = 3 MRI-visible bundles used in this analysis.

- Line 293-294: How were these contact site locations determined? Due to the electrophysiological landmarks of the activity recorded or to the MRID-tags? or both?

Thank you. We now clarify that contact site locations in larger structures (CA1 vs. DG vs. M1) were assigned based on MRI, which was sufficient for this level of anatomical resolution and subsequently validated by hierarchical clustering of inter- vs. intra-areal cross-correlation analysis of the recorded neurons from MRI labelled contact sites (Figure 4F, Suppl. Fig. 4.1, 4.3). For finer localization within CA1 laminae, contact site positions were refined using specific electrophysiological signatures characteristic of different layers, as clarified in the revised manuscript.

- Line 297-298: Figure 4E, first column. How many electrodes within the shown bundle were accurately localized? Is it one electrode per unit (spike waveform) detected?

All MRID-visible bundles were successfully localized. For accuracy estimation, each neuron was assigned the anatomical label of the contact site from which it was recorded, based on MRID-tag registration. These anatomical labels were then compared with functional clusters obtained by hierarchical clustering, which revealed strong intra-areal correlations consistent with anatomical borders. The anatomical label corresponds to the contact site, and the spike waveforms of neurons recorded at that site inherit this anatomical assignment.

In the shown bundle, we analyzed **59 of 64 functional contact sites**. From these, a total of **54 neurons** were detected, of which **50 were correctly assigned**. **Depending on local density and recording quality, multiple neurons could be recorded from a single contact site.**

Each bundle consisted of 64 independent fibers, each carrying one contact site. The reviewer's use of "electrode" appears to refer to individual contact sites, whereas in our terminology, "electrode" refers to the entire bundle. The position of each contact site (individual fiber of a bundle) was estimated from the known 50 µm inter-contact spacing and electrophysiological markers at specific depths (e.g., ripple power maxima [0µm], theta phase gradients).

- Line 310: Please explain what "overall classification accuracy" means, how was it defined? How was it calculated? What is the accuracy of the classification or the localization yield of the electrodes across the samples? Could you do the same analysis as in Figure 4 for another bundle in a different animal and add it to the supplementary? This would show any differences between the samples.

We particularly appreciate this question, as our Methods section did not previously clarify how this classification accuracy was computed. We have now added a dedicated paragraph to describe this analysis in detail. We now define overall classification accuracy in the method section as the proportion of neurons whose MRI-based anatomical labels (assigned via MRID-tag localization and WHS-atlas registration) agree with the functional clusters obtained from hierarchical clustering of spike-train correlations: Accuracy = (number of neurons with matched anatomical labels) / (total number of neurons) × 100%.

$$Accuracy = \frac{N_{match}}{N_{total}} \times 100$$

For the dataset in Fig. 4, accuracy was 50/54 = 92.59% (M1: 10/13; CA1: 12/13; DG: 28/28); mismatches occurred at anatomical borders and are within single-voxel MRI resolution. As requested, we repeated the full analysis in a different animal/bundle with a slightly more lateral/posterior trajectory (new Supplementary Fig. 4.3). There, 39/46 neurons were correctly matched, yielding 84.78% accuracy (Thal1: 6/7; Thal2: 8/12; CA1, CA3: 25/27). Within the thalamus, two functional clusters emerged, consistent with its multi-nuclear organization; however, we did not attempt nucleus-level assignments. CA1 and CA3 form a well-known functionally connected subregion of the dorsal hippocampus, in line with established hippocampal circuit architecture. Accordingly, they exhibited high correlation and clustered as a functional unit.

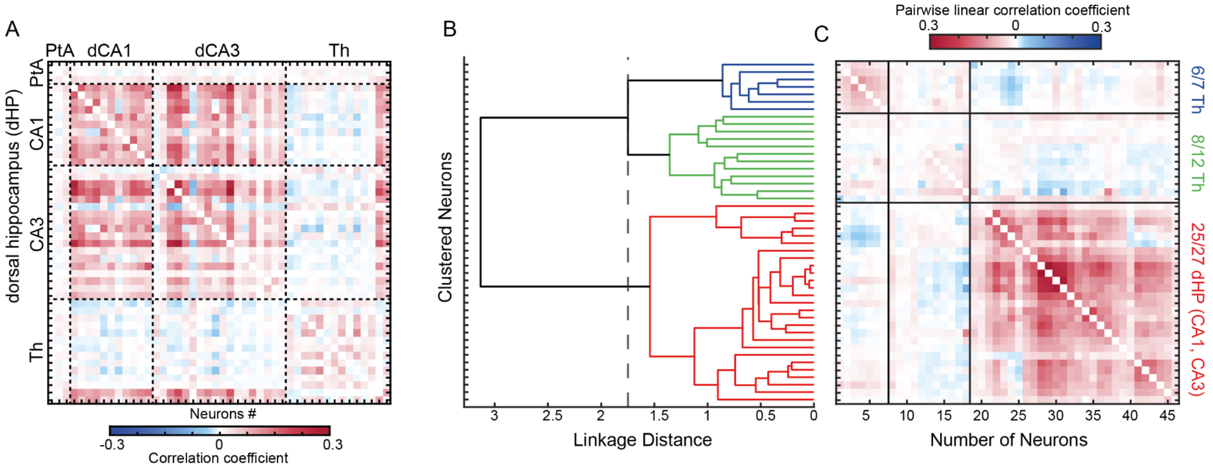

NEW Suppl. Fig. A. MRI- and function-based identification for a different bundle trajectory compared to Suppl. Fig. 4.1, with functional grouping determined by spike-train correlation and hierarchical clustering. Correlation matrix based on 25 ms binned z-scored firing rates. Neuronal IDs are derived from MRI-detected labels of contact sites (multiple neurons can originate from the same contact site). The plot preserves the dorso–ventral orientation of the brain. **B.** Hierarchical clustering of the correlation matrix. The dashed line indicates the threshold determined by the elbow method. **C**. Reordered correlation matrix according to the results of the hierarchical clustering. The hierarchical clustering revealed two thalamic clusters; units 6/7 and 8/12 were correctly labeled by MRI. In addition, 25/27 units from the dorsal CA1, identified as the third functional cluster, were correctly labeled according to the MRI localization.

Here we present an independent case of the hierarchical clustering results, as requested, and add to the main text -Results-. This figure is provided as Supplementary Figure 4.3. **A.** shows the correlation matrix; **B.** presents the hierarchical clustering that reveals functional relationships; and **C.** displays the reordered correlation matrix based on the linkage distance determined by the clustering. In this case, 6 + 8 + 25 neurons (contact sites localized by MRI) were accurately labeled out of a total of 7 + 12 + 27 neurons, resulting in an accuracy of 84.78% (39/46).

- Line 311-321: The paragraph reads as if a couple of bullet points were just strung together without any connection between them. Could you please rework the paragraph to make it clearer?

  We have revised the paragraph to improve clarity and logical flow. Rather than listing individual statistics as disconnected bullet points, we now present a cohesive summary of the results. The revised paragraph now explains the purpose of this analysis (i.e., to verify the MRI-assigned anatomical locations in CA1, DG, and M1) and how we used the activity differences among neurons recorded from MRI-labeled CA1, DG, and M1 sites.

- Line 322: Could you please comment and discuss the results of the electrolytic lesions observed during post-mortem histology? What did you find out? How does it correlate with the MRI scans and prediction of electrode positions?

  Thank you, we now clarified the purpose and the outcome of these post-mortem analyses in the Results and Discussion sections. The electrolytical lesion sites are consistent with MRI-estimated and electrophysiologically validated localization results.

- Line 349-355: What is the ratio of working electrodes across time?

  More than 95% of electrodes remained functional over a chronic period of 5 months (n = 3 electrode bundles, n=2 animals). We have added this information to the manuscript and included a supplementary figure showing bundle functionality over time (Suppl. Fig. 6.2). This figure also addresses the related question for Lines 361–365.

- Line 346: What happened to the third animal?

  The third animal (with n=4 electrode bundles) was implanted without any headposts and connectors for electrophysiology, to be used solely for MRI imaging and biocompatibility analysis. Because the cranial screws to fixate headposts, which allow us to carry out chronic recording experiments affect the MRI signal. However, we showed that we can still robustly detect MRID-tags and localize electrode channels in vivo in the vicinity of cranial screws, headpost, and peripheral connectors.

- Line 361-365: How many single- or multi-unit activity spikes were detected by each electrode, and how did this change over time? Did you notice any change in the position of electrodes over time? Perhaps you could provide a collage of MRI images at the same anatomical location in which a bundle is detected but at different points in time (e.g., monthly). How did the CoMs change over time?

  In total, we analyzed 256 contact sites from four bundles. Of these, 219 were functional (impedance 25kΩ–5 MΩ; bundle-wise counts: 59, 60, 50, and 46 contacts, with one bundle serving as a control without nanoparticles). From the functional contacts, we detected 122 single units and 72 multi-units *(see Methods for single- vs. multi-unit classification criteria).*

  To further illustrate recording stability, we generated a new supplementary figure (Suppl. Fig. 6) tracking electrode performance over time. Suppl. Fig. 6A shows (see below as well) the number of neurons recorded across days from all bundles (color code: green – bundle 1, blue – bundle 2, red – bundle 3, and control bundle 4 without IONP). Suppl. Fig. 6B shows the percentage of active channels across days. At baseline, all bundles began with 100% active channels (Bundle 1: 60; Bundle 2: 59; Bundle 3: 46; Bundle 4: 50), and across five months, more than ~95% of contact sites remained functional.

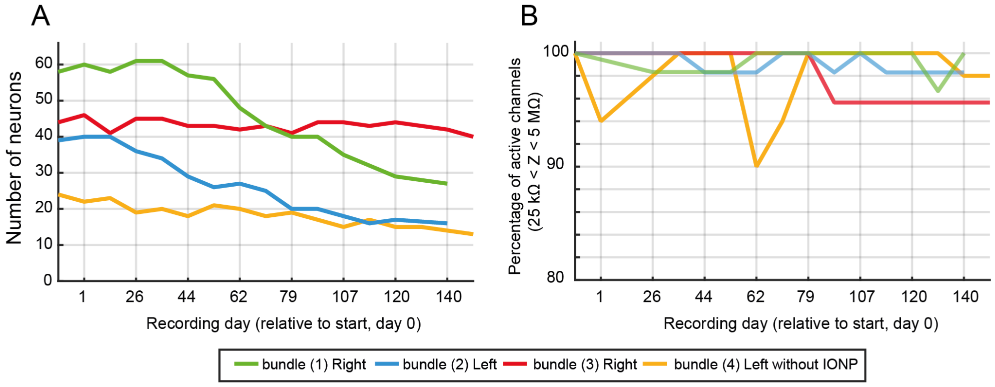

NEW Suppl. Fig. A. Number of neurons recorded across days from all bundles (color code: green – bundle 1, blue – bundle 2, red – bundle 3, and control bundle 4 without IONP). **B**. Percentage of active channels (25KΩ < Impedance (Z) < 5MΩ) across days. Bundle 1: initial 60 channels = 100% active; Bundle 2: 59 = 100%; Bundle 3: 46 = 100%; Bundle 4: 50 = 100%.

We did not observe systematic changes in electrode position over time. To illustrate this, we provide a **new** supplementary figure (Suppl. Fig. 6.1) comparing MRID-based electrode localization with electrophysiological landmarks derived from LFP recordings in the CA1 pyramidal layer (red) and the hippocampal fissure (blue). Solid lines indicate the right hemisphere and dashed lines the left. These bundles contained nanoparticles. Across recording sessions, MRID-based localization remained well aligned with electrophysiological markers, and the trajectories showed no major drift:

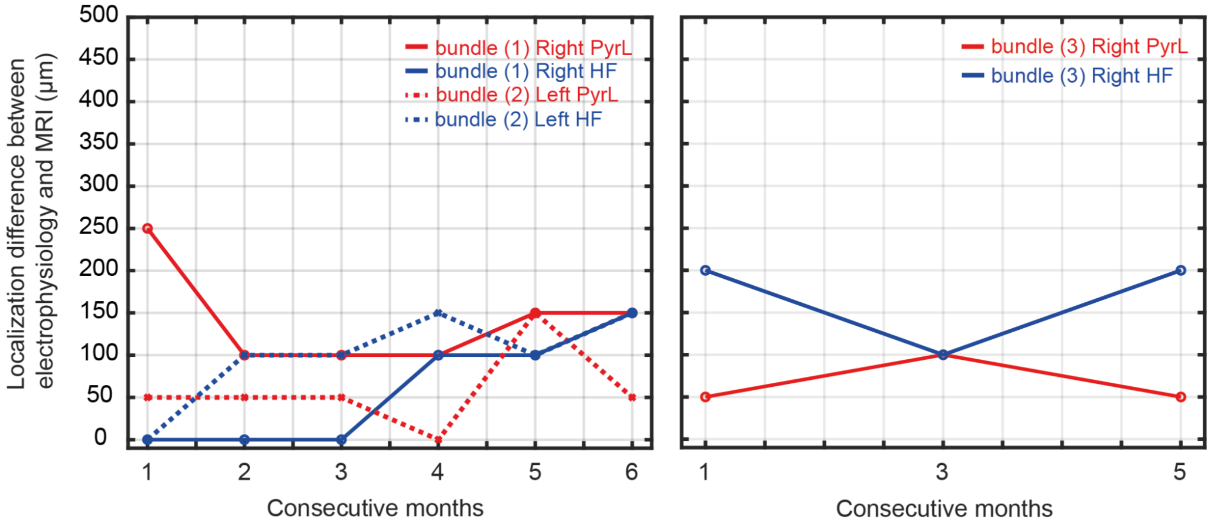

NEW Suppl. Fig. Localization differences between MRID electrode-based localization and electrophysiological landmarks derived from the LFP of the CA1 pyramidal layer (red) and the hippocampal fissure (blue). Solid lines indicate the right side, and dashed lines indicate the left. These bundles contained nanoparticles.

- Line 367-369: This sentence belongs to the Methods. Please check also the wording for clarity.
  Thanks. The sentence indeed belongs more to the Methods. We have now moved the corresponding text to the Methods and revised the wording.

- Line 372-373: If there's a significant decrease in impedance, it could mean that the electrodes are failing. What was the ratio of functional electrodes over time?

  Electrode impedances remained within the functional range (25 kΩ < Z < 5 MΩ - see previous response) throughout the recordings. We define this range as follows: channels with impedances below ~25 kΩ are likely short-circuited (e.g. delaminated) or otherwise non-functional, while channels with impedances above ~5 MΩ typically have poor signal quality and fail to detect spiking activity reliably. Using this criterion, more than 95% of electrode channels remained functional over time.

- Line 376-379: It seems that this sentence belongs to the Methods, what is the main takeaway here? Why did you slice the trajectory horizontally?
  We have now reworked the section, corrected this sentence, and moved it to the Methods section.

  We used this experiment to calculate/show the stability of IONP coating. We coated a bundle uniformly with a known amount of IONP and we quantified the IONP contrast using horizontal sections.

- Line 386-392: If the trajectory was sliced differently, is the MRID contrast comparison between Animal#1 and Animals#2-3 equivalent? From Animal#1 it seems hard to distinguish the spatial distribution of the bundle, I would've expected to see a more enlarged or circular MRI contrast profile, could you please comment how is the imaging slicing orientation affecting the localization of the MRID-tags?
  For the chronic contrast comparison, what matters is that the imaging slice encapsulates the same segment of MRID-tag at the initial and final MRI sessions in the same animal. In Figure 5B, we do not compare the contrast intensities across different MRID-tags, but we compare within each MRID-tag across time. Therefore, the slicing orientation does not affect the contrast comparison.

For an accurate localization, we use at least two different imaging slicing orientations (e.g. coronal and sagittal) to truly capture the 3D trajectory of MRID-tags in the brain. Depending on the complexity of electrode bundle trajectory multiple imaging slices can be used and merged for a better localization accuracy.

We now further clarified these in the manuscript.

- Line 396: typo? "...to image neurons".
  Thanks, we now fixed this.

- Line 399-401: Could you please provide an overview of all 13 slices in the supplementary? Why is the implant red? How is the footprint (cross-sectional area) of the implant changing across depths?
  We have now provided all the 13 slices in the Supplementary Materials.

  Electrode fibers and neurons (Nissl) were imaged with the same imaging channel. To make them visually differentable, we assigned the implant red color and neurons blue. It is purely post-processing, and for illustrative purposes. The immunohistology fluorescence analysis was carried out on the raw images.

  The footprint of the implant gets smaller towards deeper segments as the number of electrode fibers are less at deeper segments. In the figure of the main text, we show a representative segment with the largest footprint (including all fibers). We now clarified these in the figure caption.

  Please find below the overview of all 13 slices for your convenience.

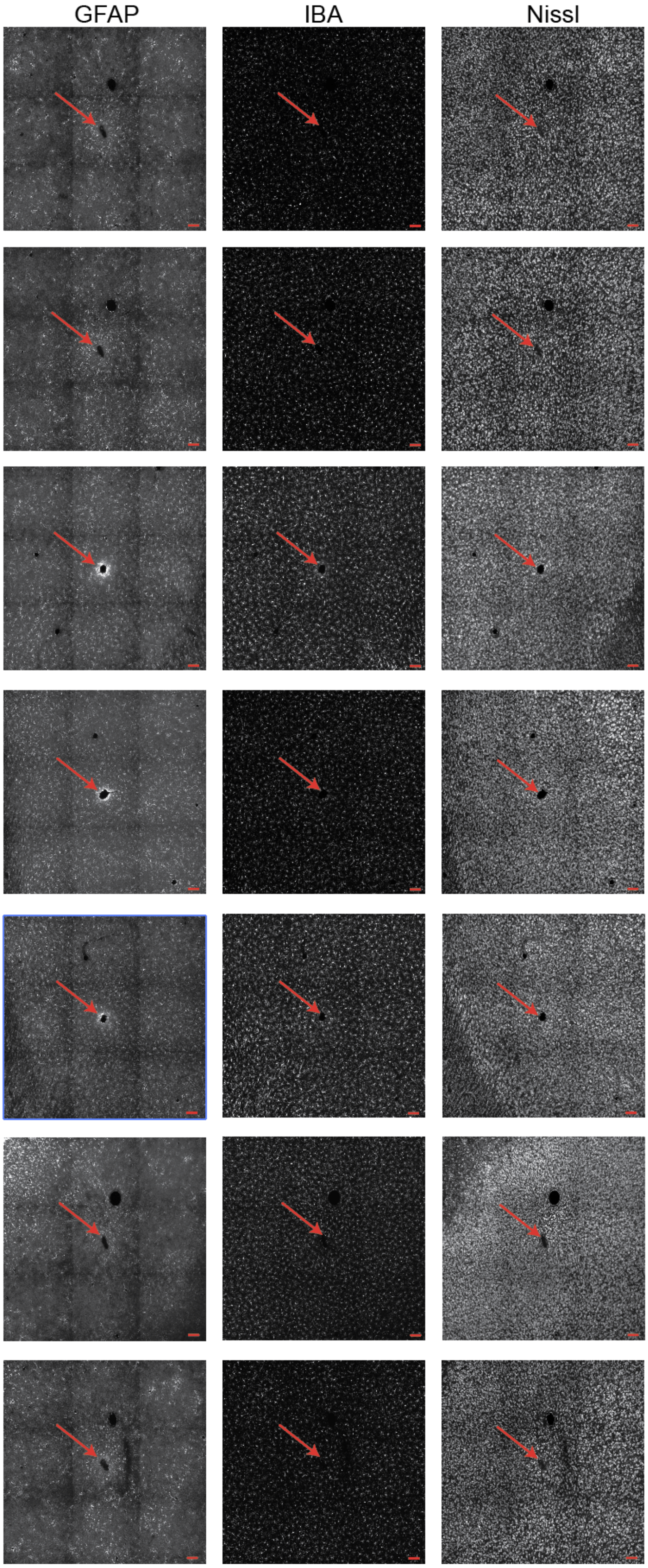
GFAP
IBA
Nissl

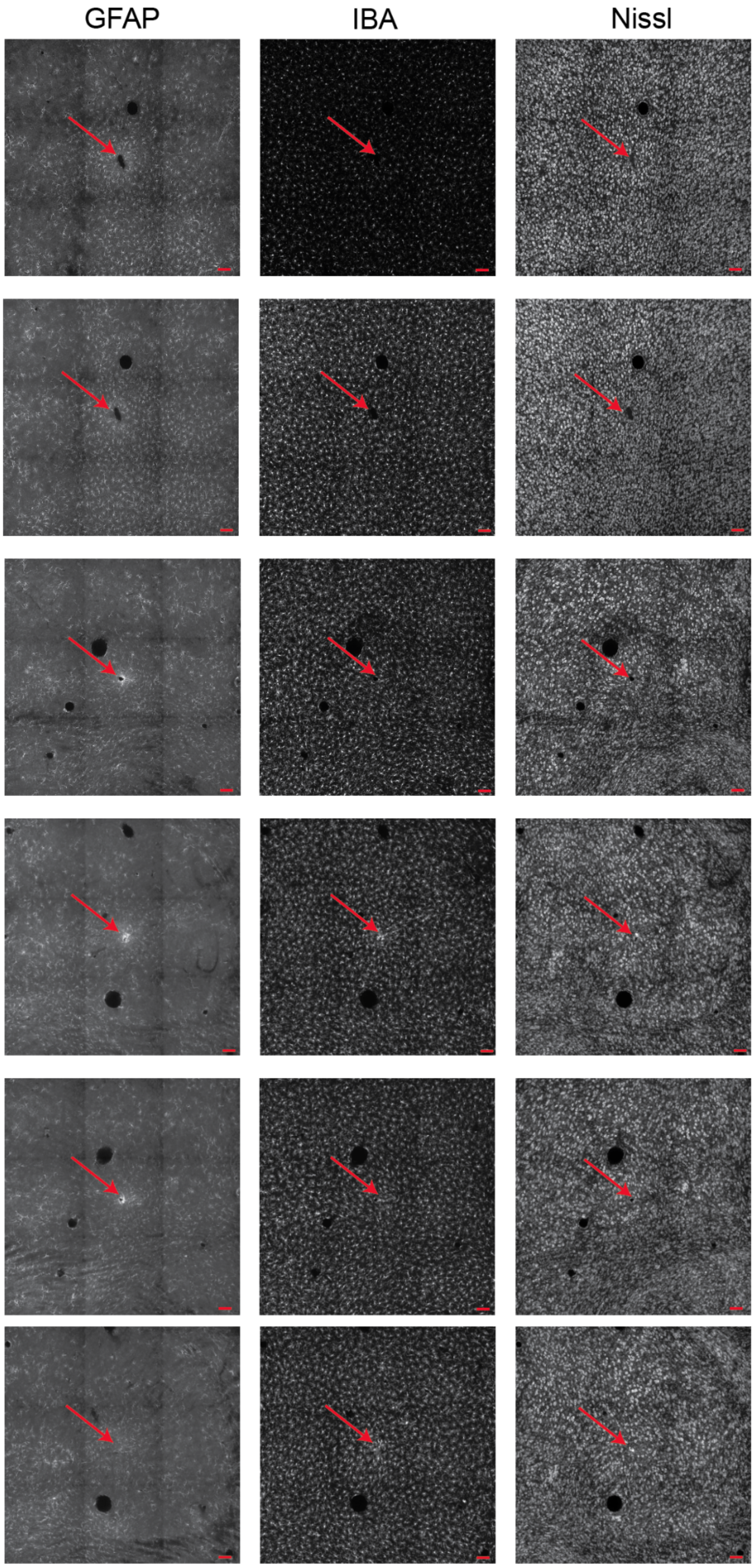

NEW Supp. Fig. All histology images with splitted imaging channels (Nissl, IBA, GFAP) are shown. Red arrows point to the UFTE bundle positions. Scale bars denote 50 μm.

- Line 404-409: How do these results compare to the literature? or to your previous publication without the MIRD-tags?
The results are qualitatively comparable to the literature (e.g. Lycke et al 2023) as well as to our previous work (without the MRID-tags, Baran et. al. 2024).

- Line 443-445: To which structure do you refer here?
Here, we refer to the structures on the sample such as gold traces etc. We have now clarified the corresponding sentence for better readability.

- Line 445-446: In which cases?
We now clarified this: A potential case would be using MRID-tags in low field MRI scanners where the image resolution and SNR are substantially lower than the 7T or 3T scanners we used in this work.

- Line 446-447: Do you mean an intermediate layer of IONPs encapsulated with PI? Is that the IONPs would be directly on PI and not anymore on the Au traces of the electrodes? Why would it doubled?
Yes, just as an alternative, we meant a 3-layer Polyimide sandwich where there are 2 layers of IONPs. This would mean IONPs would be patterned separately on both the middle PI and the Au layers. Please note that another potential alternative, a single much deeper trap (with higher aspect ratios) would be problematic because they tend to trap air pockets during the nanoparticle assembly.

- Line 451: Replace center of masses for CoM.
We now replaced these.

- Line 460: Please introduce the abbreviation PEEK when first used in the text.
We now introduced the abbreviation “PEEK” (Polyether Ether Ketone) at its first mention in the manuscript, as requested.

- Line 464-468: Please discuss what is the smallest INOP-coated feature size that can generate a measurable MRI contrast. This part of the discussion should be included in the Results and Discussion section.
According to our measurements, the smallest IONP-coated features should be at least 2 MRI-pixels long (~270um), where the density of >50 IONP dots per pixel is preferable (Fig. 3e). We now mention this in the manuscript.

- Line 483: What is the expected dissolution rate of silk fibroin? Is it expected to be longer than one month?
The expected dissolution rate of silk fibroin is a few days. We now carried out further experiments for the dissolution rate of silk fibroin, also confirming that the complete dissolution is within a few days. We now clarified this in the manuscript. Impedances gradually decrease during the early days of implantation, likely due to the dissolution of silk fibroin.
For the dissolution rate of silk-fibroin in protease solution, please also see the figure related to the sterilization of electrode bundles.

- Line 485-486: According to Figure 5A, impedances were stable for 53 days, decreased at day 59, were stable again until day 83, decreased again, and had a prominent decrease at day 110. However, such impedance fluctuation seemed not have affected the quality of the recordings, as SNRs even increased over time. How do you explain this? Is there any fluctuation in the electrode position? As questioned before, what was the ratio of functional electrodes?
We do not observe significant changes in the electrode positions neither in electrophysiology nor in MRI.

Since the silk fibroin should be completely resolved after a few days, we suspect that these changes could be due to transient microglial movements around the electrodes (where microglia roams even in the healthy brain) or due to the movement of interstitial tissue (conductive) around the electrodes (potentially due to a physical trauma in the home cage). We note that the transient impedances never drop below the in vitro levels (i.e. ~50kOhm), indicating that these are not likely due to the unlikely transient delamination of the polyimide cables etc. We now mention these in the manuscript.

- Line 492-494: The connection of electrode localization with such clinical applications was not made before. Can the authors elaborate more? CT is also used in those applications, is this method restricted to MRI?
  Although our method is not strictly restricted to MRI, it harnesses the superparamagnetic properties of iron-oxide nanoparticles—which provide strong contrast with minimal material—and temporal dynamics of MRI images to accurately reconstruct electrode shank trajectory and localize the individual electrode channels.
  We have now reworked the corresponding paragraph in Discussion.

- Line 492: Any electrode going inside the brain is always going to be invasive. Please reformulate the sentence.

  We corrected this as ‘minimally invasive”. We agree with the reviewer that all penetrating electrodes are, by definition, invasive. Ultra-flexible neural implants are designed to minimize such chronic tissue response and mechanical mismatch, thereby reducing long-term damage compared to conventional stiff electrodes.

- Line 494-496: What kind of “precisely targeted applications”? Is the localization of electrode clusters enough, or is the localization of individual electrodes needed when using imaging methods in combination with electrophysiological methods? Perhaps it was not clear, but only the localization of electrode clusters was demonstrated, and the localization limits of high-density arrays were not explored. How closely packed can the electrodes be to localize them accurately?

  We now clarified this in the manuscript. We indeed showed here we can determine the positions of every electrode by our approach using two steps: Within each electrode bundle/array, the inter-electrode distances are approximately determined by the layout of bundle fibers. **MRID-barcodes allow precision localization of the identities and the positions of the bundles within the brain, after which the inter-electrode distances from layout can be used to identify the electrode positions with high precision (even when the bundle consists of mechanically independent electrode fibers as we validated in this manuscript using extensive electrophysiology)**.

  Potential clinical applications could benefit from the use of both the bundle identity/position as well as the precision electrode position information, using our technology.

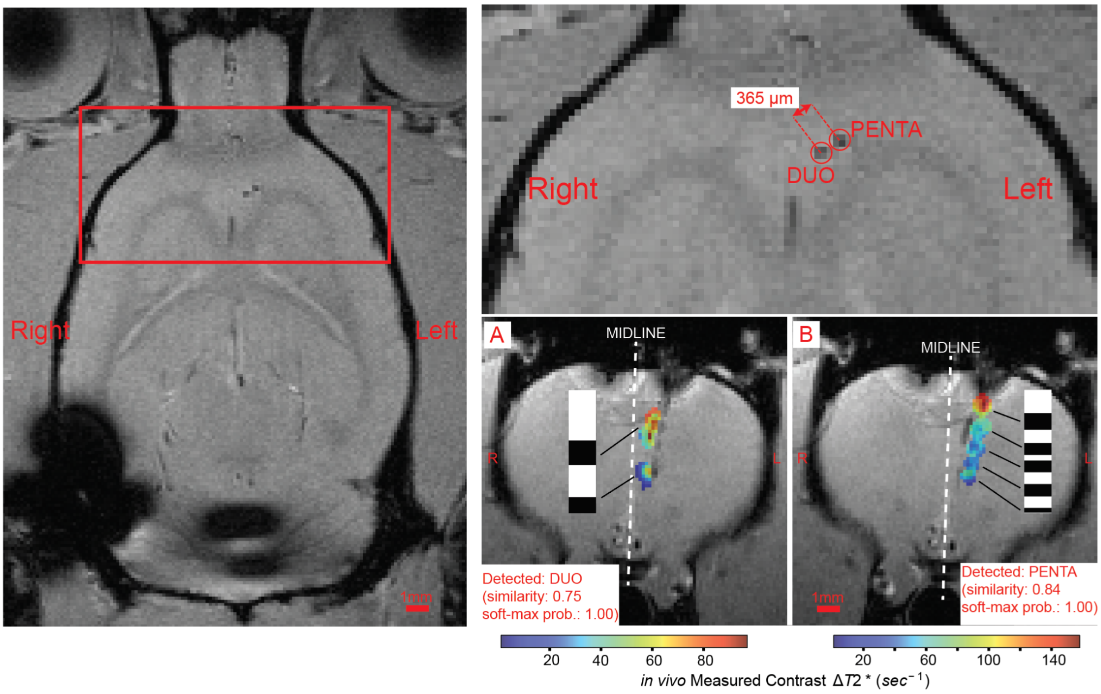

NEW Rebuttal Figure. Two MRID-tagged UFTE bundles implanted in close vicinity (365 um separation). The left panel shows the horizontal MRI image, and the top-right panel shows the zoomed-in image of UFTE bundle cross-sections. Panels A and B show the MRI-barcodes from each bundle. Insets in panels show the detected barcodes with similarity and probability scores.

- Line 496-498: What is still missing so that this technology can be employed in NHP or human applications? Please comment on, e.g., sterilization methods, front-end connectors, etc.
  We now included the clinical-grade sterilisation method and the data we validated for MRID-tagged UFTEs.

  There are many different types of front-end connectors, which can be easily incorporated with standard CAD/PCB design techniques, which falls out of the scope of this manuscript.

Figures:

- Figure 1 should be referred to in the Results section and not directly from the introduction.
  Since we believe that Figure 1 is a very high-level overview and is very helpful to understand the content/innovation in the manuscript, we now moved it to the transition between the Introduction and Results sections.

- Figure 1B: It is not representative of an implanted device in a rat brain, but rather a schematic of the insertion method; please revise its reference in line 68.
  We now revised the wording according to the reviewer comments.

- Suppl. Fig. 1A is not referred in the text.
  We now refer to this figure in the main text.

- Figure 2E: Is the scale bar needed? If so, what are its dimensions?
  We now added the dimensions on the scale bar in Figure 2E.

- Figure 3: Add labels to individual sections of each panel. E.g, Ai, Aii, or any other label to distinguish them.
  We now added labels for individual sections of the panels.

- Figure 3: What is the difference between CoMs and curve centers? How do they relate? Please expand the explanation in the main text.

We now clarified. We calculate the CoMs using the IONP coating amount and dimensions from CAD designs. We measure and detect gaussian curve centers from the MRI images, which are located at the CoMs as we validated by our characterization experiments and results.

- Fig. 3A: What is μ2 or μ1, the mean of the Gaussian distribution? What is W1 and W2? Please define the variables in the figure caption and the main text.
  We now further clarified these variables both in the figure caption and main text.
  μ1 and μ2 indicate the center point of the 2D gaussian curve.
  W1 and W2 are the detected/measured width of the bars in the reconstructed MRID-barcode.

- Suppl. Fig. 3: Please add an order to the data processing and analysis pipeline; it's overwhelming to see the schematic, and it's unclear where to start reading.
  We now included a better illustration of the data processing and analysis pipeline.

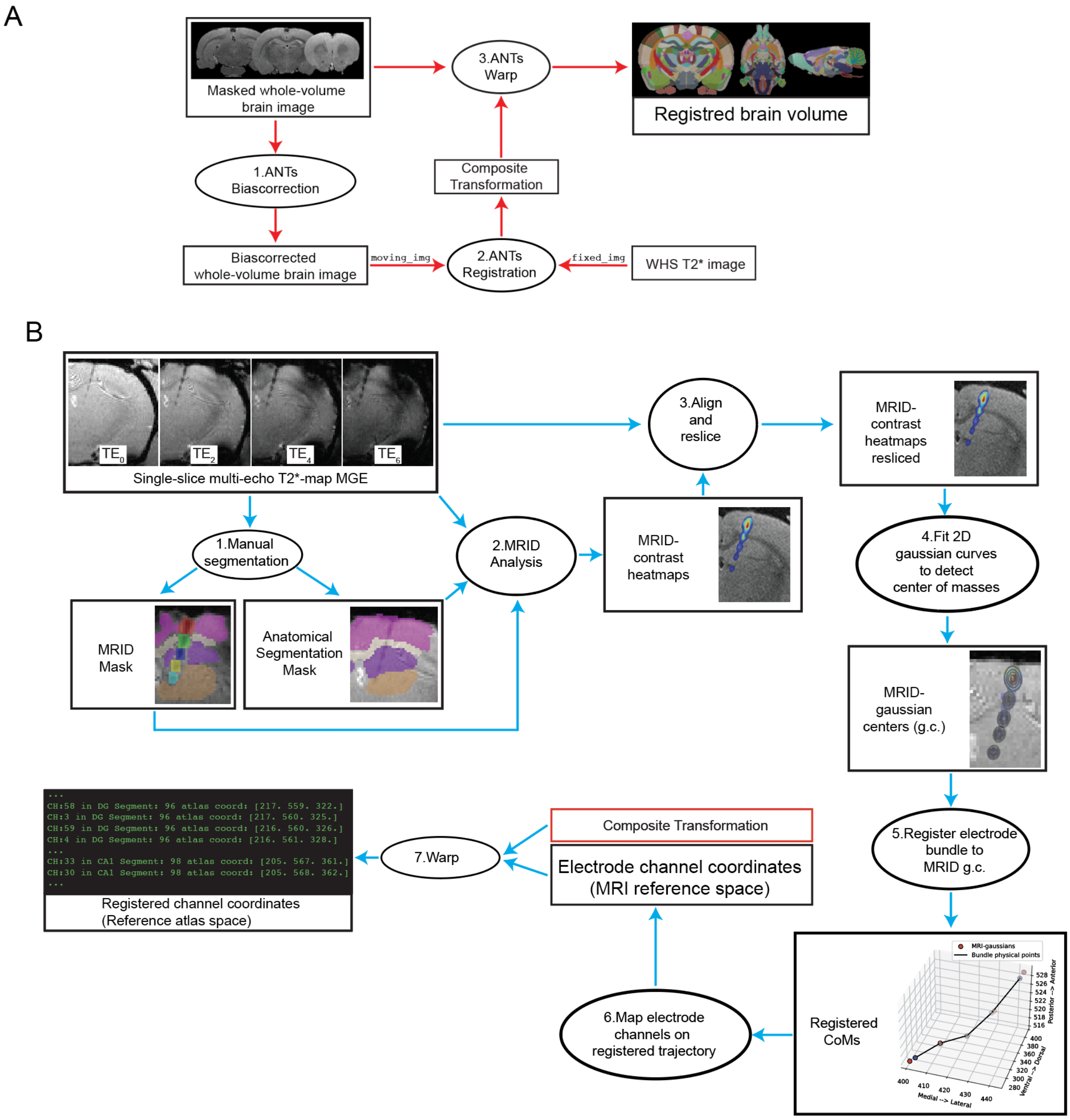

NEW Suppl. Fig. 9. MRI image registration and MRID-tag image analysis pipeline explained. **A.** Whole-volume brain MR images are biascorrected and registered to WHS reference atlas outputting a composite (i.e. rigid, affine and elastic transformations combined) transformation. **B.** Single-slice multi echo time T2*-map MGE images of MRID-tags are analyzed with custom MRID analysis pipeline. Images are manual segmented to define region of interests for MRID-tags and anatomy. Contrast heatmaps are generated for each MRID tag and Gaussian curves

are fit around the heatmaps which estimates the CoMs of IONP islands. Point-set registration finds the best fit bundle trajectory explaining the MRID-tag induced contrast in MRI. Electrode channels are mapped on the best fit bundle trajectory using the exact design layout dimensions. Composite transform from reference atlas registration is used to map each electrode channel into reference atlas space to identify the anatomical location

- Figure 4B: What are the units of the x axis?
  We now updated the figure caption to clarify that the x-axis represents arbitrary units corresponding to normalized, inverted grayscale values from the 1D MRI topography. [The x-axis represents inverted grayscale intensity values extracted from the MRI image along the 1D trajectory of the electrode. These values are unitless [a.u.] and have been normalized between 0 and 1 to reflect relative tissue contrast.]

- Figure. 4C: The figure caption says the first column shows raw SWR, however, it's unclear if raw means all frequencies (e.g., up to 3-5 kHz) or just the frequency range in which SWR are encountered (e.g., below 300 Hz)?
  We now clarified in the figure legend that this is the raw signal, where spike waveforms are visible, covering the full bandwidth from 0.1 Hz to 7.5 kHz (default by Intan tech: https://intantech.com).

- Figure 4A and 4C, second column: Where is the color code and the corresponding units of the heatmaps?
  We now added the corresponding color and units to the figure legend for the heatmaps shown in the second columns of Figures 4A and 4C. The unit is visible at the bottom left corner of Figure 4C, and it is true for all channels.

- Figure 4: Label F is missing in the figure caption.
  We now added F to the figure legend.

- Figure 4D: Is there a significant difference between the average localization difference across anatomical landmarks, e.g., PyL and HF? Is there an influence of depth on localization accuracy? Check possible typo, is it PyrL or PyL?

  We thank the reviewer for these insightful comments. Our primary goal in Figure 4D was not to compare localization accuracy between PyL and HF directly, but to evaluate how accurately electrode locations can be determined with respect to each anatomical landmark individually. This was intended to demonstrate that MRI-based localization can be trusted—even in the absence of clear electrophysiological markers. We agree that the potential influence of depth on localization accuracy is an important consideration. The result showed that the difference between the two was not statistically significant t-test ($t(22) = 0.79$, $p = 0.44$, pooled SD = 64.76 μm), indicating similar localization accuracy across these structures (we now add this comparison to the result). We corrected the typo in the figure label from "PyrL" to "PyL" in the revised version.

- Figure 4: Please add scale bars in MRI images. (Missing from FigA D)
  We now added scale bars in MRI images in the corresponding Figure.

- Figure 5B: It would be better to show just one animal, but with more data points over time (e.g., monthly). The data for the other animals should then be included in the supplementary. This would help to better observe how the MRI contrast is changing over time.
  We now revised the Figure showing only n=1 animal and n=1 bundle over time. We also added other examples from other animals and bundles in the Supplementary Materials.

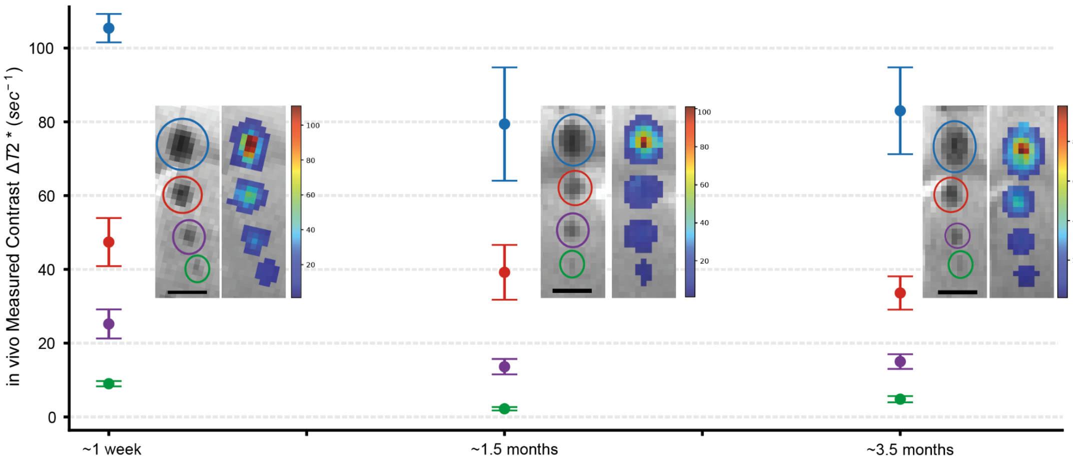

REVISED Figure 5B. Chronic in vivo induced MRI contrasts over 3.5 months of a sample bundle. Mean and s.t.d contrast intensities (5 most contrastive pixels) from each IONP island is plotted at 1-week, 1.5 months, and 3.5 months post-implantation (blue: IONP island #4, red: IONP island #3, purple: IONP island #2, green: IONP island #1), scale bar denotes 1 mm.

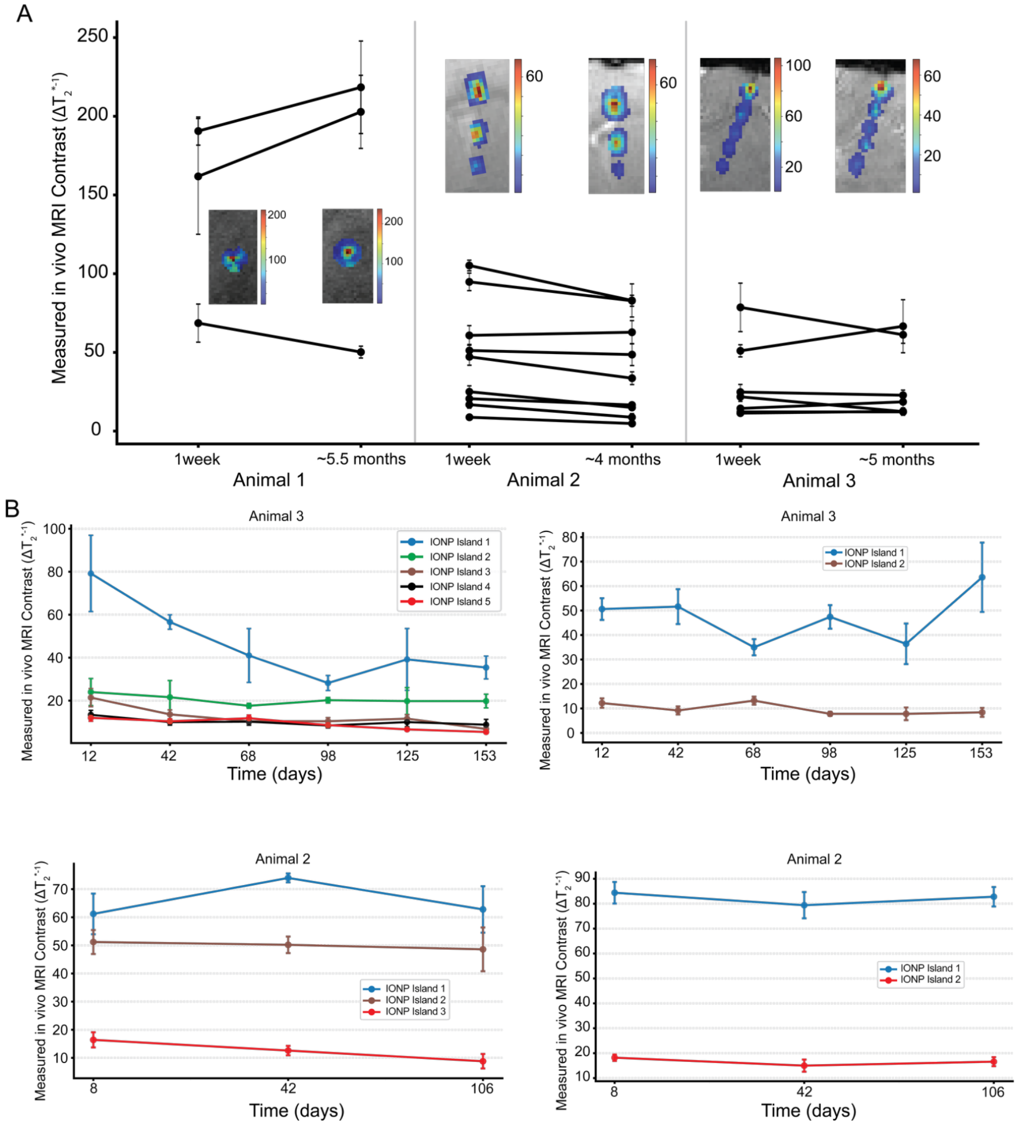

NEW Suppl. Fig. Chronic MRI contrast induced by MRID-tags. A. Post-recovery and prior to perfusion MRID-tag induced contrasts from all animals (mean ± standard deviation, *n* = 5 pixels per point). **B.** Chronological MRI contrasts from four example MRID-tagged UFTE bundles from two animals shown.

- Figure 5C: Please add the scale bars to the stainings.
  We now added scale bars to the images and made the circular ROI boundaries more visible.

- Suppl. Fig. 5: What is the thickness and distance between the slices? Please add scale bars and label of the different anatomical regions/layers for clarity. Such pictures should probably be better placed on the front page in the main manuscript, not in the supplementary, as they confirm the actual location of the implants.
  We now clarified in the main manuscript. The thickness of the slices are 100 micrometers. We picked the 3 consecutive slices along the anterior-posterior axis. We kept the figure in the Supplementary section because our quantified validation analysis relies solely on electrophysiology data, whereas the comparison of MRI images with post-mortem histology results is only qualitative.

  We also added another example to the corresponding supplementary figure to show the variance.

Methods:

- **Microfabrication of the ultra-flexible electrodes and MRID-tags:**

1. Did you explore other surface activation methods before considering roughening the Au surface with wet etching methods? For example, oxygen plasma or other surface coatings?
   Since roughening Au surfaced worked very well, we did not try other surface activation methods in this work such as oxygen plasma.

2. Is there an adhesion promoter employed before spin-coating the second PI layer?
   No adhesion promoter was employed before spin-coating the second PI layer. The procedure is the same as in our previous published work Yasar et al. 2023 Nat Comm.

3. Please provide details of the PEDOT:PSS blend. What is the concentration of PH100 versus EG, DBSA, and GOPS?
   We now referenced that the exact mixture rates of PEDOT:PSS blend given in our published work [Yasar et al. 2023 Nat Comm.] and the original work [Sessolo, M Adv. Mater. 2013].

- **Assembly of the UFTEs**

1. What kind of solder paste and method was used to bond the flexible implant onto the flexible PCB?
   We now clarified that a standard solder is used to solder ultra-flexible electrodes to the flexible PCBs with a standard soldering iron.

2. Curing temperatures and duration of medical-grade epoxy are missing.
   We now clarified that the medical-grade epoxy is cured at room temperature between 48h-72h.

3. Why was a two-electrode cell configuration used for impedance measurements? What equipment was used to measure impedances?
   We used Intan RHD Recording System (for both pre-surgery *in vitro* and *in vivo* impedances) for impedance measurements. The system operates in two-electrode configuration with a working-electrode (single Au/PEDOT:PSS electrode of UFTE) and reference-electrode. We now clarified that the reference-electrode is:
   - Ag|AgCl wire for *in vitro* impedance measurements
   - Au sputtered Titanium medical grade cranial screw for *in vivo* impedance measurements

4. What was the total weight of the assembly?
   We now clarified that the total weight of the assembly is 35.3 grams.

- **Surgical procedures:**

1. What was the dose of the analgesic Meloxicam?
   We now clarified that 2 mg/kg of body weight Meloxicam was used.

2. It is mentioned that Ringer's solution and glucose were injected s.c. at fixed intervals. How often were these intervals, and what volume per weight was used?
   We now clarified that we injected the pre-warmed isotonic glucose solution up to 5 ml s.c. for initial injection and every 4 hours up to 2ml s.c. during the surgery.

3. Did you monitor the temperature of the animal during the surgery?
   We now clarified that we monitor the body temperature of the animal with a rectal probe which is also connected to a closed-loop heating pad that is placed underneath the animal.

4. How did the animals recover? Was an additional analgesic given?
   We now clarified that the animals recovered single housed with tramadol drinking water (0.25mg/ml) readily available in the cage and 2 mg/kg per body weight Meloxicam administered twice a day.

- **Imaging rat brain in vivo in MRI**

1. Was the first scan one month post-implantation?
   We now clarified that the first scan is between 10-14 days post-implantation.

2. What was the concentration of isoflurane used for inducing the animals?
   We now clarified that the 4% Isoflurane in 1lt/min O2 flow was used for inducing the animals.

3. Did you monitor the temperature of the animal?
   We now clarified that we monitored the temperature of the animal with a rectal thermometer while a circulating-water heatpad was also used to maintain the body temperature.

4. Could provide pictures of the experimental setup during MRI scans. In line 631, it gives a reference to the "Supplementary", however, no reference is given, and none of the supplementary Figures is related to it.
   Suppl. Fig 2Ei provides a picture of the setup during MRI scan. We have now added labels to the individual subpanels in panels too.
   Please also find below an example photo of the experimental setup for MRI scan for your convenience.

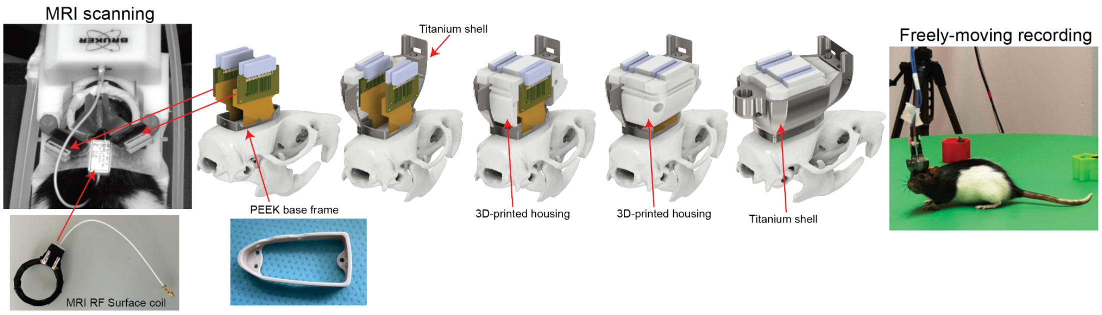

REVISED Suppl. Fig. Modular 3D-printed housing with titaniumHelmet for chronic MRI scanning and freely-moving awake recordings. Left-most side shows the MRI scanning setup. 30mm surface receive-only ring coil is used. PEEK base frame is used to fixate the housing to the skull.

- **MRID analysis:**

1. Line 674: "ri denotes the relaxivity constant of the constant (where i: 1,2,2*)": Which constant? What is its value? What is i?

There was a typo in the text which we now addressed in the manuscript. The sentence should read as “ri denotes the relaxivity constant of the contrast agent (where i:1,2,2*)”

Here, r_i is in $mM^{-1}.s^{-1}$ for T_i-weighted imaging is the imaging modality (i: 1,2,2*). Relaxivity constant of an MRI contrast agent is determined by measuring the relaxation of different concentrations of the MRI contrast agent in water.

2. Please specify that [CM] in Eq. 1 is the concentration of the contrast medium. Which units are used?
   We now clarified that the unit is $mM^{-1}.s^{-1}$.

3. What were the observed concentrations?
   We now clarified that the mean observed concentration was 0.035 mM for the most dense MRID-tag region, while the observed concentration as low as 0.005 mM was still detectable.

   Please see below how we compute the relaxation constant of the IONP which we used. We now added this to Supplementary Material.

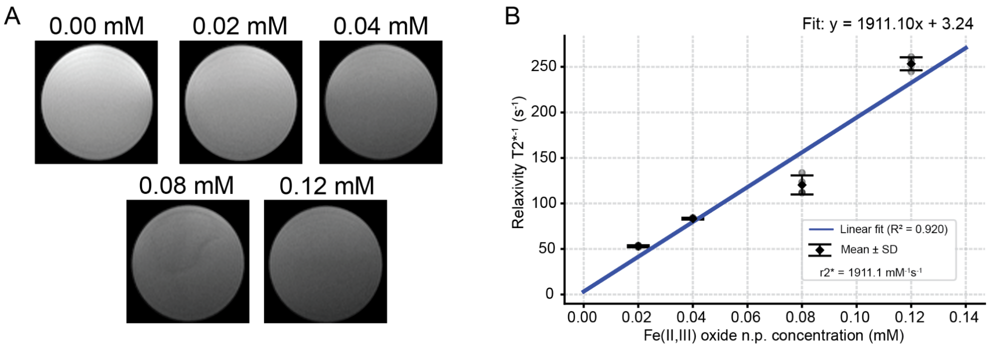

NEW Suppl. Fig. 4. Characterization of induced contrast by IONP in 7T MRI at varying concentrations. **A.** T2*-MGE (TE: 4.09ms) MRI images of phantoms at varying IONP in dH_2O concentrations. **B.** T2* relaxivity of IONP (25 nm avg. diameter). Mean measurements are shown with **black** scatter plots (n=4 MRI imaging slice at each concentration). **Blue** line shows the best fit giving the relaxivity constant (i.e. r2*) of IONP.

4. It would give more clarity if the authors could give a full example on how the different equations were used, giving numbers to the variables in the equations, and correlating it with the images used. As mentioned before Suppl. Fig. 3 needs improvement, and the order in which the analysis is carried out is lost.
   We now reworked the structure of Methods Section alongside with the Suppl. Fig 3.

- **In vivo recordings from rats**

1. What kind of filters were applied to the raw data? E.g., Butterworth, Notch, etc?
   We now clarified that the in vivo recordings from rats were acquired with a bandwidth of 0.1 Hz to 7.5 kHz. During acquisition, a hardware high-pass filter at 0.1 Hz and an anti-aliasing low-pass filter at 7.5 kHz were applied.

- **Local Field Potential Data Processing**

1. Why were LFPs down-sampled?

   We down-sampled the LFPs after applying appropriate low-pass filtering because the electrophysiological markers we investigated, including theta (~7 Hz) and SWR components (<200 Hz), lie well below 300 Hz. According to the Nyquist–Shannon sampling theorem, the sampling rate must be at least twice the highest frequency of interest; thus, a rate of 2,000 Hz is more than sufficient to capture signals up to 200 Hz without loss of information providing 10 sample points per cycle at 200 Hz. A second consideration was data size: a 1-hour session recorded at 20 kHz with 128 channels and 16-bit resolution produces about 17.2 GB of raw data, whereas down-sampling to 2 kHz reduces this to about 1.7 GB, making

storage and analysis substantially more efficient when handling multiple sessions. This is a standard procedure in electrophysiological investigations of LFPs from extracellular recordings.

2. Line 821-822: What is the normalized amplitude?
   We now clarified that the normalized amplitude (theta amplitude) is the instantaneous amplitude of the signal obtained from the Hilbert transform, divided by the maximum amplitude value across the entire signal. This results in an amplitude signal where the maximum value is 1, and all other values are scaled proportionally between 0 and 1.

- Adding diagram flows in the Supplementary to show the different offline data processing pipelines used in the electrophysiological data will give clarity to the reader.

  In addition, we have included workflow diagrams in the Supplementary Information (Suppl.Fig.4.4) to illustrate the different offline data processing pipelines used in the electrophysiological analysis, which we believe improves clarity for the reader. (see fig. Below as well:)

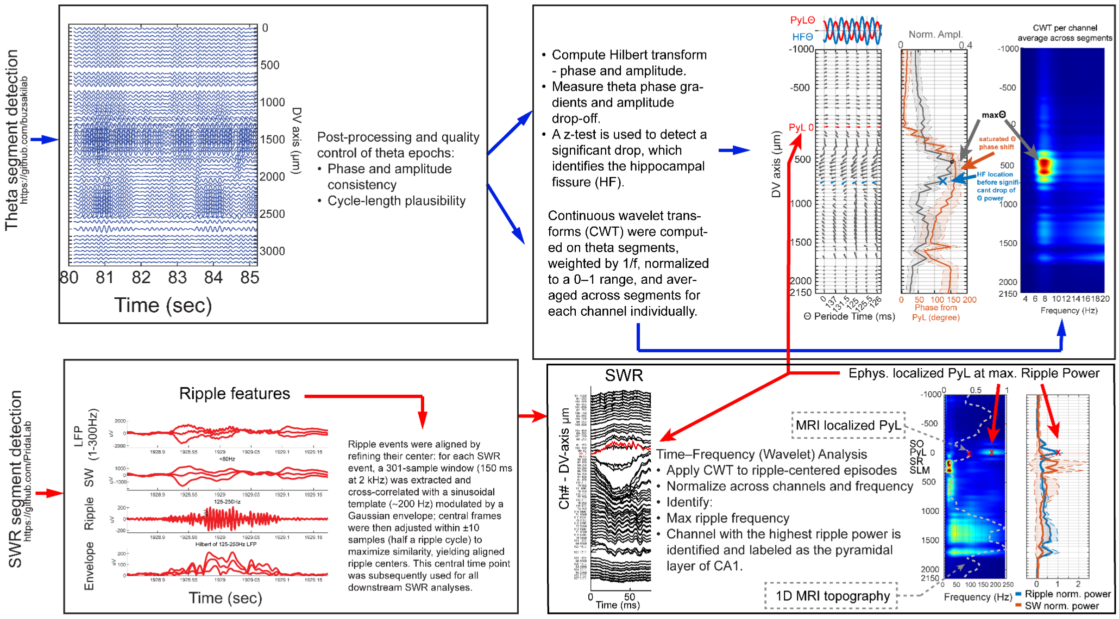

Suppl. Fig. 6.4. Workflow diagram illustrating electrophysiological localization of the pyramidal layer (PyL) of dorsal hippocampal CA1 and the hippocampal fissure (HF).

The first row shows the workflow for HF localization, beginning with detection of the theta segment, followed by identification of the maximum theta power using the Hilbert transform of all channels along the dorso–ventral axis (grey arrow in the right panel, maximum theta in SLM). From this point, the algorithm iteratively determines the significant drop in theta power (blue arrow) together with the saturation of the theta phase shift (orange arrow) relative to the cycle zero (peak) of theta, recorded from the PyL. The second row illustrates the localization of the CA1 PyL in the dorsal hippocampus based on SWR events. The algorithm searches for the maximum ripple power along the dorso–ventral axis; once identified, the corresponding channel ID is passed to the HF detection algorithm to be used as the reference channel (0 μm) for phase difference calculations and as the starting point for detecting the drop in theta amplitude toward the dorsal direction from the PyL. Dashed grey frames and arrows indicate the corresponding points of MRI-based localization (1D MRI topography; see Methods), obtained independently by the MRI localization algorithm. Dashed grey frames and arrows indicate the corresponding points of MRI-based localization (1D MRI topography; see Methods), obtained independently by the MRI localization algorithm. Note that HF MRI localization is not labeled in the workflow, as it comes directly from the MRI localization algorithm. Finally, the difference between MRI- and electrophysiology-based localization (HF and PyL) along the dorso–ventral axis is calculated to assess accuracy (see Results).

Abbreviations: SWR – Sharp-wave Ripple, SW – Sharp-wave component of SWR (~20Hz), SO – stratum oriens; PyL – pyramidal layer; SR – stratum radiatum; SLM – stratum lacunosum-moleculare; HF – hippocampal fissure.

- **Electrolytical Lesioning:**

1. Can you provide references on this lesioning method.
   We now clarified the references for this lesioning method and parameters used for UFTEs.

2. How did you confirm the lesion in vivo and post-mortem?
   We now clarified that we did the lesioning briefly before the perfusion. We confirmed the lesion with post-mortem immunostaining.

3. Line 881: Explain what "for each polarity" means. Do you mean biphasic current pulses containing a cathodic and an anodic phase?
   We now clarified that 'each polarity' means the polarity of the DC current applied. We first applied a positive DC current pulse for 5 seconds. We then applied a negative DC current pulse for 5 seconds.

- **Immunostaining:**

1. Were the rats perfused?
   We now clarified that the rats were perfused with PBS and PFA.

2. Was the brain fixed with PFA or any similar?
   We now clarified that we fixed the brains with PFA. We used 4% paraformaldehyde (PFA) in PBS, freshly prepared from powder (Merck, Cat. No. 158127) and adjusted to pH 7.4.

3. How was the dissection of the brain carried out? Were the implants still inside the brain during brain sectioning? Or was the implant retrieved?
   We left the electrode bundles inside the brain in the animal (Animal#2) which was used for astrogliosis analysis. We nevertheless expect slight movements/displacements of electrode bundles during the dissection and sectioning.

   For the Animal#1 and Animal#3, we were not able to leave the electrodes in the brain as they were fully embedded in the bone (~5 months-long implantation). For these animals, we did electrolytic lesioning to localize electrode trajectories post-mortem.

   We now clarified these in the manuscript.

4. What is the "established protocol"? Please add references.

   The perfusion and fixation procedures are described in detail in our previous paper - Yasar et al. (2024) https://doi.org/10.1038/s41467-024-49226-9

   At the end of the in vivo experiments, the rat was euthanized via intraperitoneal injection of sodium pentobarbital (300 mg/kg; Esconarkon, Streuli Tiergesundheit AG). Once a deep level of anesthesia was confirmed, transcardial perfusion was performed using 4% paraformaldehyde (PFA) in phosphate-buffered saline (PBS). Following perfusion, the brain was carefully extracted from the skull, post-fixed overnight in 4% PFA, and then washed three times with 1× PBS.

   We now clarified these in the manuscript

5. Why did you section the brain into 100 μm-thick slices? Wouldn't thinner slices be a better option to provide better details?

   We sectioned the brain into 100 μm-thick slices to preserve the 3D tissue architecture. Thicker sections were particularly advantageous for analyzing spatial fluorescence gradients around implants (e.g., circular contour analysis around UFTEs, Fig. 5C), as they retained

more intact tissue surrounding the electrode sites. In contrast, thinner slices are more prone to mechanical damage and offer limited volumetric context due to tissue loss during sectioning.

High-resolution volumetric imaging was performed using the Olympus IXplore SpinSR Spinning-Disk Confocal system, equipped with long working distance, high-NA objectives, specifically the UPLXAPO 20x/0.80 air objective (working distance: 0.61 mm) and the UPLSAPO 30x/1.05 silicon objective (working distance: 0.8 mm). These optics enabled imaging through the full depth of the 100 µm-thick sections without compromising resolution from surface to deeper layers. Additionally, strong and efficient immunolabeling facilitated clear visualization across the entire section thickness.

To ensure uniform antibody penetration, we included a permeabilization step and extended the incubation time in antibody solutions to 2-3 days longer than in typical immunostaining protocols of thin brain slices.

We now clarified these in the manuscript.

6. What was the PBS concentration used? Was PBS self-made or was it acquired from a commercial supplier?

   We now clarified that we used 1× Phosphate Buffered Saline (PBS), pH 7.4 from Gibco (Thermo Fisher Scientific, Cat. No. 10010056).

7. Add details for suppliers and product numbers on all solutions, antibodies, etc.
   We have now added all the details for suppliers and product numbers in the answers to the reviewers questions regarding the immunostaining. We also added now all the corresponding details into the Methods section of the manuscript.

8. Are these free-floating stainings? Or directly on glass slides?

   We now clarified that all stainings were performed on free-floating brain sections. After staining, the sections were mounted onto standard microscopy glass slides (Superfrost Glass Microscope Slides with Ground Edges, Thermo Scientific Menzel-Gläser). Iohexol was used as the mounting medium, and the sections were covered with thin rectangular coverslips (Menzel-Gläser 24 × 60 mm, No. 1).

   Our immunohistological protocol is also described in the following publication (Methods section: “Immunohistological processing of the brain tissue”): Yasar, Gombkoto et al. 2024

9. Why was Nissl used instead of NeuN or MAP2, given that IHC was being carried out?
   We chose fluorescent Nissl staining (NeuroTrace) instead of NeuN or MAP2 because it was the most suitable option for our analysis of general neuron distribution and density around the UFTE implants. NeuroTrace labels Nissl substance found in the cell bodies of almost all neurons, giving a more complete and unbiased picture of neuronal populations. In comparison, NeuN labels only specific types of neurons and does not mark all neuronal populations (e.g misses some interneurons). MAP2 mainly stains dendrites, which was not useful for our analysis since we focused on counting and mapping neuron somata. NeuroTrace provided clear labeling of cell bodies, which was important for our measurements using concentric zones around the electrodes (Fig. 5C).

We greatly thank the reviewer for the very detailed and instructive comments and suggestions.

RESPONSE TO REVIEWERS

We thank Reviewer #1 for the 3 detailed comments and constructive feedback provided during the first round of revision, which helped us improve the quality of our manuscript. Reviewer #1 subsequently recommended our manuscript for publication in *Nature Communications*. We thank Reviewer #2 for the detailed comments and feedback, which likewise contributed substantially to strengthening the manuscript. We hope, over the last 6 months, we satisfactorily answered the 139 questions from Reviewer #2 in two revisions.

We extended the *Supplementary Figure 12* (Panel A) to elaborate MRID-based electrode localization results over several months, in response to Reviewer#2's 5th question. We replaced the bar plot in Figure 4D with a violin plot showing all the data points. The actual data behind the plot remained the same. We also made a few grammatical corrections and fixed typos in the main text to improve readability. Our responses to reviewers' comments and questions are highlighted in red font below. Changes in the manuscript are also highlighted in red. We have made small corrections to the main Figure 5B and Suppl. Figure 18, and corresponding statistical analysis (highlighted in the main text). We have corrected the n-numbers for some analyses and figures which we highlighted in the main text.

REVIEWER COMMENTS

Reviewer #1 (Remarks to the Author):

The authors have provided sufficient responses to my comments and have addressed the majority of them in the revised manuscript. Therefore, I find the revision satisfactory and recommend the manuscript for publication in Nature Communications.

Reviewer #2 (Remarks to the Author):

The revisions significantly improved the quality of the manuscript. However, a few open questions remain. Please see comments below:

1. Regarding the simulations and analytical calculations carried out for the critical wire length that could induce heating: Did you correct the Larmor frequency for the surrounding tissue? 1 m and 50 cm would correspond to the wavelength and half-wavelength resonance conditions if the medium were air. Which relative permittivity did you consider in your simulations? This is an important parameter, given the complex nature of the permittivity in biological tissue, which leads to frequency-dependent values that could range between 30 to 100, depending on which source you consult. If you

assume it to be 50, the half- and quarter-wavelengths are already 7 and 3.5 cm, respectively. Given that the electrodes are 6 mm long, what is the overall conductive length, including the feedlines connecting the electrodes to the pads (see also Suppl. Fig. 5. and Suppl. Fig. 20A, feedlines along polyimide implant), that may couple to RF fields and contribute to heating?

We thank the reviewer for bringing this important detail to our attention. In our COMSOL simulations, we used the realistic head phantom provided by COMSOL (https://www.comsol.com/model/specific-absorption-rate-sar-in-the-human-brain-2190) which is used for specific absorption rate simulations. The relative permittivity of the brain tissue was taken as 58.13 (i.e. similar to the value that the reviewer assumes). The gray matter permittivity is ~60 at 300Mhz according to literature resources (IT'IS Foundation Database Zurich, Gabriel et al. 1996, Pang et al. 2011).

epsilonr0_brain	58.13	58.13	Permittivity for the brain tissue

The overall conductive length is around 1.4cm outside the skull. However, during the implantation, we fold feedlines into 3-4 mm long segments and bury them under dental-cement to make sure there is no coiled-wire nor straight wire left (to spoil the RF resonance) on top of the skull.

Indeed, in the future, for human use or different animal models or different types of MRI scanners, electrode designs can be adapted according to the safety criteria as these parameters (e.g. feedline length) are flexible design choices.

We have now added these details to the manuscript.

2. Why were 2% agarose gel phantoms selected, instead of the typical 0.6% agarose w/w brain phantoms used to mimic the brain? What was the conductivity of the brain phantom used?
We used agarose gel phantoms solely for the imaging test, which is whether deposited iron-oxide nanoparticles (IONP) increase the visibility of ultra-flexible electrodes. 2% agarose gel phantom provided sufficient mechanical stability/rigidity to insert IONP-deposited and standard ultra-flexible electrodes into the phantom without forming air-bubbles for artifact-free MRI imaging.
Moreover, the T2*-relaxation time of 2% agarose gel phantom is shorter (~65 ms) compared to 0.6% agarose gel phantom (~80 ms) according to our measurements. Therefore, its value is closer to the rat brain T2*-relaxation time (~50 ms, Cremillieux et al. 1998) compared to that of the 0.6% agarose gel phantom.

The conductivity of the brain phantom here was not important because we did not carry out any impedance measurements inside the agarose gel phantom.

3. Long-term stability: It seems that the impedance median is getting lower across months (nearly half after 6 months compared to 1-month post-implantation, see NEW Rebuttal Figure. Chronic stability example of an MRID-tagged UFTE, panel C), and it also seems that the contrast has a negative trend. Could this be an early indication of water intrusion between the layers (not necessarily delamination per se)?

   We thank the reviewer for this important comment. Indeed, this alone could indicate water intrusion between layers. However, we think this is unlikely because signal quality (e.g. SNR) remained stable e.g. 16.49 ± 1.47 (s.e.m) to 20.38 ± 2.08 (s.e.m) for Animal#1 over 23 weeks; 25.33 ± 2.15 (s.e.m) to 26.02 ± 2.00 (s.e.m) for Animal#3 over 22 weeks.

4. Regarding the question about possible electrode movement over time. Where can I find Suppl. Fig 6.1 and 6.2?

   We thank the reviewer for bringing this to our attention. We apologize for not updating the index of supplementary figures. *Supplementary Figure 6.1* corresponds to *Supplementary Figure 12* in the final submission. *Supplementary Figure 6.2* corresponds to *Supplementary Figure 17* in the final submission.

5. According to New Suppl. Fig. “Localization differences between MRID electrode-based localization...”, localization drifts of up to 150 μm can be observed. While electrodes would still be in CA1, they could be moving across layers within the CA1. Is this a correct interpretation? According to Fig.5B, the electrodes do move.

   We used electrophysiological landmarks as the ground-truth reference for localization, as the CA1 pyramidal layer is sharply and reliably defined by electrophysiological markers. The CA1 pyramidal layer, identified by the channel with maximal ripple power, showed minimal variation: bundles 1 and 3 showed no detectable variation (0 μm) while bundle 2 varied within ±50 μm, with the same channels consistently identified over extended periods (bundles 1 and 2 for 6 months; bundle 3 for 5 months).

   The differences of up to ~150 μm observed between MRID- and electrophysiology-based localization reflect methodological uncertainty inherent to mostly MRI detection, rather than physical movement of electrodes across CA1 layers. To make this explicit, we now provide separate analyses (Supplementary Fig. 12, please see below) showing month-to-month variation for each method. The lack of electrode movement is further supported by the high stability of waveforms from many single units recorded at the same contact sites within the bundle over multiple months, both in the present study and in our previous UFTE-based work.

(In contrast to the CA1 pyramidal layer identified by maximum ripple power, electrophysiological localization of the hippocampal fissure was less reliable, as it depends on strong theta power, which predominates during exploration. With habituation, theta activity becomes weaker and shorter in duration, reducing detection reliability and necessitating sufficiently strong theta for accurate HF identification.)

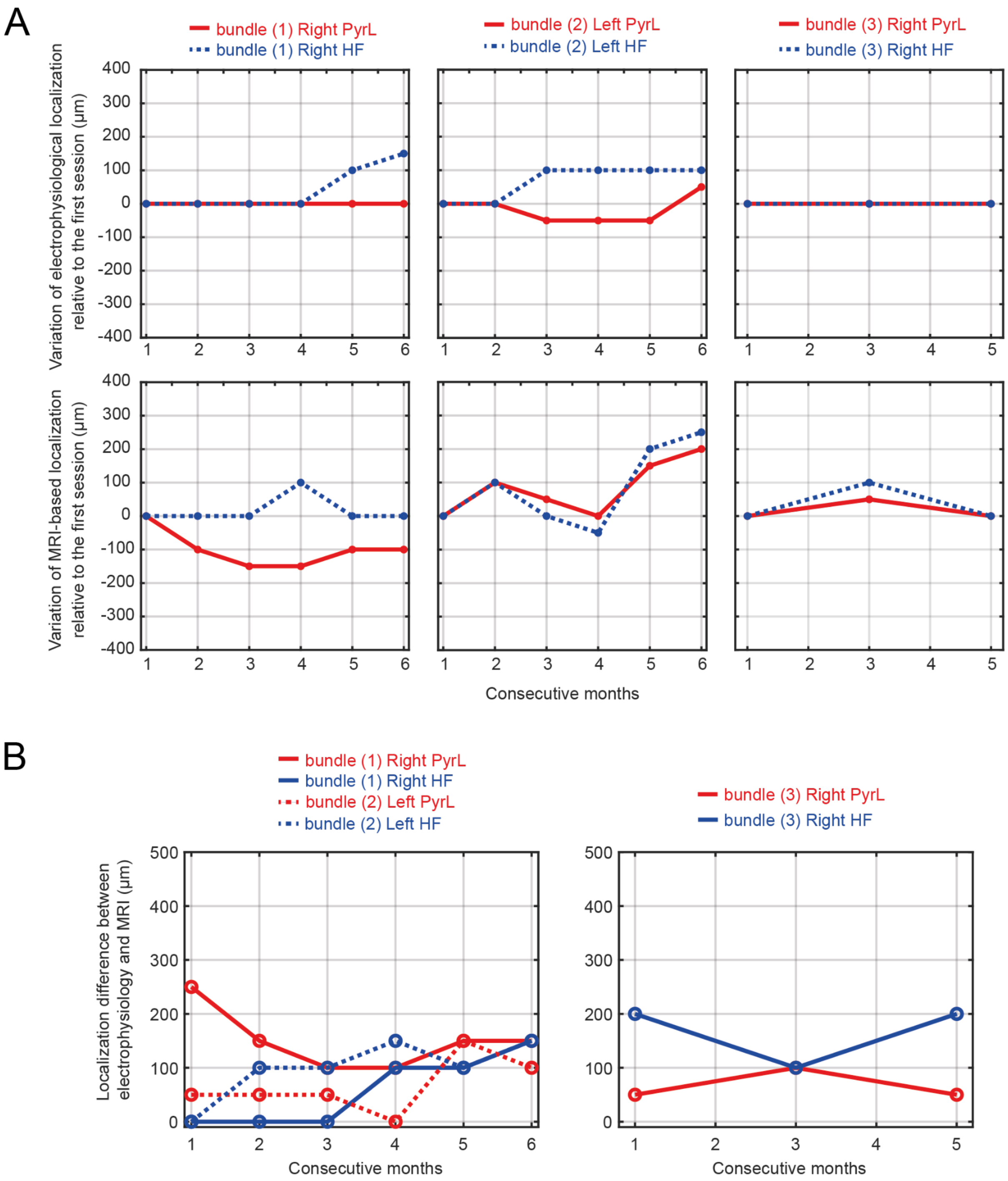

Supplementary Fig. 12.
A. *Top row:* Three representative electrode bundles showing localization consistency across sessions based on electrophysiological (LFP-derived) landmarks, referenced to the first

recording session (0 μm). The solid red line marks the CA1 pyramidal layer, and the dashed blue line indicates the hippocampal fissure at each measurement time point. *Bottom row:* Variation of consecutive MRI-based localization across sessions relative to the first session (0 μm reference). B. Localization differences between MRID–based localization and electrophysiological landmarks derived from LFPs, including the CA1 pyramidal layer (red) and the hippocampal fissure (blue). Solid lines denote the right hemisphere, and dashed lines denote the left.
